# Supplementary material for: Inositol Derivatives with Anti-Inflammatory Activity from Leaves of Solanum capsicoides Allioni
Source: Molecules. 2022 Sep 16;27(18):6063. doi: 10.3390/molecules27186063 (PMC9503535; doi:10.3390/molecules27186063)
Supplement: Supplementary file 1 [file molecules-27-06063-s001.zip › molecules-1912371-supplementary.pdf]

molecules

Supplementary Material

Inositol derivatives with anti-inflammatory activity from leaves of *Solanum capsicoides* Allioni

Yan Liu, Xin Meng, Han Wang, Yan Sun, Si-Yi Wang, Yi-Kai Jiang, Adnan Mohammed Algradi, Anam Naseem, Hai-Xue Kuang* and Bing-You Yang*

Key Laboratory of Basic and Application Research of Beiyao (Heilongjiang University of Chinese Medicine), Ministry of Education, Heilongjiang University of Chinese Medicine, Harbin 150040, China.

* Correspondence: hxkuang@yahoo.com (H.-X.K.); ybywater@163.com (B.-Y.Y.)

Figure S1. HR-ESI-MS spectrum of **1**

Figure S2. ^{1}H NMR spectrum (AV-600, 600 MHz) of compound **1** in CD_3OD-*d4*

Figure S3. ^{13}C NMR spectrum (AV-600, 150 MHz) of compound **1** in CD_3OD-*d4*

Figure S4. DEPT spectrum (AV-600) of compound **1** in CD_3OD-*d4*

Figure S5. ^{1}H-^{1}H COSY spectrum (AV-600) of compound **1** in CD_3OD-*d4*

Figure S6. HSQC spectrum (AV-600) of compound **1** in CD_3OD-*d4*

Figure S7. HMBC spectrum (AV-600) of compound **1** in CD_3OD-*d4*

Figure S8. NOESY spectrum (AV-600) of compound **1** in CD_3OD-*d4*

Figure S9. HR-ESI-MS spectrum of Compound **2**

Figure S10. ^{1}H NMR spectrum (AV-600, 600 MHz) of compound **2** in CD_3OD-*d4*

Figure S11. ^{13}C-NMR spectrum (AV-600, 150 MHz) of compound **2** in CD_3OD-*d4*

Figure S12. DEPT spectrum (AV-600) of compound **2** in CD_3OD-*d4*

Figure S13. ^{1}H-^{1}H COSY spectrum (AV-600) of compound **2** in CD_3OD-*d4*

Figure S14. HSQC spectrum (AV-600) of compound **2** in CD_3OD-*d4*

Figure S15. HMBC spectrum (AV-600) of compound **2** in CD_3OD-*d4*

Figure S16. NOESY spectrum (AV-600) of compound **2** in CD_3OD-*d4*

Figure S17. HR-ESI-MS spectrum of compound **3**

Figure S18. ^{1}H-NMR spectrum (AV-600, 600 MHz) of compound **3** in CD_3OD-*d4*

Figure S19. ^{13}C NMR spectrum (AV-600, 150 MHz) of compound **3** in CD_3OD-*d4*

Figure S20. DEPT spectrum (AV-600) of compound **3** in CD_3OD-*d4*

Figure S21. ^{1}H-^{1}H COSY spectrum (AV-600) of **3** in CD_3OD-*d4*

Figure S22. HSQC spectrum (AV-600) of **3** in CD_3OD-*d4*

Figure S23. HMBC spectrum (AV-600) of **3** in CD_3OD-*d4*

Figure S24. NOESY spectrum (AV-600) of **3** in CD_3OD-*d4*

Figure S25. HR-ESI-MS spectrum of **4** in CD_3OD-*d4*

Figure S26. ^{1}H NMR spectrum (AV-600, 600 MHz) of **4** in CD_3OD-*d4*

Figure S27. ^{13}C NMR spectrum (AV-600, 150 MHz) of **4** in CD_3OD-*d4*

Figure S28. DEPT spectrum (AV-600) of **4** in CD_3OD-*d4*

Figure S29. ^{1}H-^{1}H COSY spectrum (AV-600) of **4** in CD_3OD-*d4*

Figure S30. HSQC spectrum (AV-600) of **4** in CD_3OD-*d4*

Figure S31. HMBC spectrum (AV-600) of **4** in CD_3OD-*d4*

Figure S32. NOESY spectrum (AV-600) of **4** in CD_3OD-*d4*

Figure S33. HR-ESI-MS spectrum of **5** in CD_3OD-*d4*

Figure S34. ^{1}H NMR spectrum (AV-600, 600 MHz) of **5** in CD_3OD-*d4*

Figure S35.^{13}C NMR spectrum (AV-600, 150 MHz) of **5** in CD_3OD-*d4*

Figure S36. DEPT spectrum (AV-600) of **5** in CD_3OD-*d4*

Figure S37. ^{1}H-^{1}H COSY spectrum (AV-600) of **5** in CD_3OD-*d4*

Figure S38. HSQC spectrum (AV-600) of **5** in CD_3OD-*d4*

Figure S39. HMBC spectrum (AV-600) of **5** in CD_3OD-*d4*

Figure S40. NOESY spectrum (AV-600) of **5** in CD_3OD-*d4*

Figure S41. HR-ESI-MS spectrum of **6** in CD_3OD-*d4*

Figure S42. ^{1}H NMR spectrum (AV-600, 600 MHz) of **6** in CD_3OD-*d4*

Figure S43. ^{13}C NMR spectrum (AV-600, 150 MHz) of **6** in CD_3OD-*d4*

Figure S44. DEPT spectrum (AV-600) of **6** in CD_3OD-*d4*

Figure S45. ^{1}H-^{1}H COSY spectrum (AV-600) of **6** in CD_3OD-*d4*

Figure S46. HSQC spectrum (AV-600) of **6** in CD_3OD-*d4*

Figure S47. HMBC spectrum (AV-600) of **6** in CD_3OD-*d4*

Figure S48. NOESY spectrum (AV-600) of **6** in CD_3OD-*d4*

Figure S49. HR-ESI-MS spectrum of **7** in CD_3OD-*d4*

Figure S50. ^{1}H NMR spectrum (AV-600, 600 MHz) of **7** in CD_3OD-*d4*

Figure S51.^{13}C NMR spectrum (AV-600, 150 MHz) of **7** in CD_3OD-*d4*

Figure S52. DEPT spectrum (AV-600) of **7** in CD_3OD-*d4*

Figure S53. ^{1}H-^{1}H COSY spectrum (AV-600) of **7** in CD_3OD-*d4*

Figure S54. HSQC spectrum (AV-600) of **7** in CD_3OD-*d4*

Figure S55. HMBC spectrum (AV-600) of **7** in CD_3OD-*d4*

Figure S56. NOESY spectrum (AV-600) of **7** in CD_3OD-*d4*

Figure S57. HR-ESI-MS spectrum of **8** in CD_3OD-*d4*

Figure S58. ^{1}H NMR spectrum (AV-600, 600 MHz) of **8** in CD_3OD-*d4*

Figure S59.^{13}C NMR spectrum (AV-600, 150 MHz) of **8** in CD_3OD-*d4*

Figure S60. DEPT spectrum (AV-600) of **8** in CD_3OD-*d4*

Figure S61. ^{1}H-^{1}H COSY spectrum (AV-600) of **8** in CD_3OD-*d4*

Figure S62. HSQC spectrum (AV-600) of **8** in CD_3OD-*d4*

Figure S63. HMBC spectrum (AV-600) of **8** in CD_3OD-*d4*

Figure S64. NOESY spectrum (AV-600) of **8** in CD_3OD-*d4*

Figure S65. HPLC spectrum of **1**–**8**

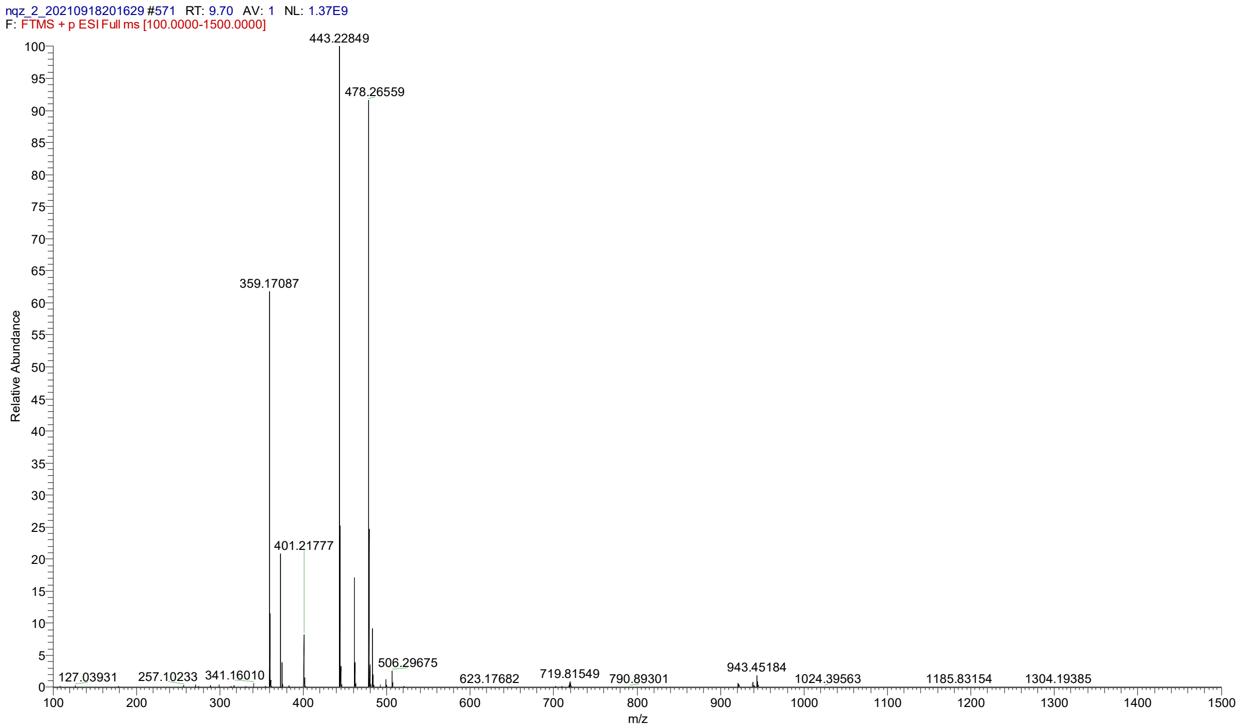

Figure S1. HR-ESI-MS spectrum of **1**

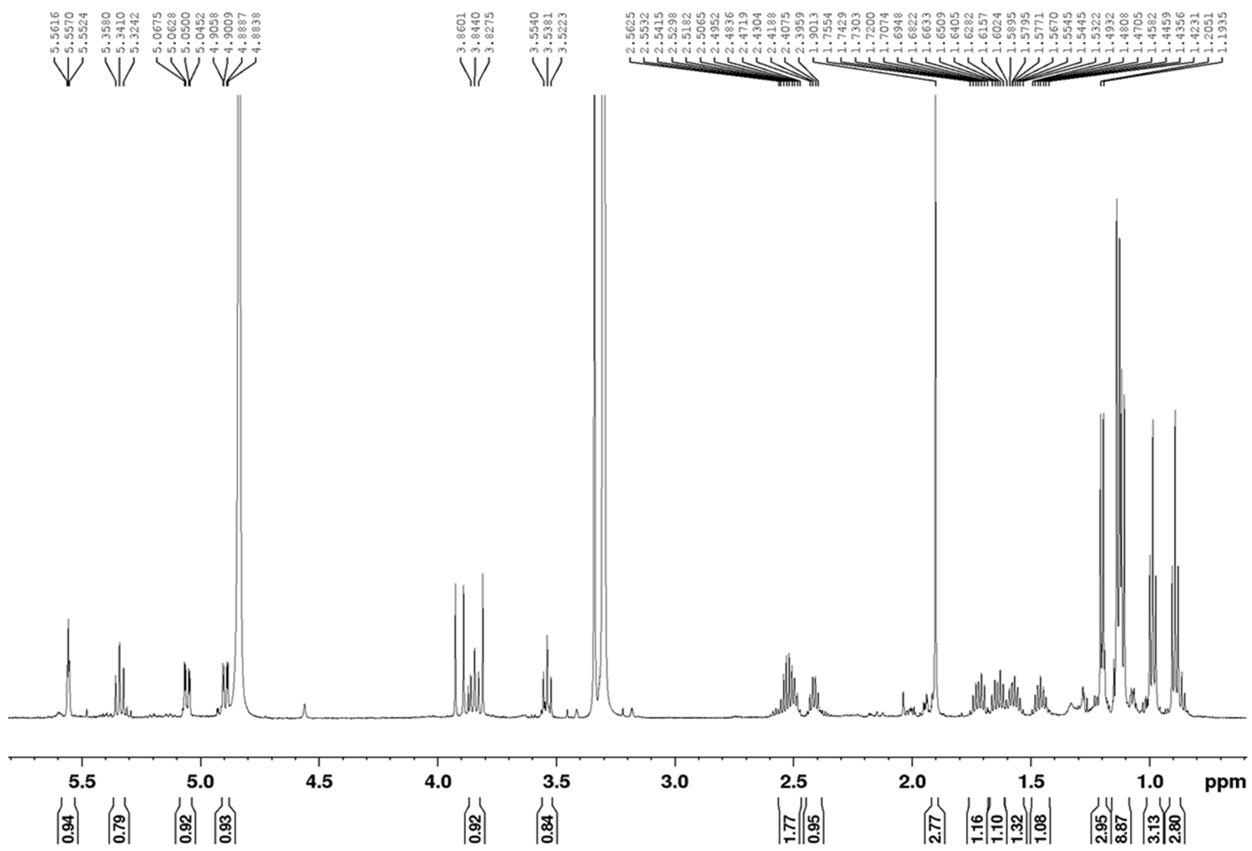

Figure S2. ^{1}H NMR spectrum (AV-600, 600 MHz) of compound **1** in CD_3OD-*d4*

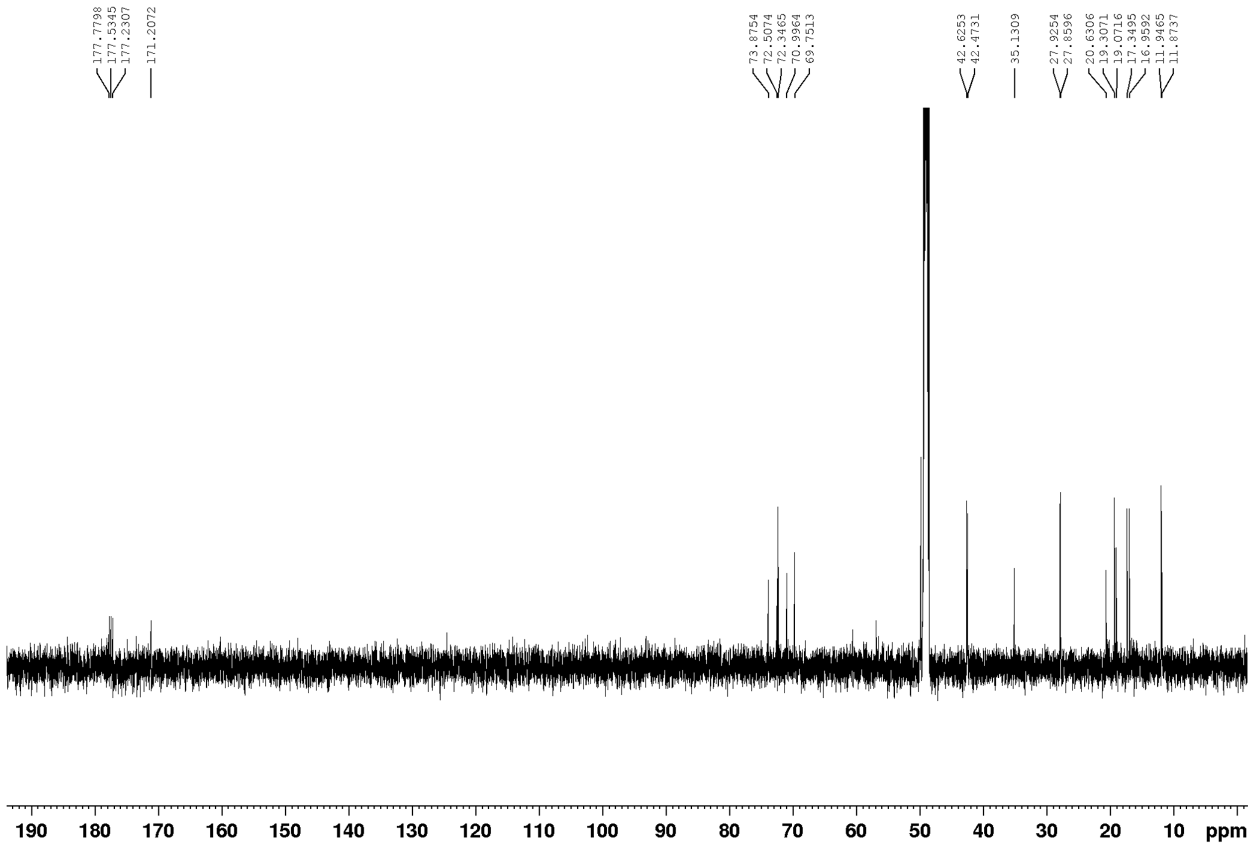

Figure S3. ^{13}C NMR spectrum (AV-600, 150 MHz) of compound **1** in CD_3OD-*d4*

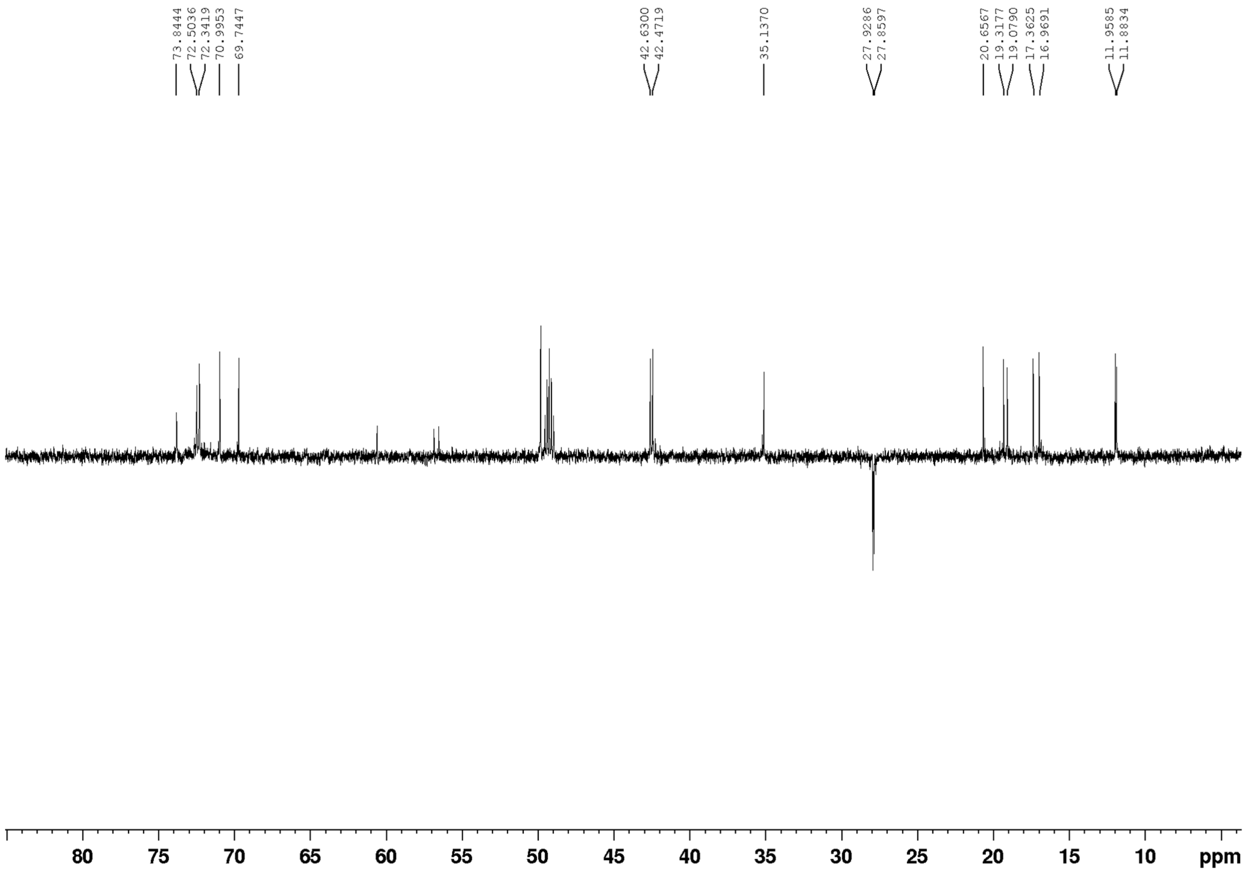

Figure S4. DEPT spectrum (AV-600) of compound **1** in CD_3OD-*d4*

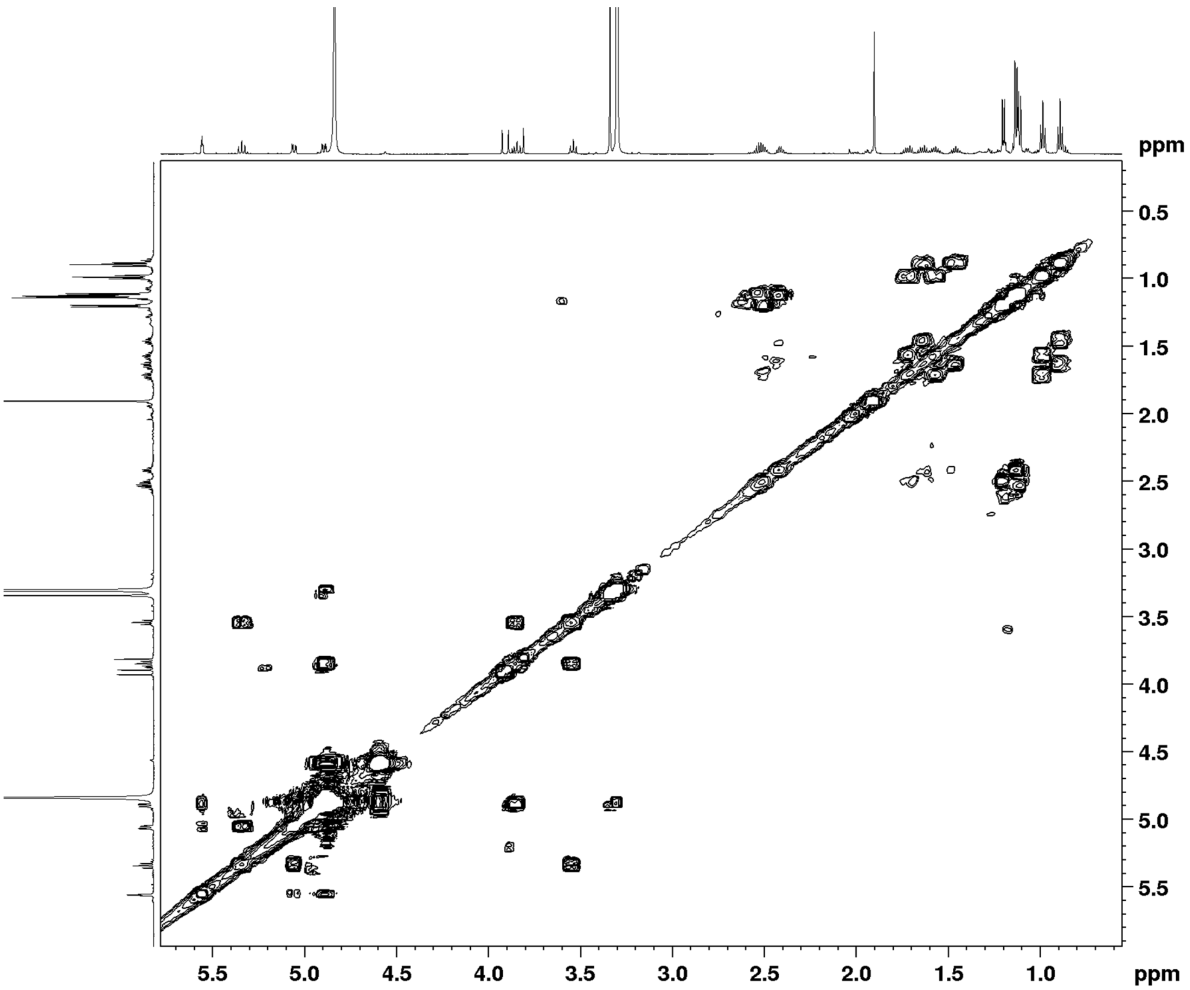

Figure S5. ^{1}H-^{1}H COSY spectrum (AV-600) of compound **1** in CD_3OD-*d4*

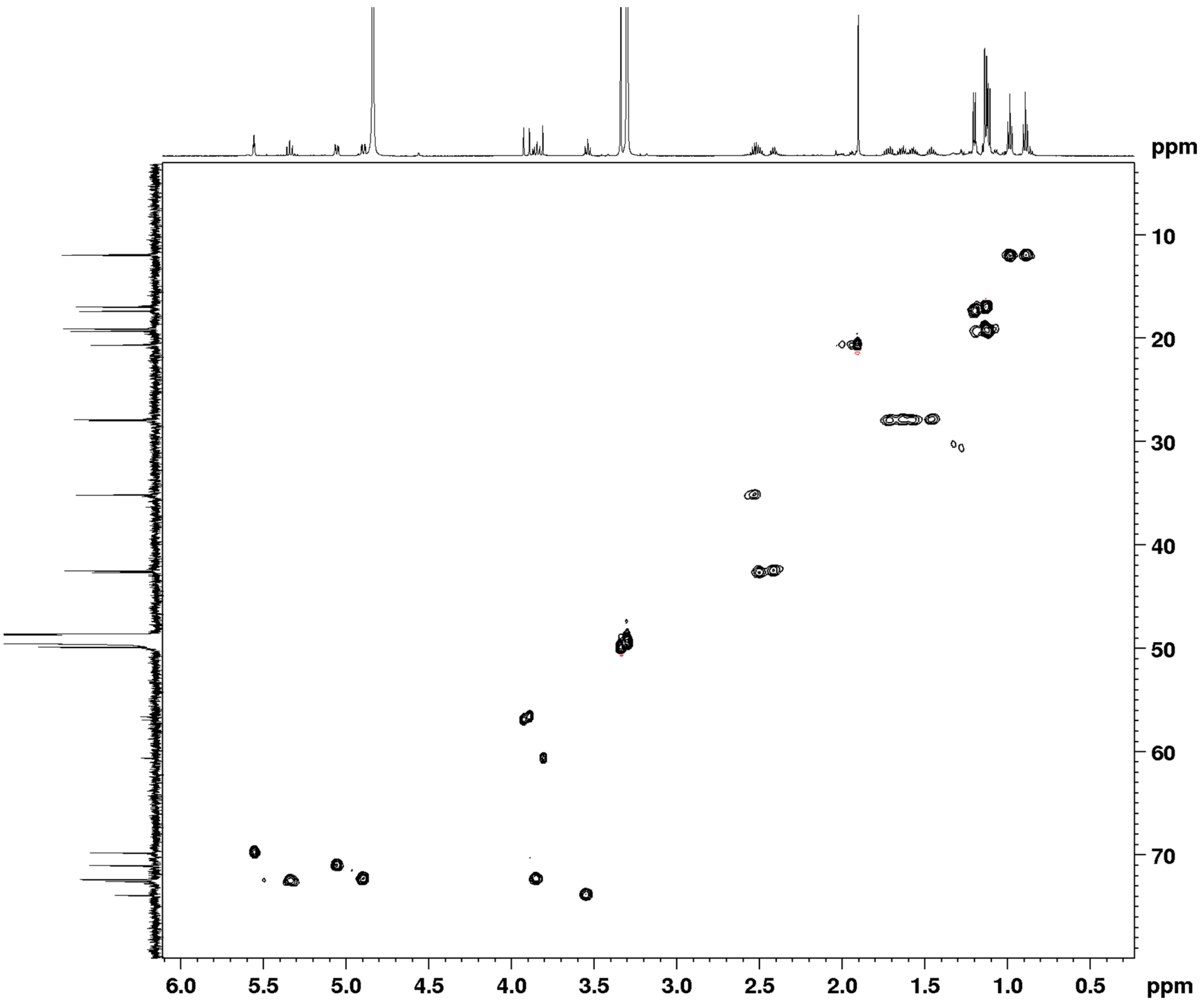

Figure S6. HSQC spectrum (AV-600) of compound **1** in CD3OD-*d4*

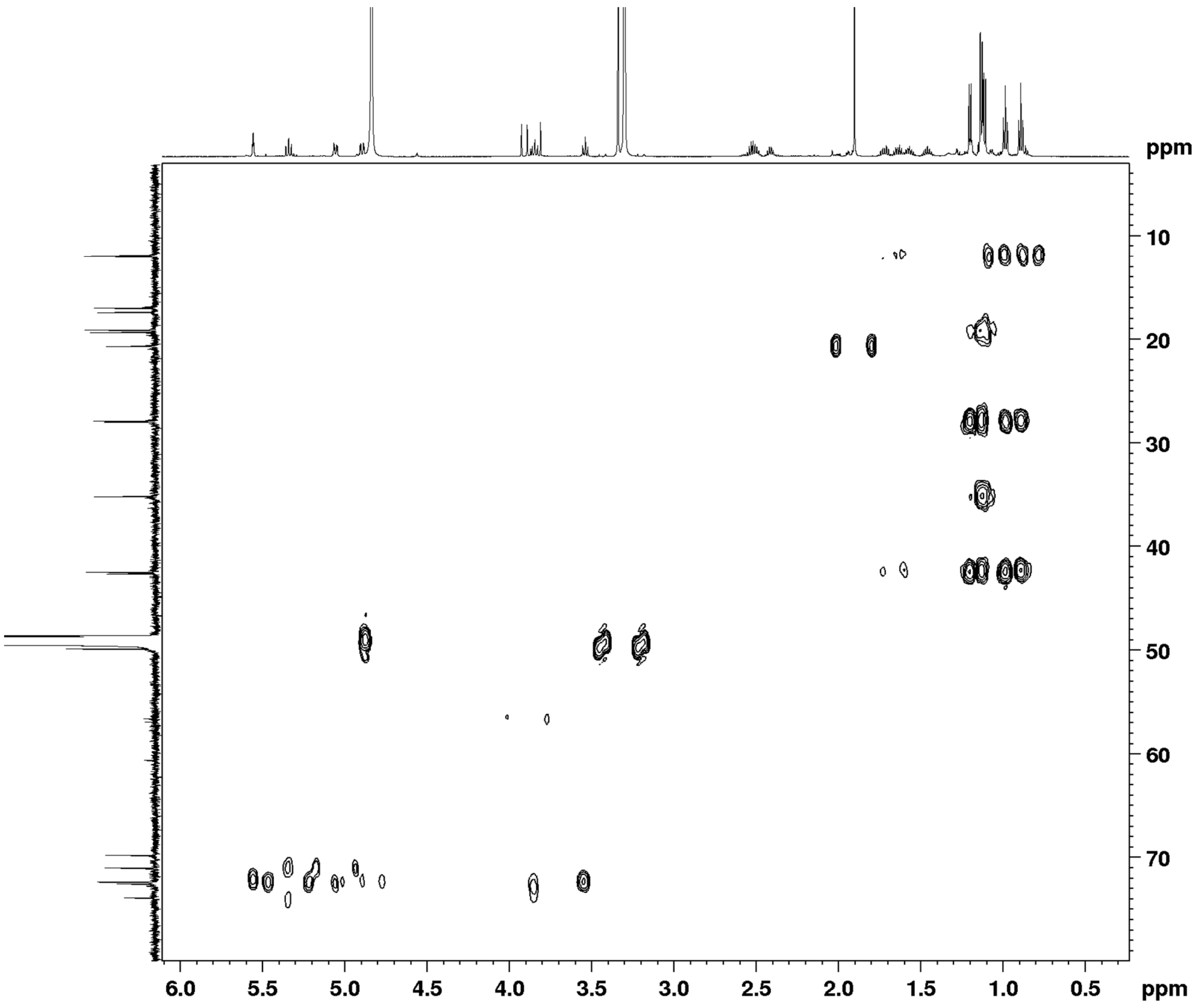

Figure S7. HMBC spectrum (AV-600) of compound **1** in CD_3OD-*d4*

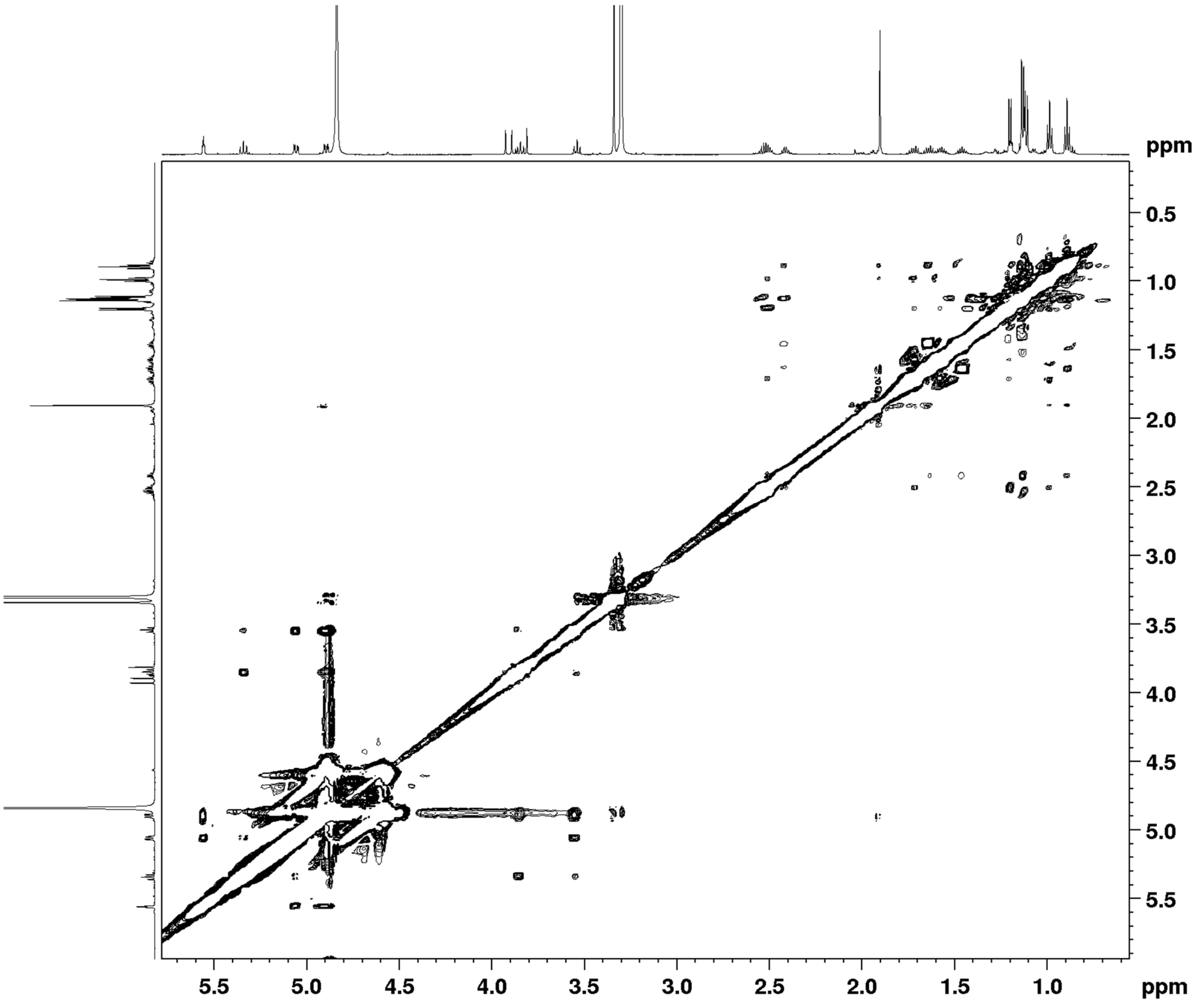

Figure S8. NOESY spectrum (AV-600) of compound **1** in CD_3OD-*d4*

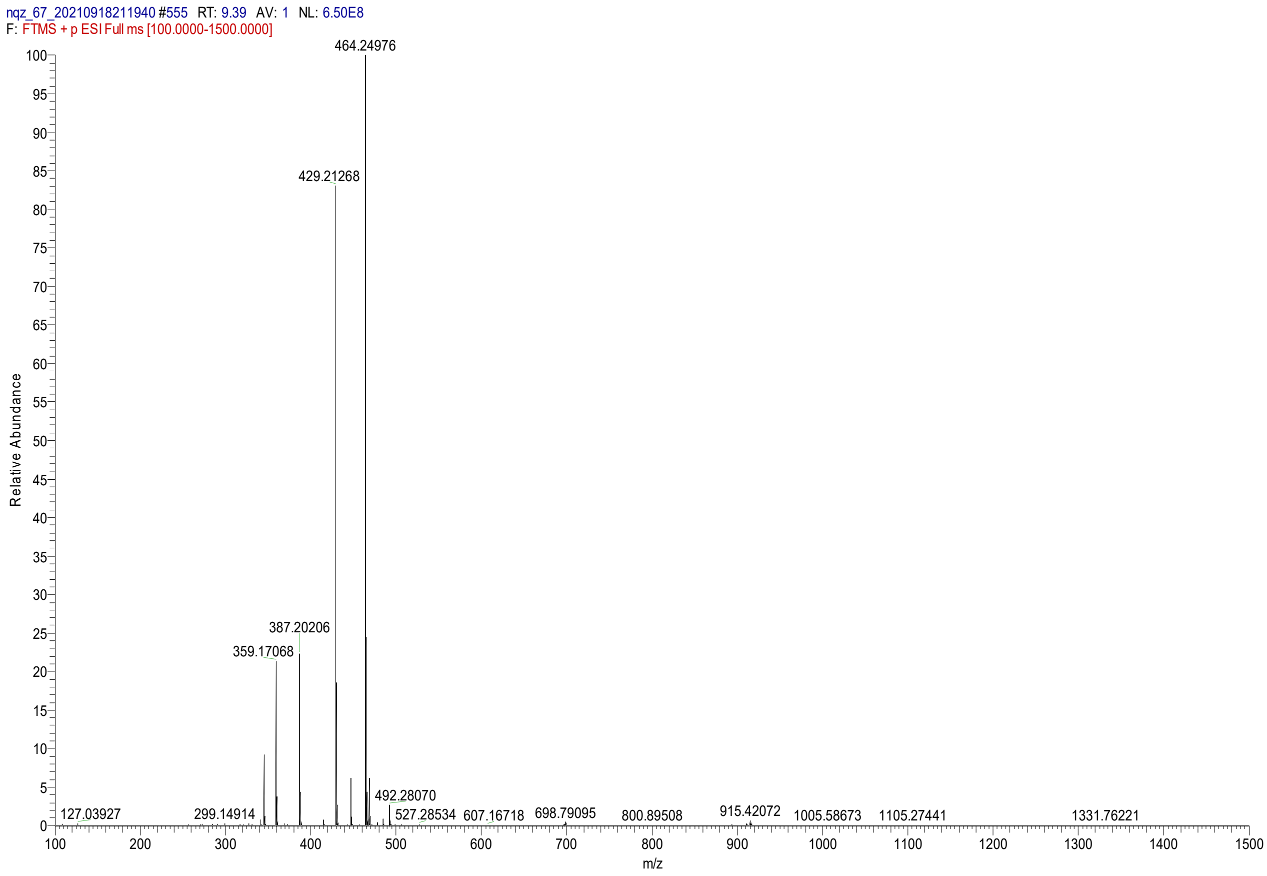

Figure S9. HR-ESI-MS spectrum of Compound **2**

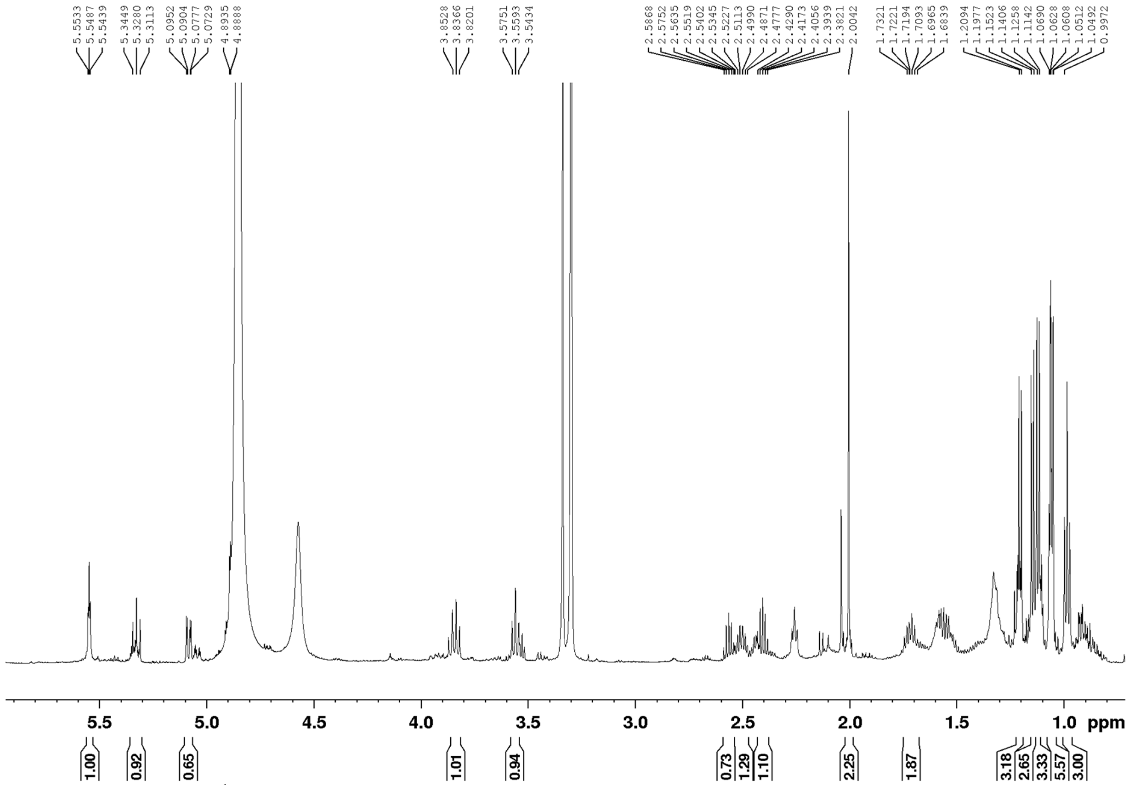

Figure S10. ^1H NMR spectrum (AV-600, 600 MHz) of compound **2** in CD_3OD-*d4*

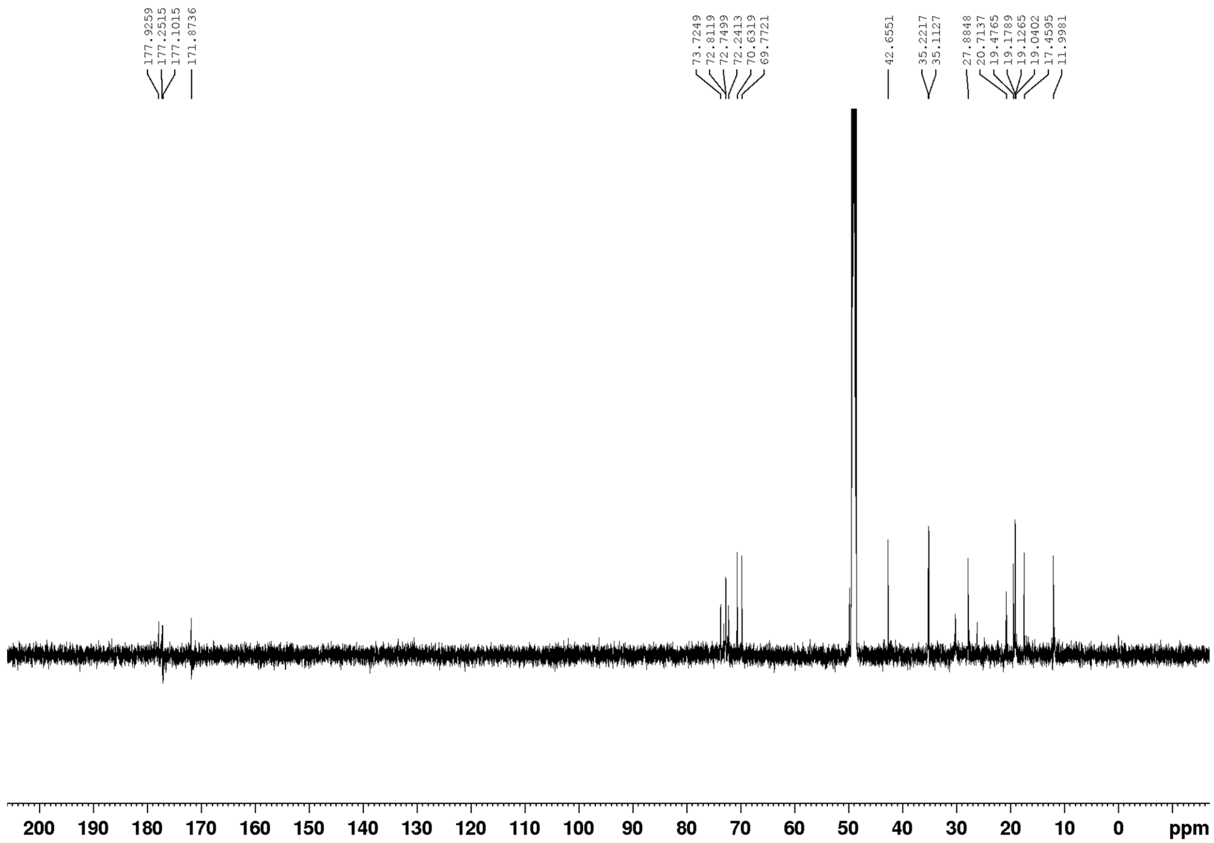

Figure S11. ^{13}C-NMR spectrum (AV-600, 150 MHz) of compound **2** in CD_3OD-*d4*

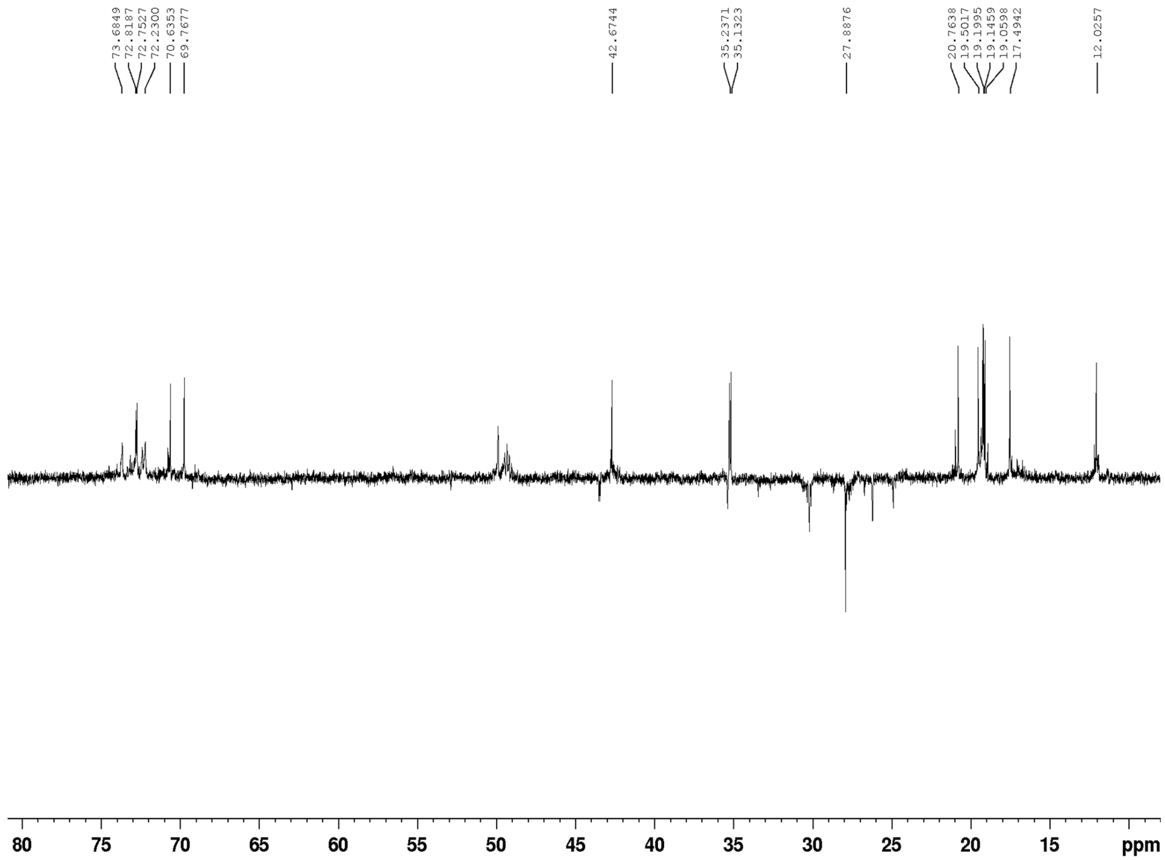

Figure S12. DEPT spectrum (AV-600) of compound **2** in CD_3OD-*d4*

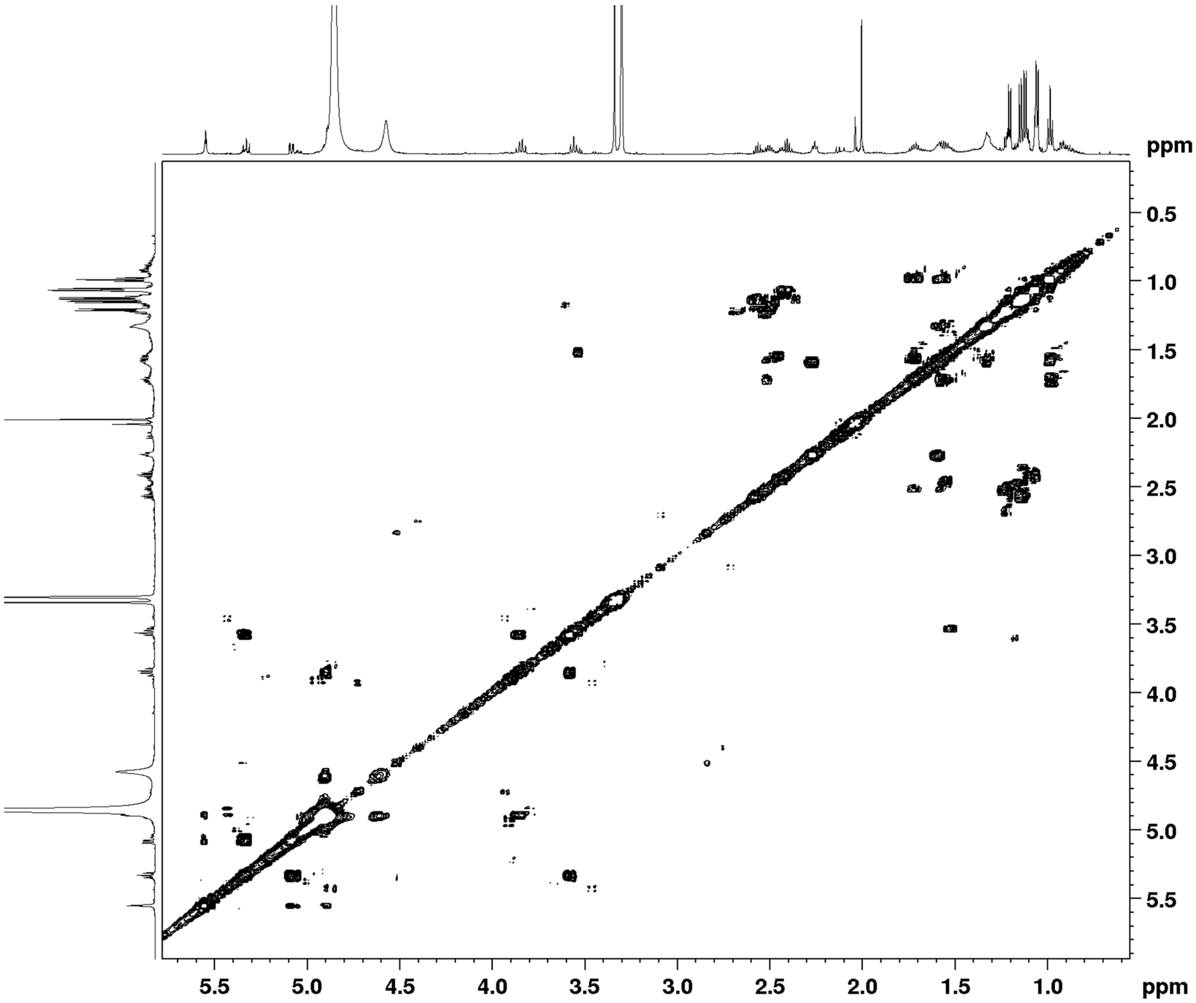

Figure S13. ^1H-^1H COSY spectrum (AV-600) of compound **2** in CD_3OD-*d4*

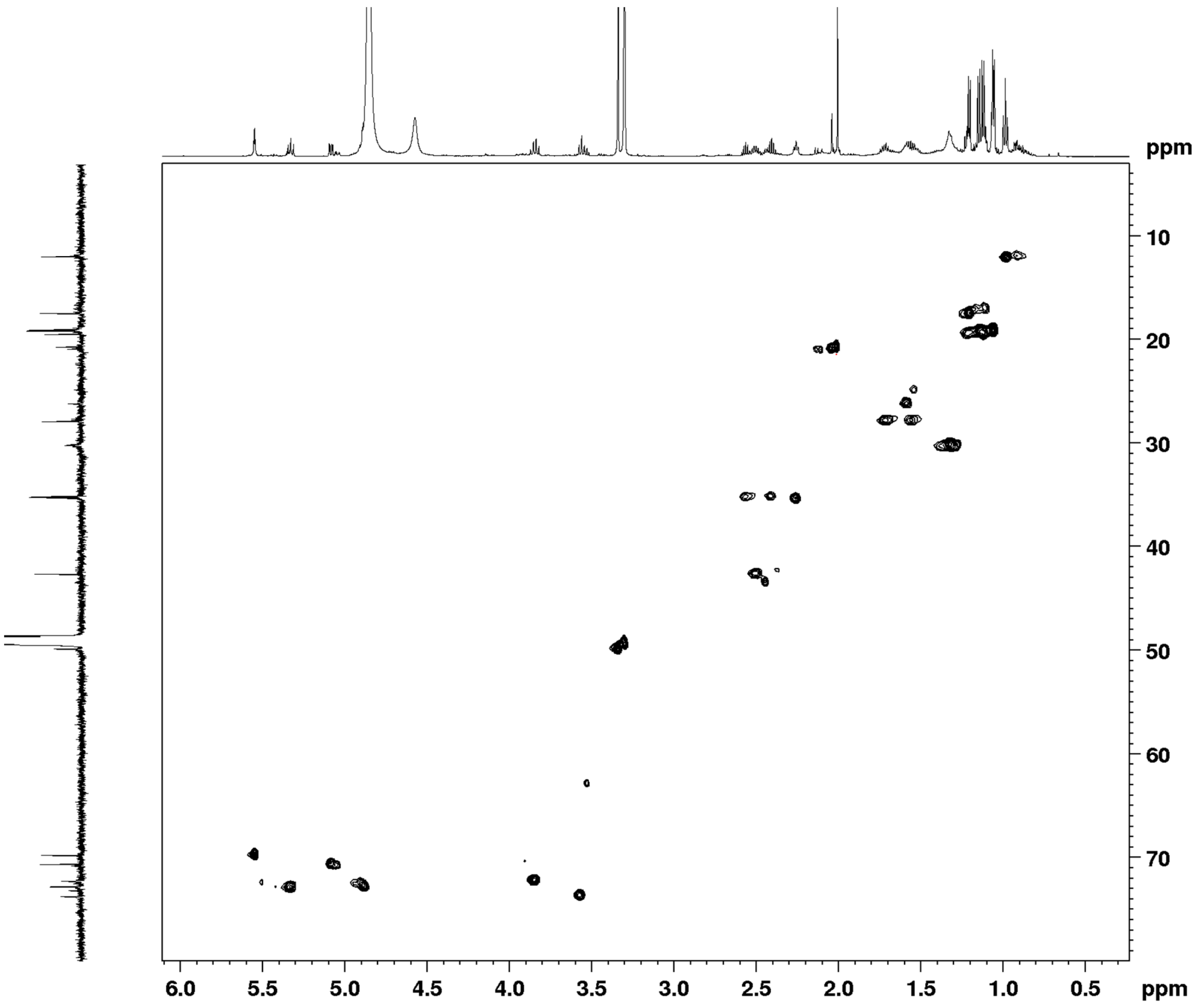

Figure S14. HSQC spectrum (AV-600) of compound **2** in CD_3OD-*d4*

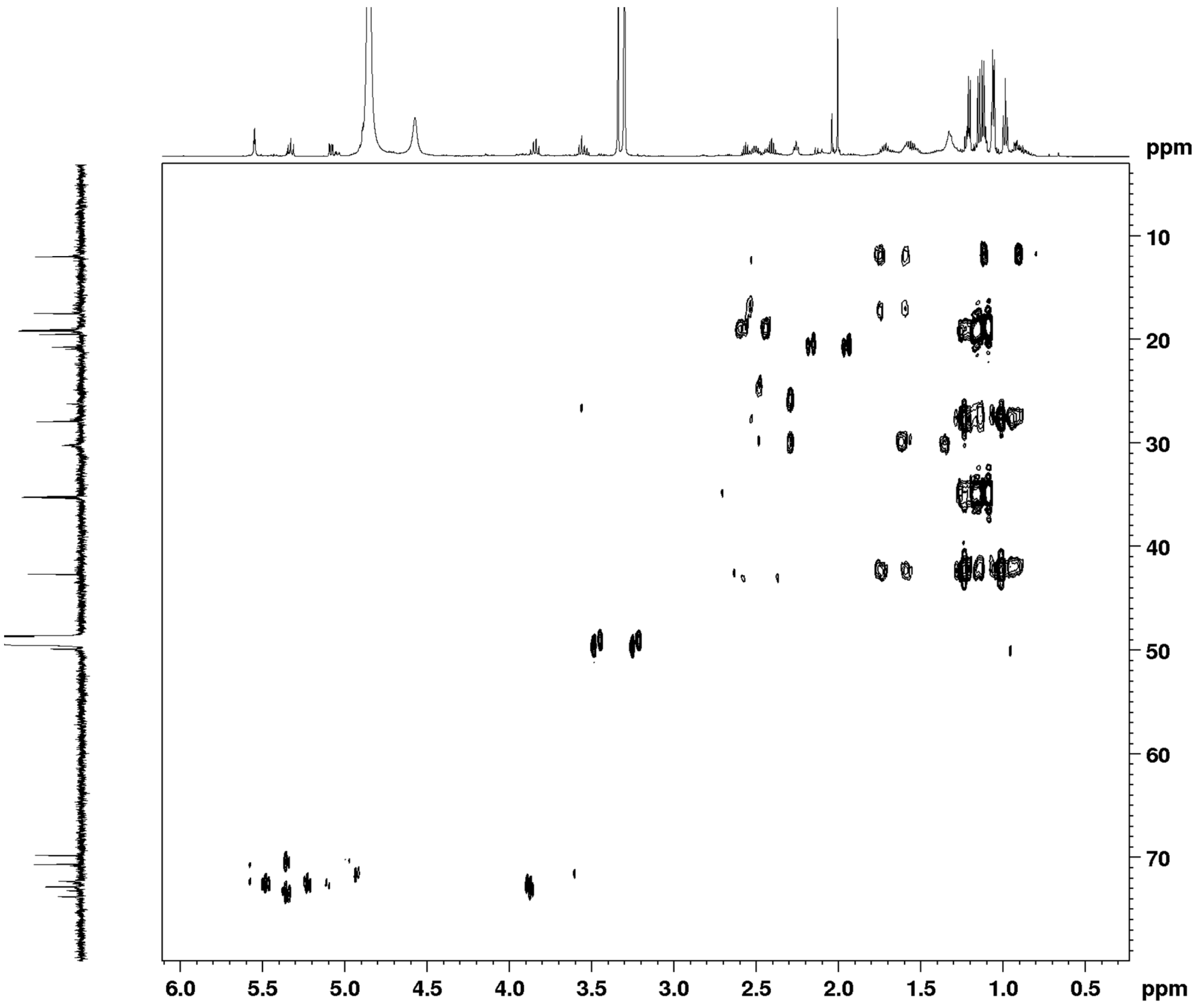

Figure S15. HMBC spectrum (AV-600) of compound **2** in CD_3OD-*d4*

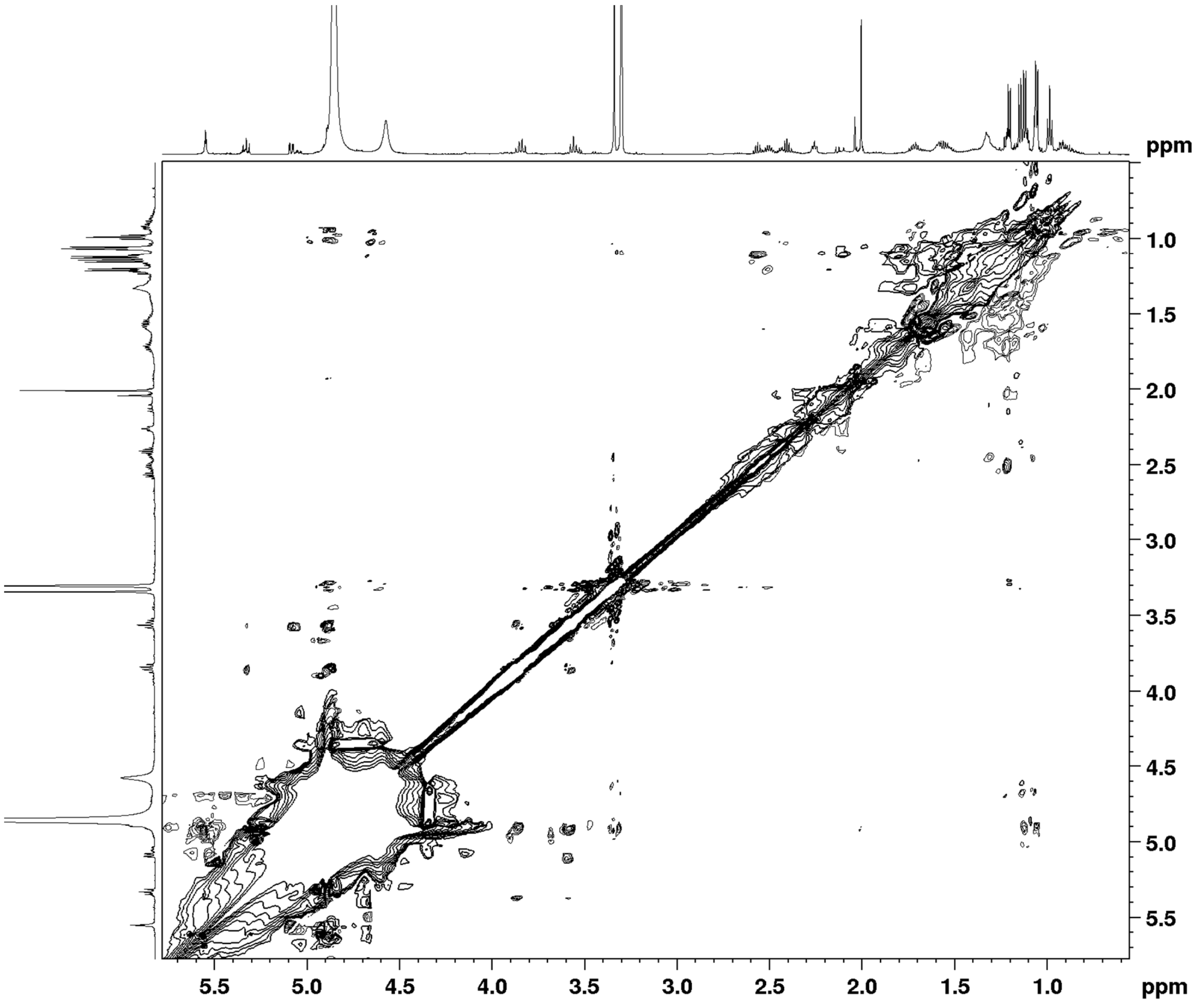

Figure S16. NOESY spectrum (AV-600) of compound **2** in CD_3OD-*d4*

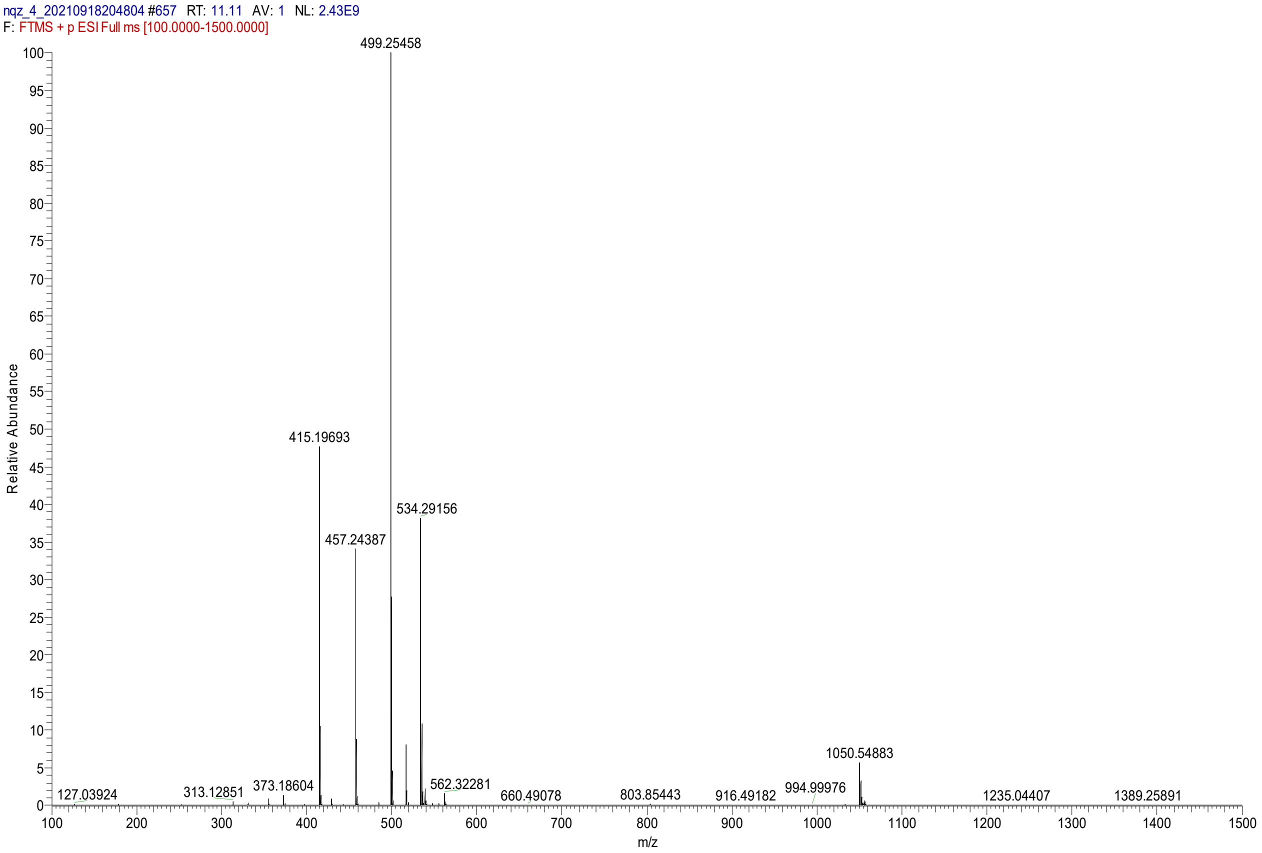

Figure S17. HR-ESI-MS spectrum of compound **3**

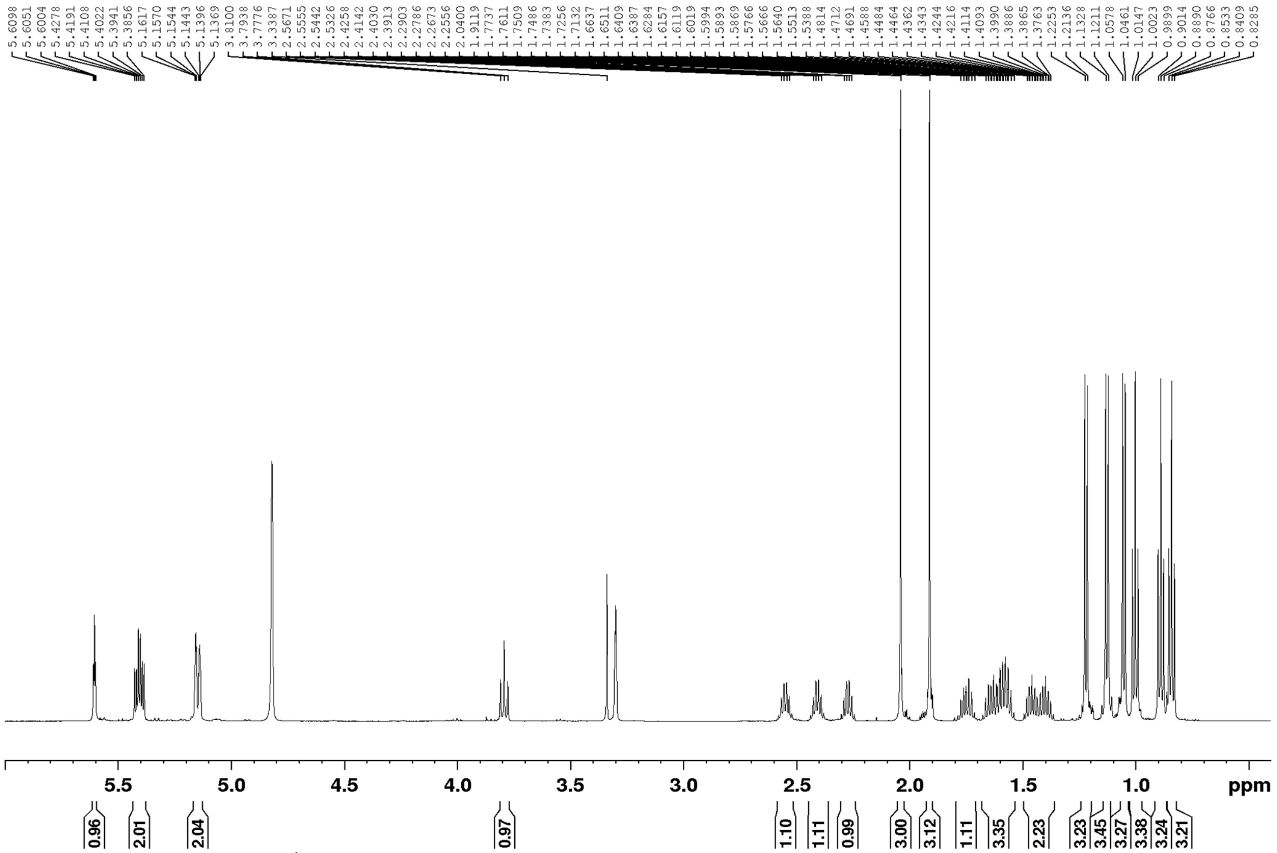

Figure S18. ^{1}H-NMR spectrum (AV-600, 600 MHz) of compound **3** in CD3OD-*d4*

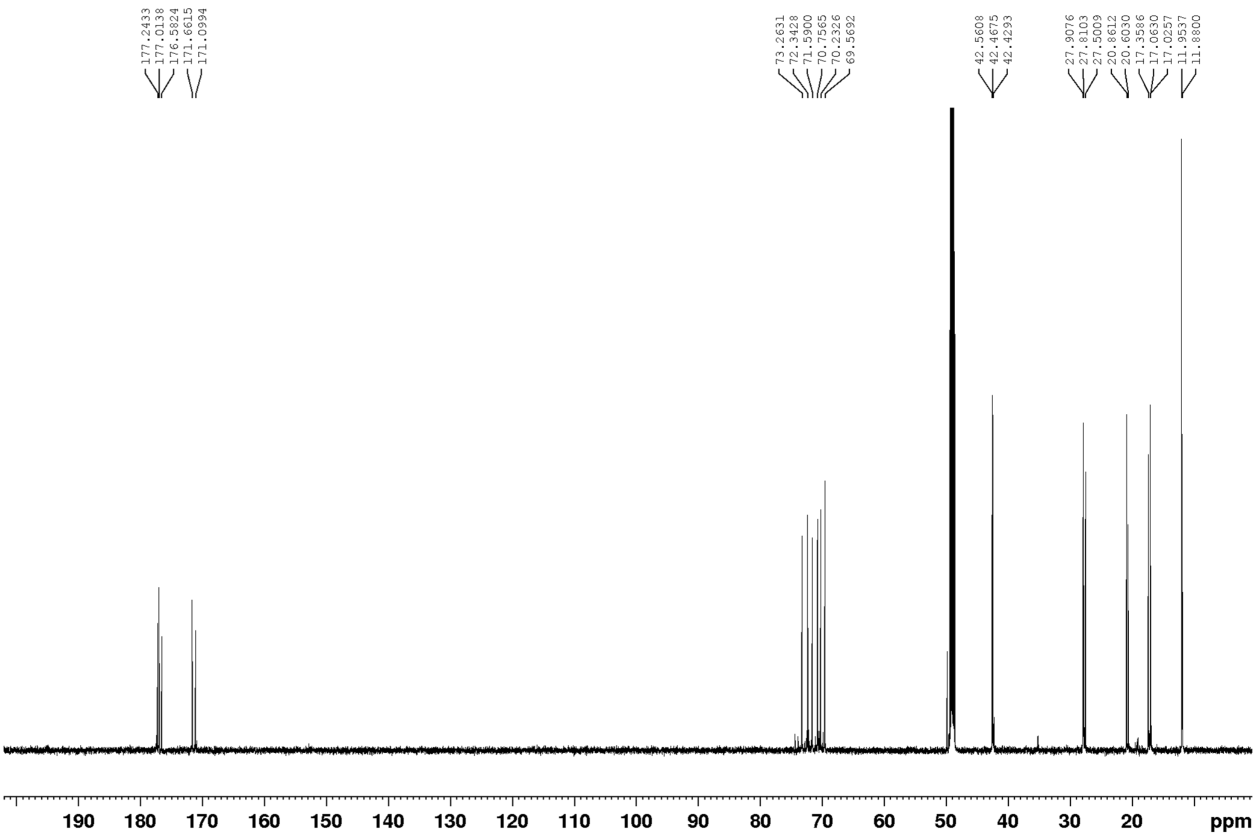

Figure S19. ^{13}C NMR spectrum (AV-600, 150 MHz) of compound **3** in CD_3OD-*d4*

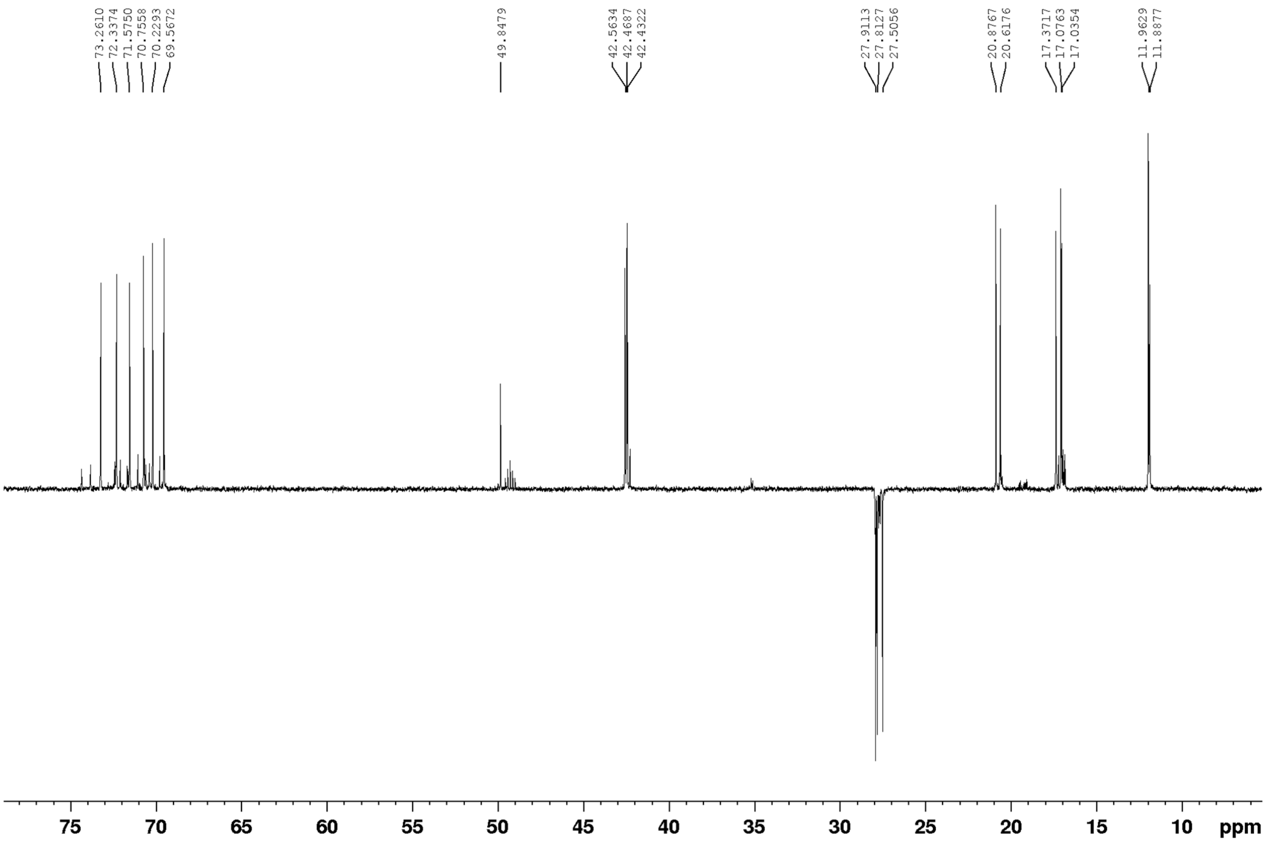

Figure S20. DEPT spectrum (AV-600) of compound **3** in CD_3OD-*d4*

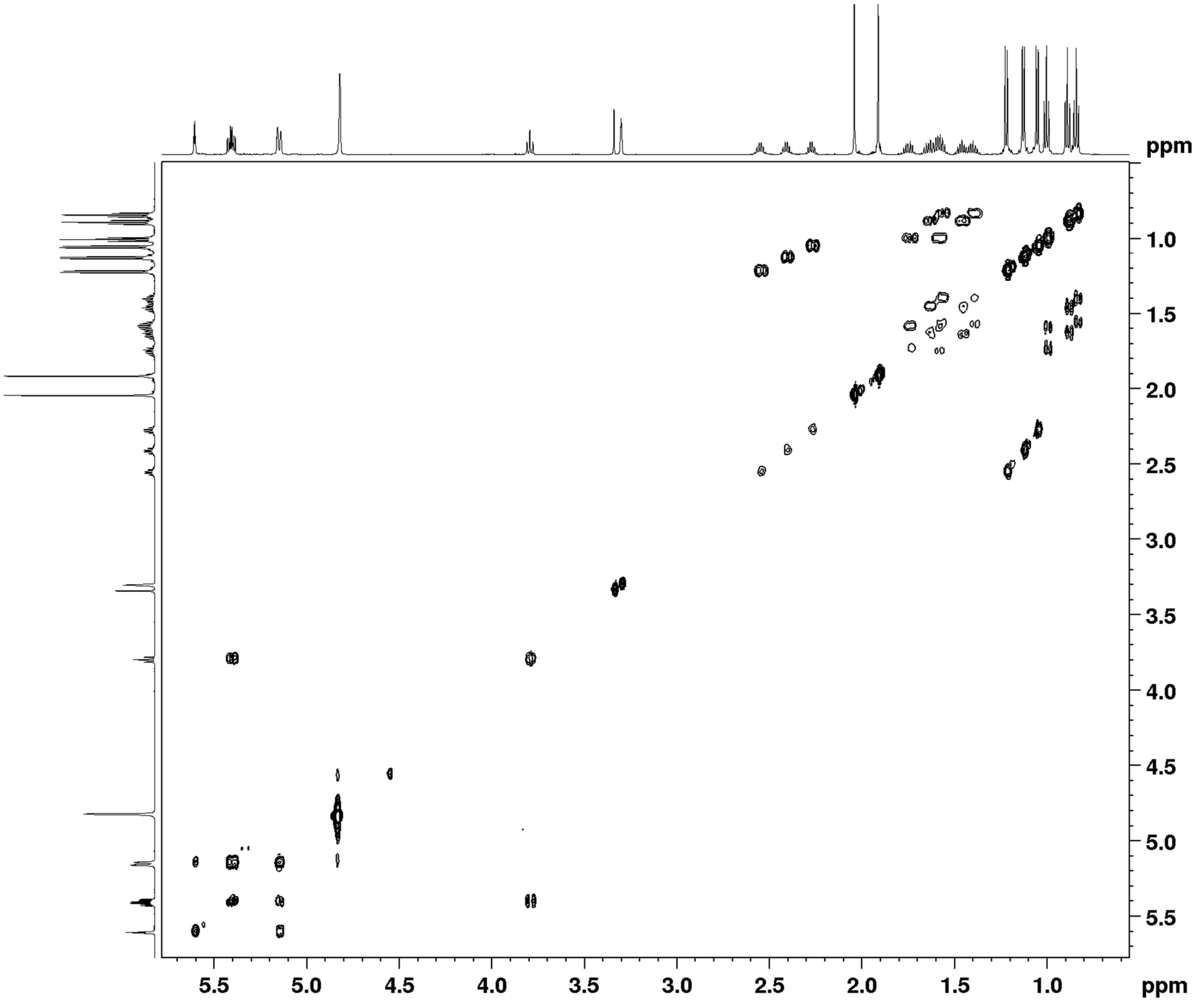

Figure S21. ^{1}H-^{1}H COSY spectrum (AV-600) of **3** in CD3OD-*d4*

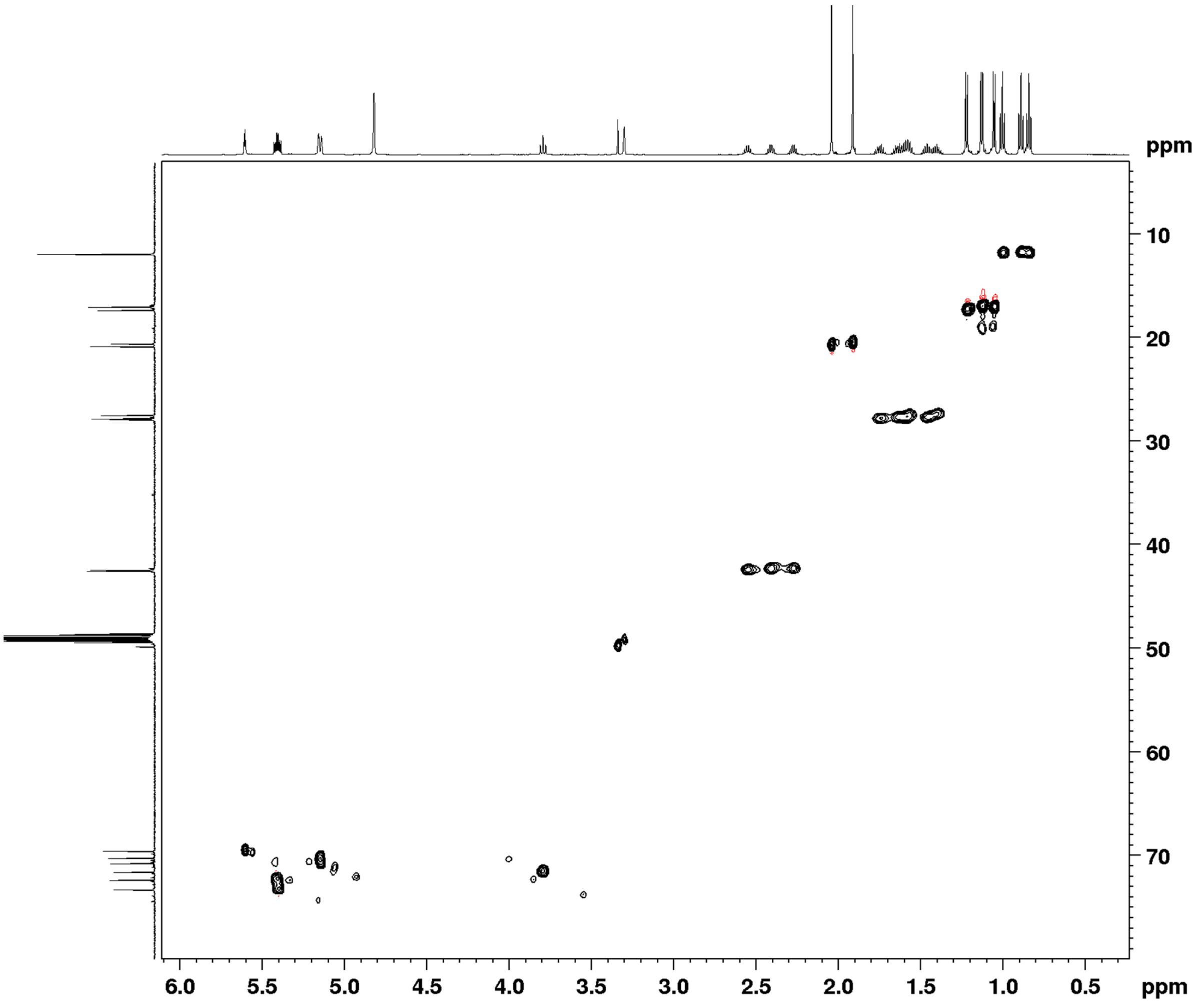

Figure S22. HSQC spectrum (AV-600) of **3** in CD_3OD-*d4*

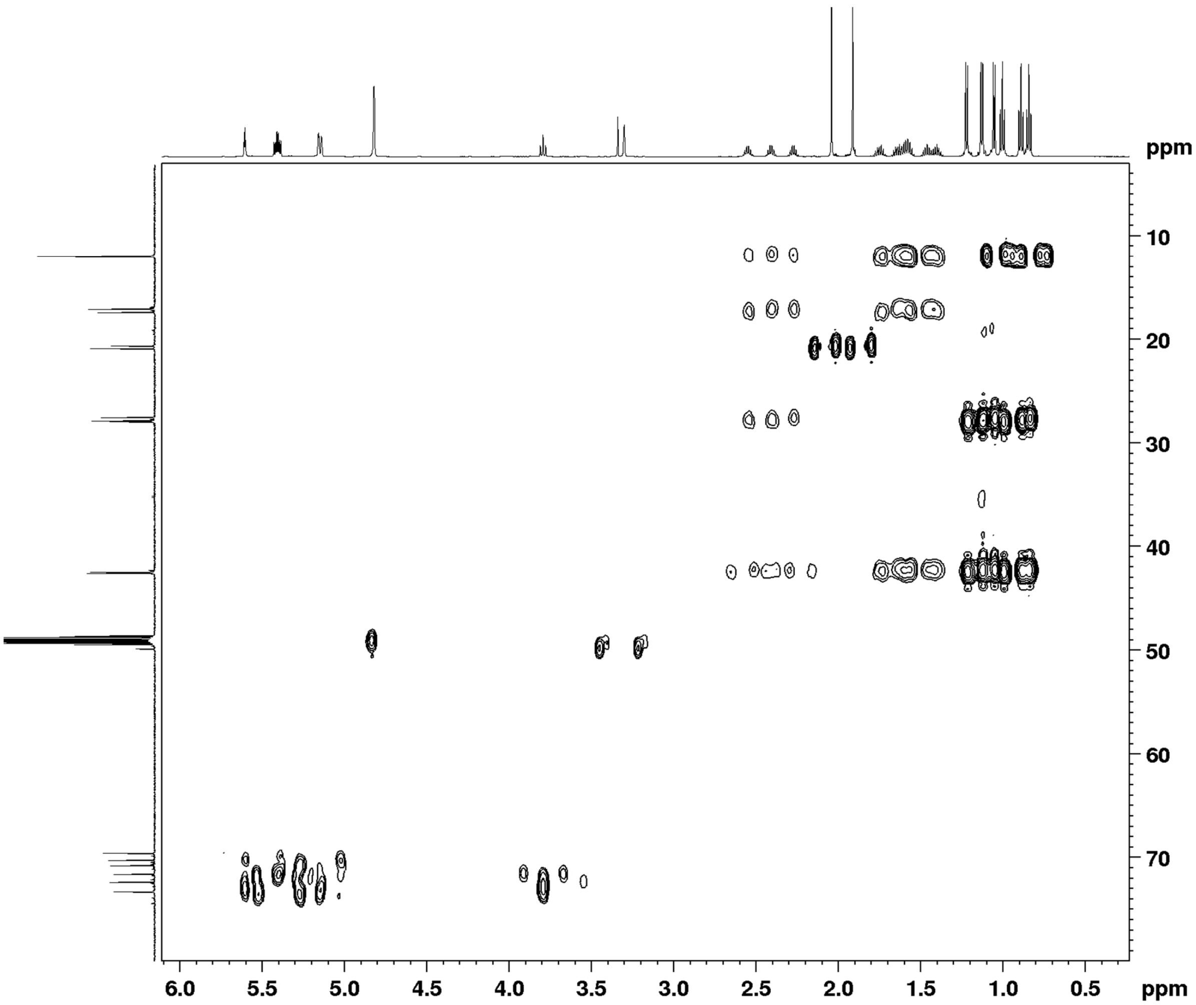

Figure S23. HMBC spectrum (AV-600) of **3** in CD3OD-*d4*

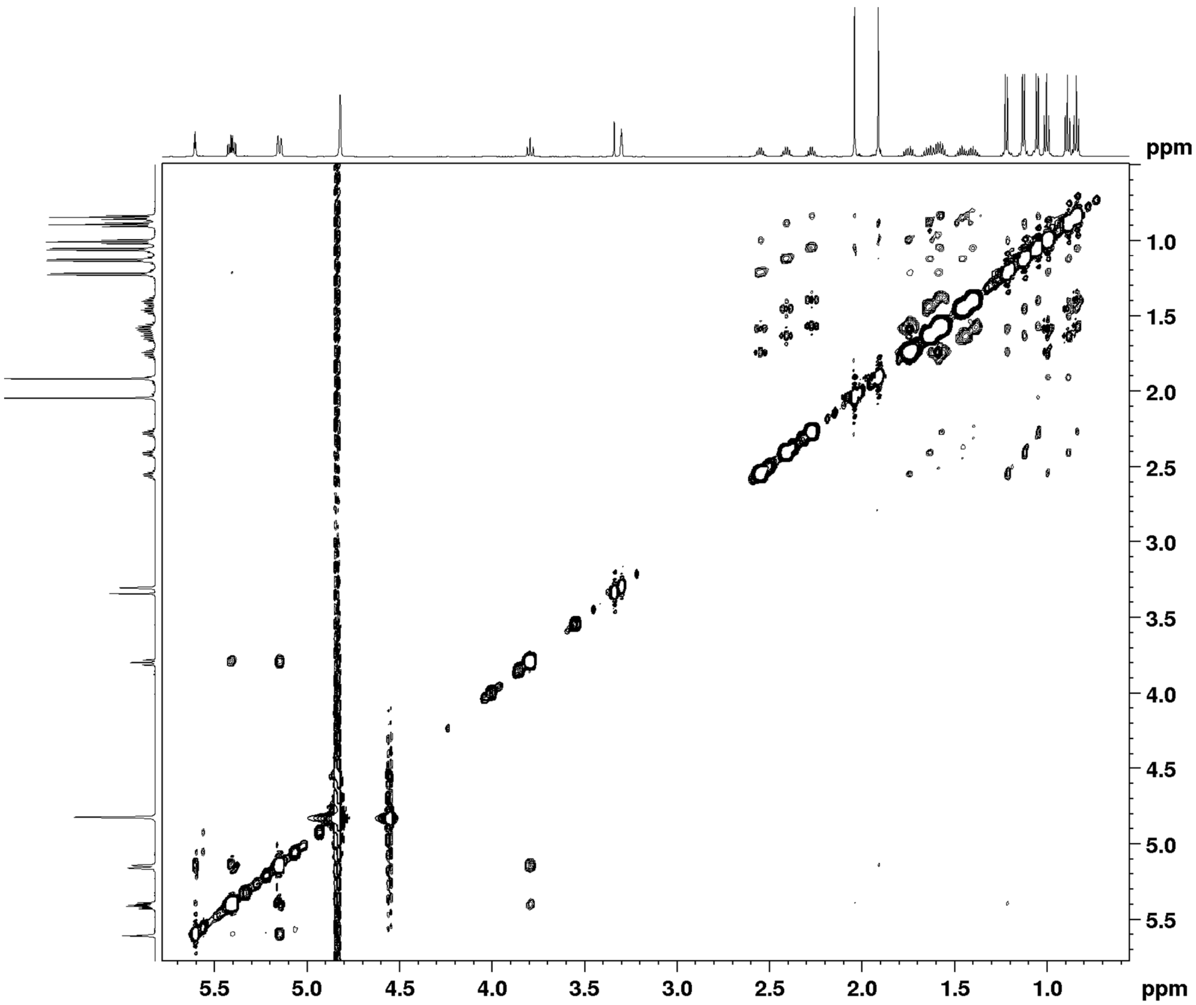

Figure S24. NOESY spectrum (AV-600) of **3** in CD_3OD-*d4*

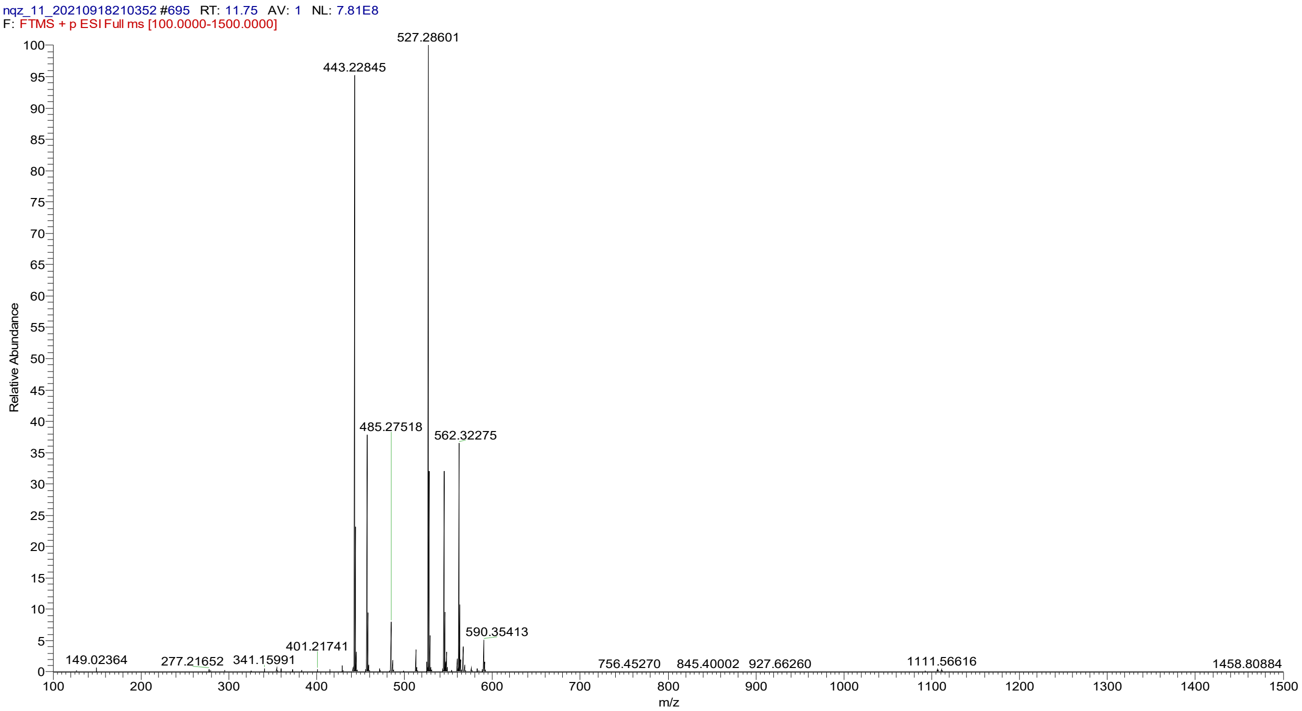

Figure S25. HR-ESI-MS spectrum of **4** in CD_3OD-*d4*

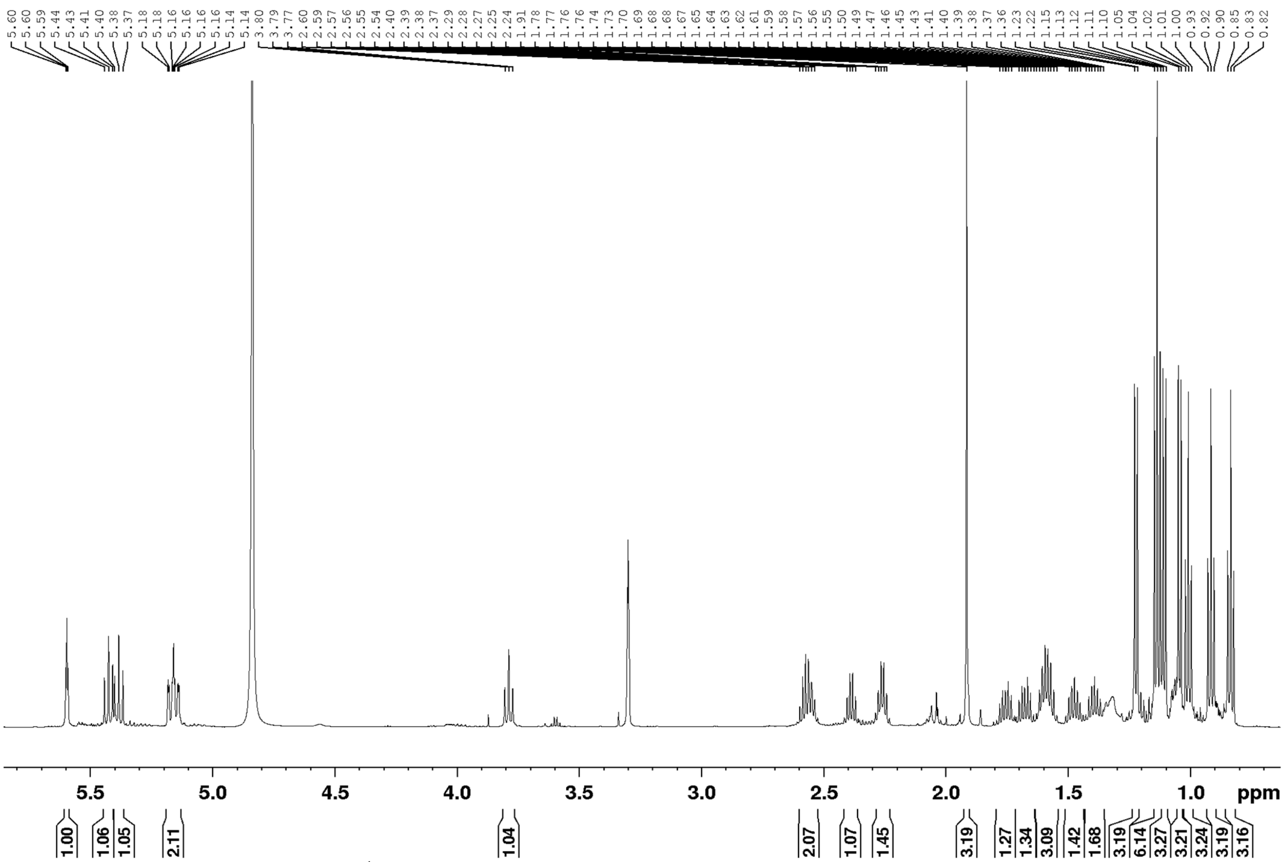

Figure S26. ^{1}H NMR spectrum (AV-600, 600 MHz) of **4** in CD_3OD-*d4*

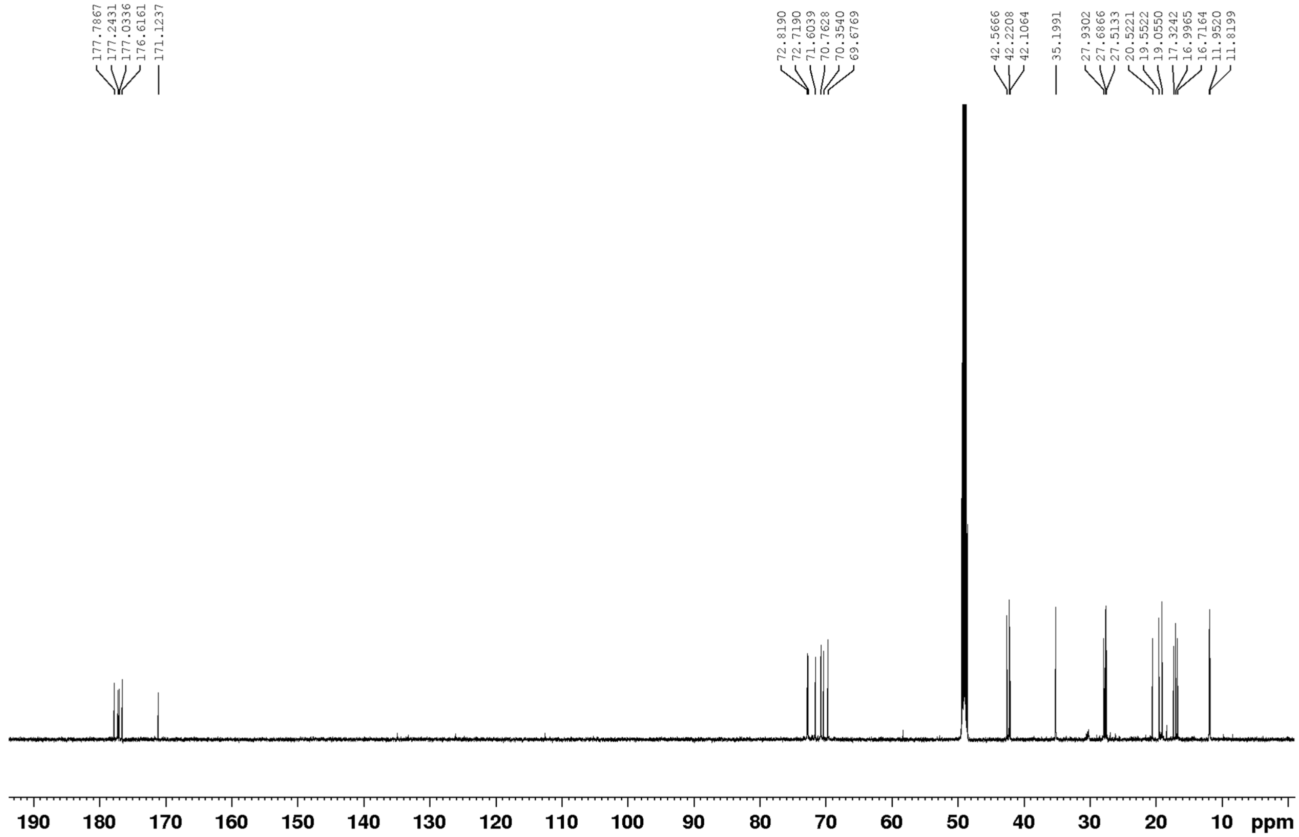

Figure S27. ^{13}C NMR spectrum (AV-600, 150 MHz) of **4** in CD3OD-*d4*

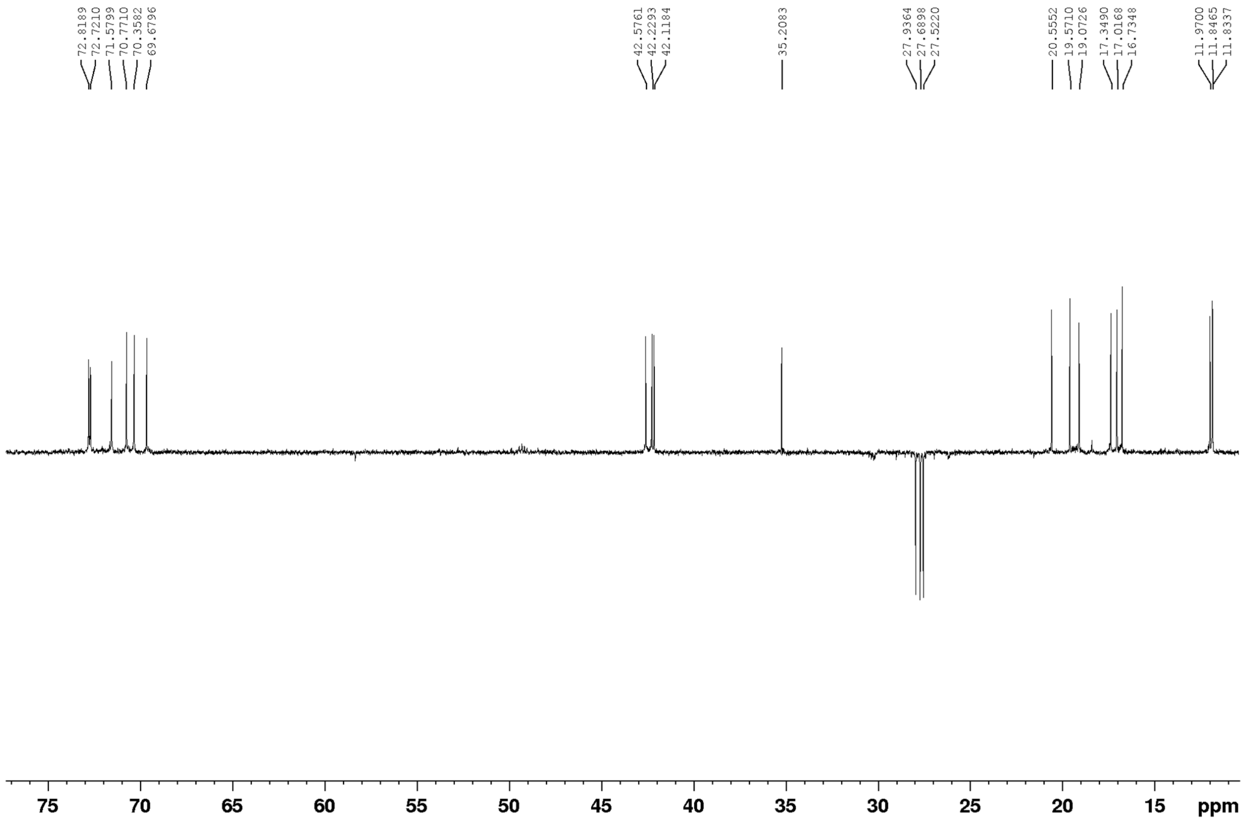

Figure S28. DEPT spectrum (AV-600) of **4** in CD3OD-*d4*

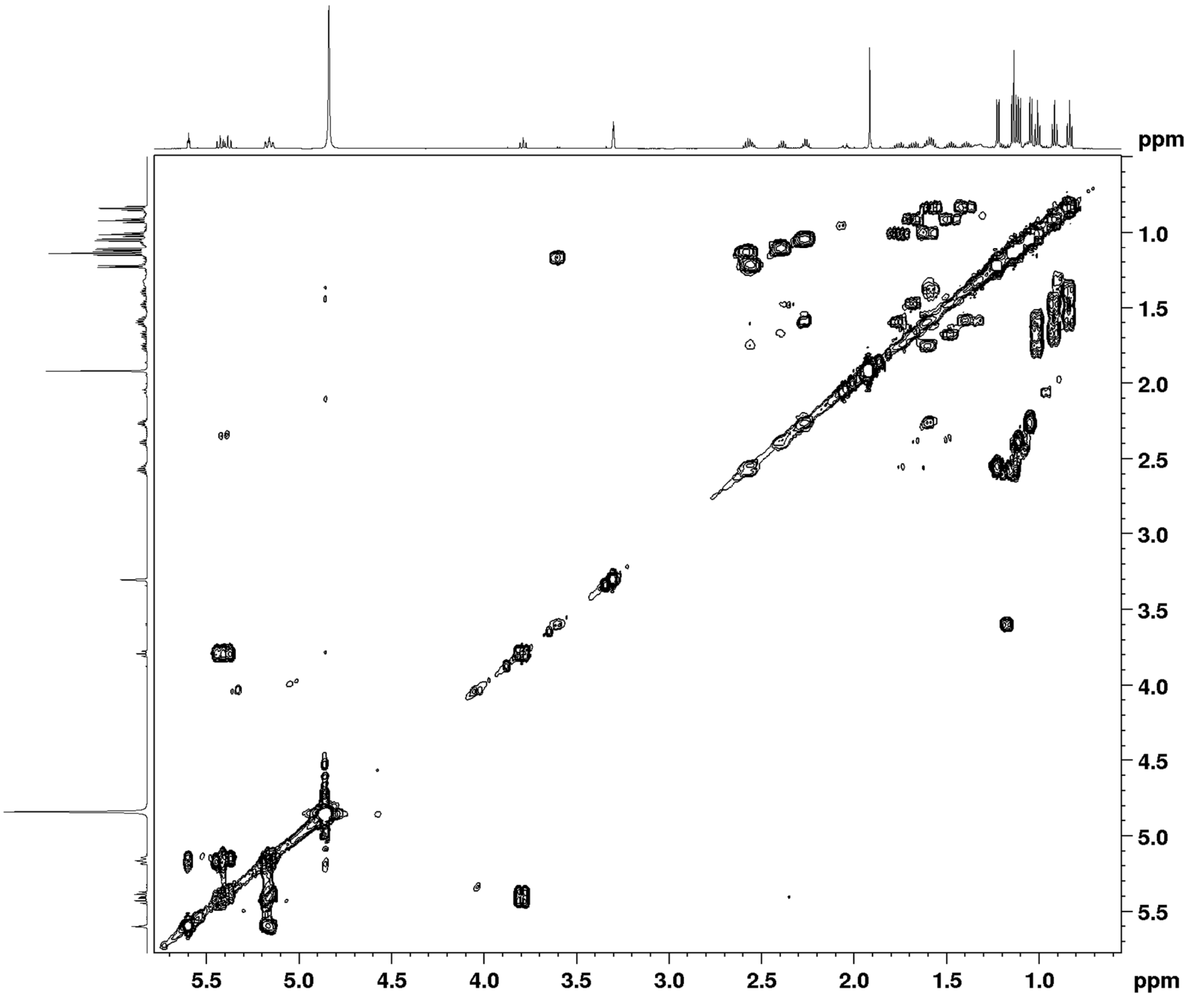

Figure S29. ^{1}H-^{1}H COSY spectrum (AV-600) of **4** in CD_3OD-*d4*

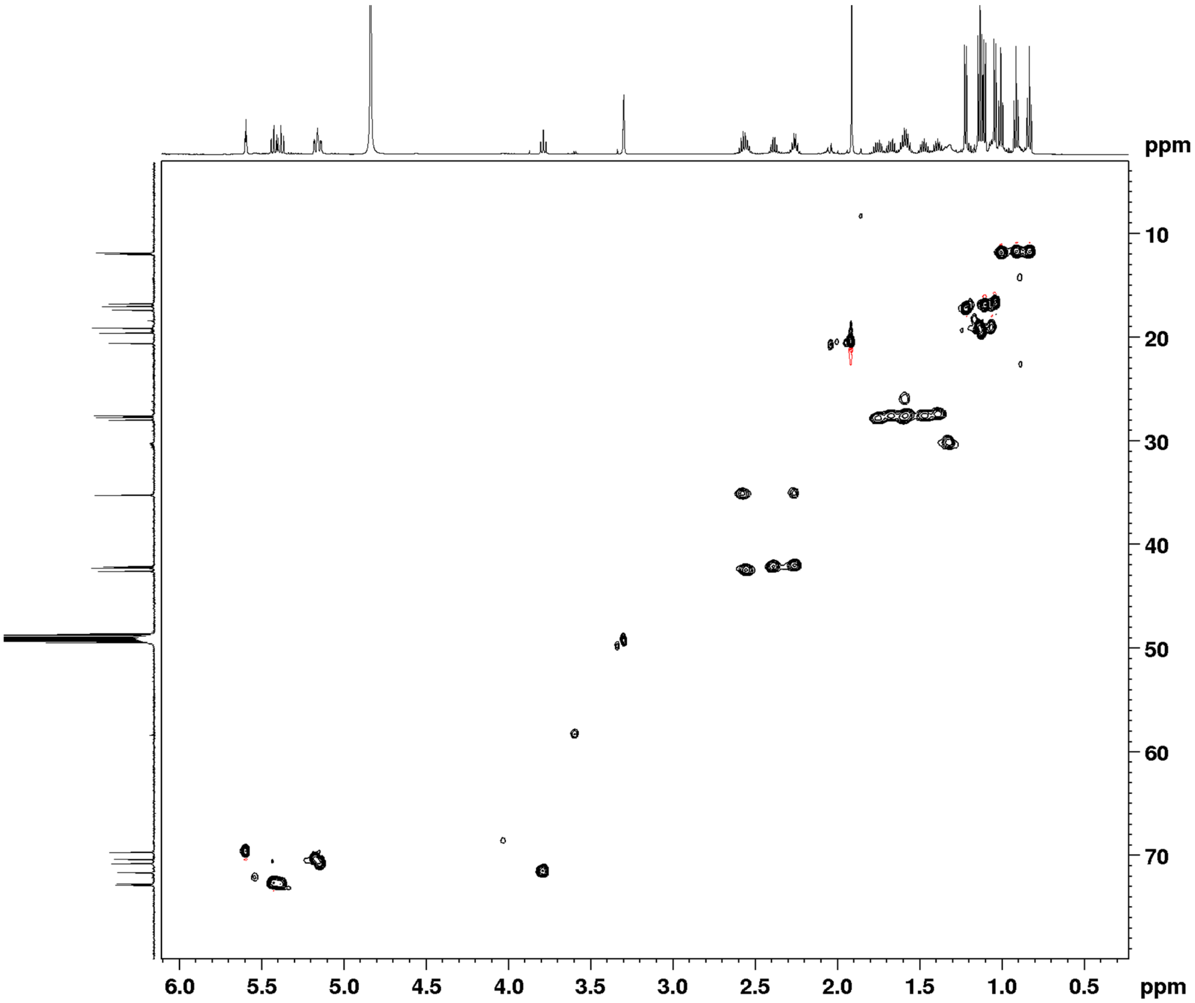

Figure S30. HSQC spectrum (AV-600) of **4** in CD_3OD-*d4*

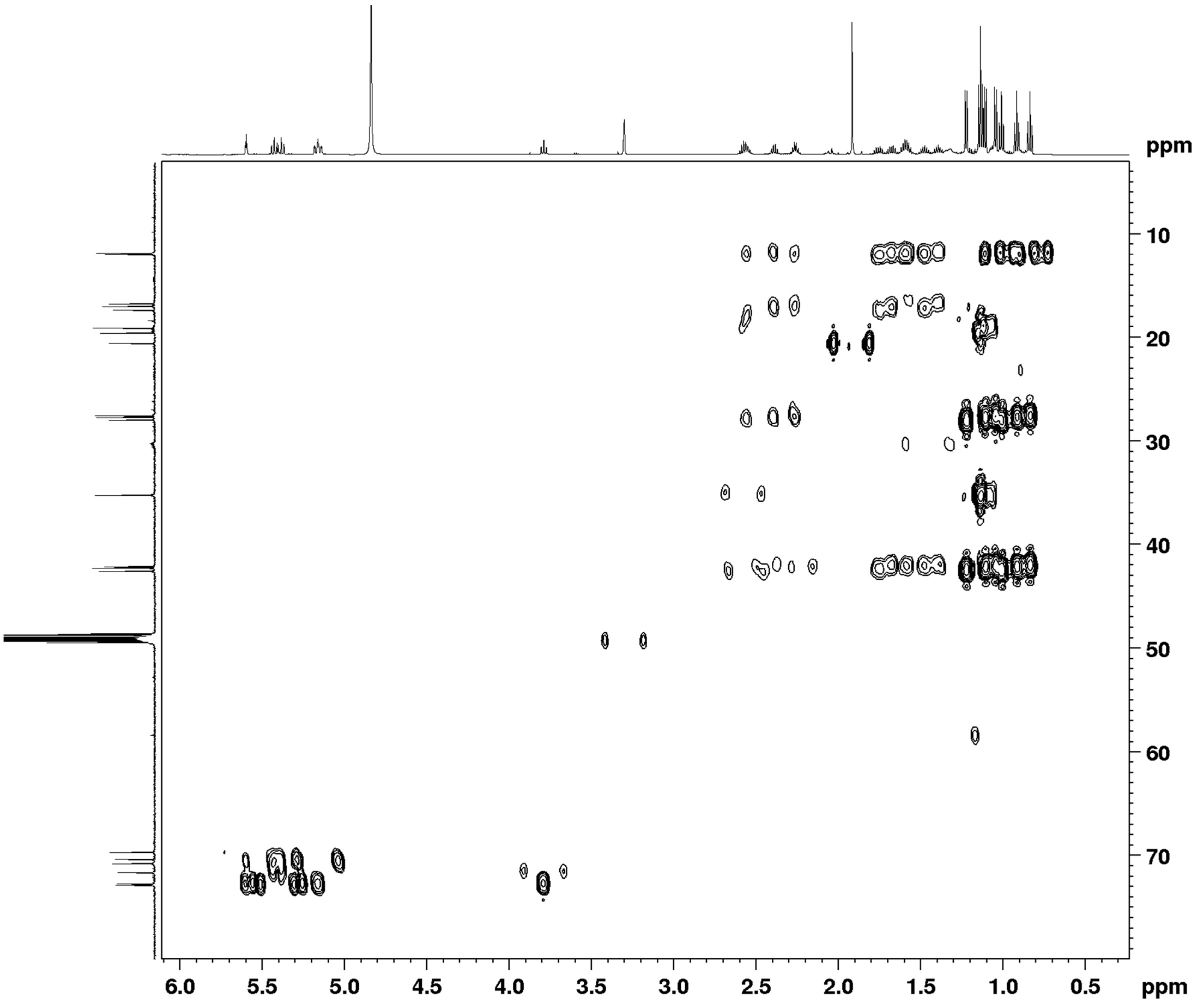

Figure S31. HMBC spectrum (AV-600) of **4** in CD_3OD-*d4*

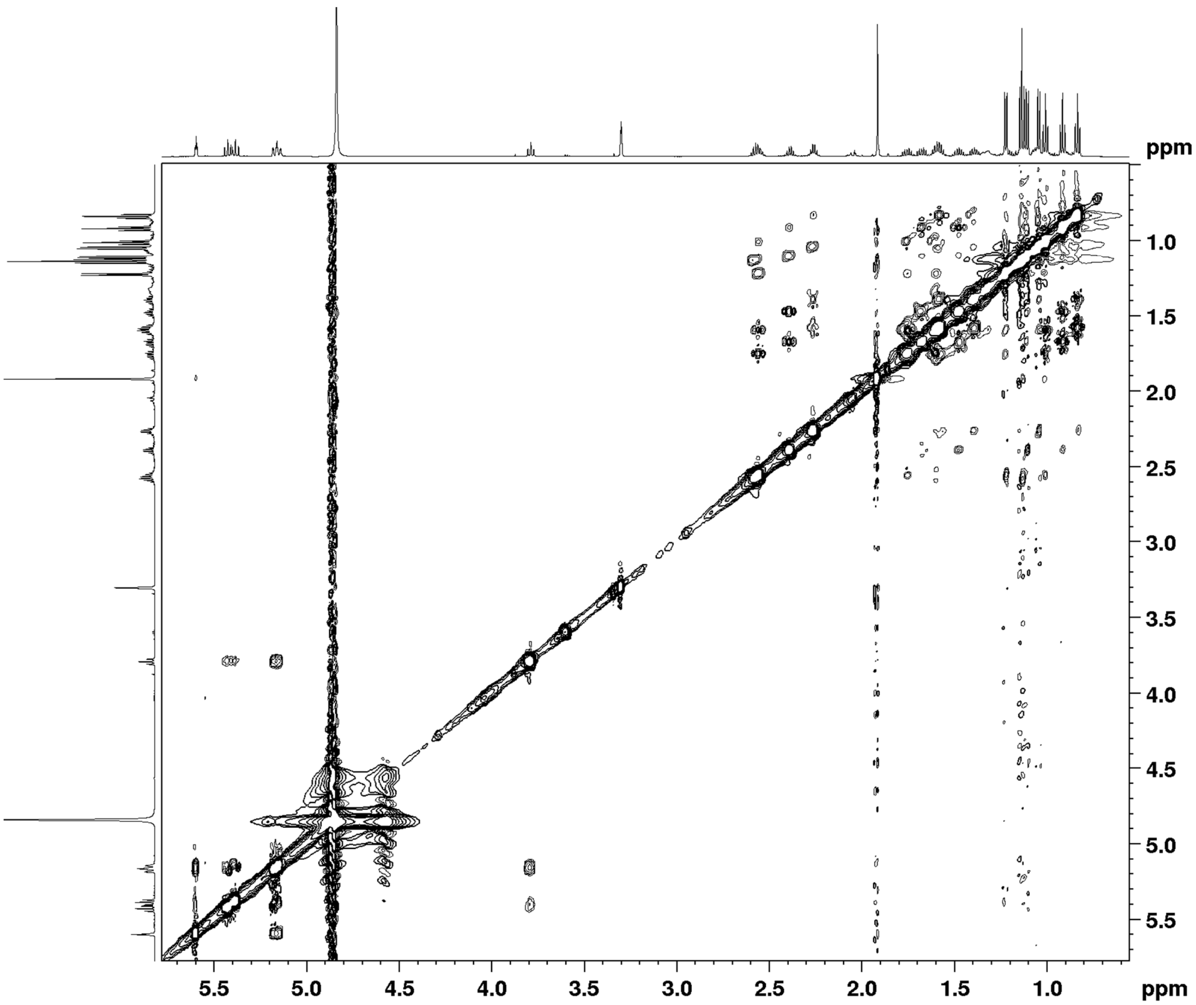

Figure S32. NOESY spectrum (AV-600) of **4** in CD3OD-*d4*

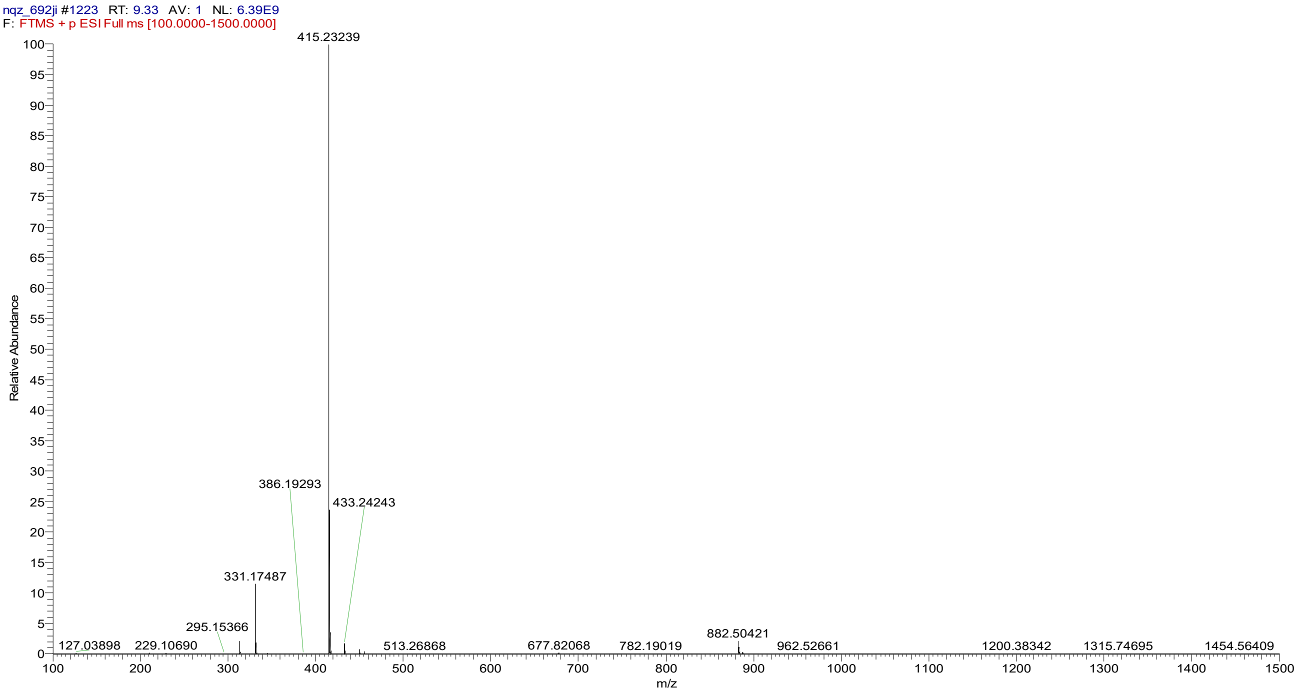

Figure S33. HR-ESI-MS spectrum of **5** in CD_3OD-*d4*

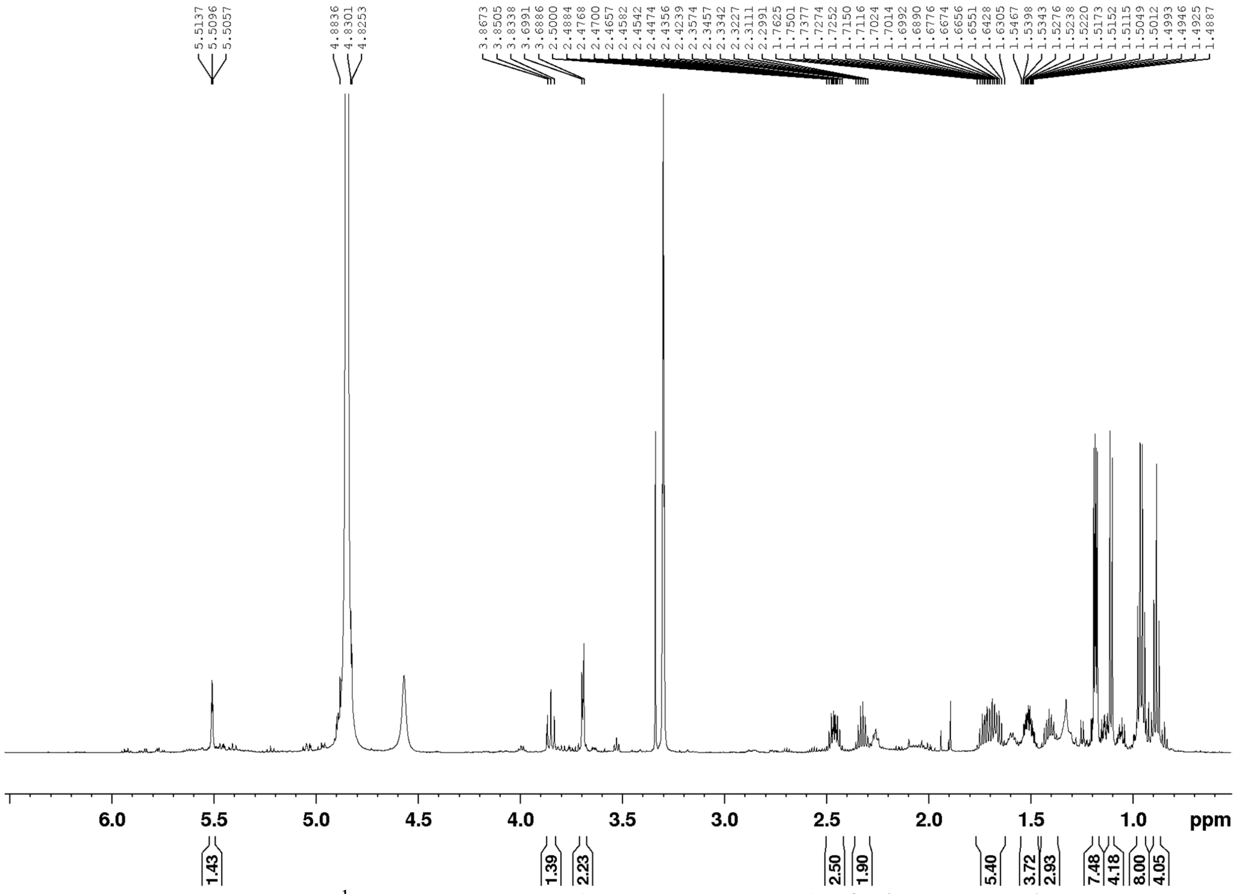

Figure S34. ^{1}H NMR spectrum (AV-600, 600 MHz) of **5** in CD_3OD-*d4*

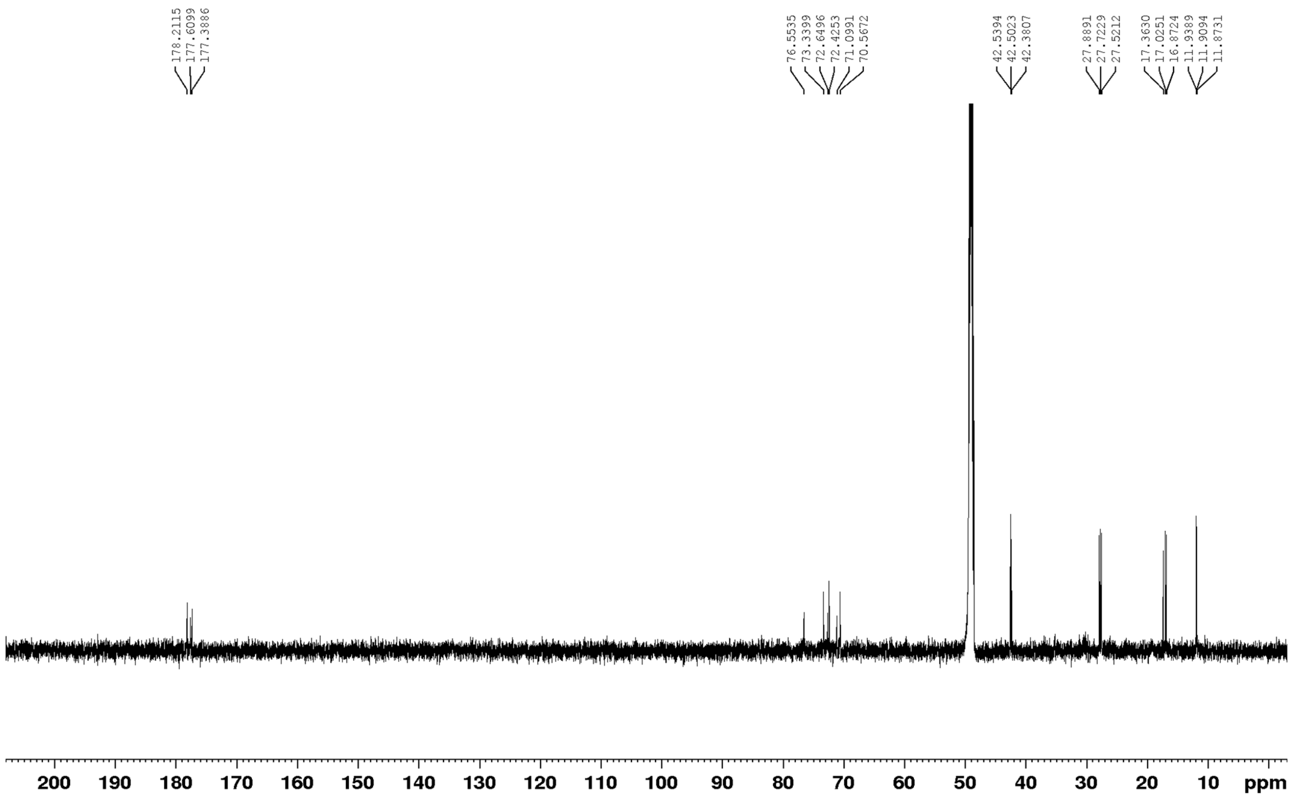

Figure S35.^{13}C NMR spectrum (AV-600, 150 MHz) of **5** in CD_3OD-*d4*

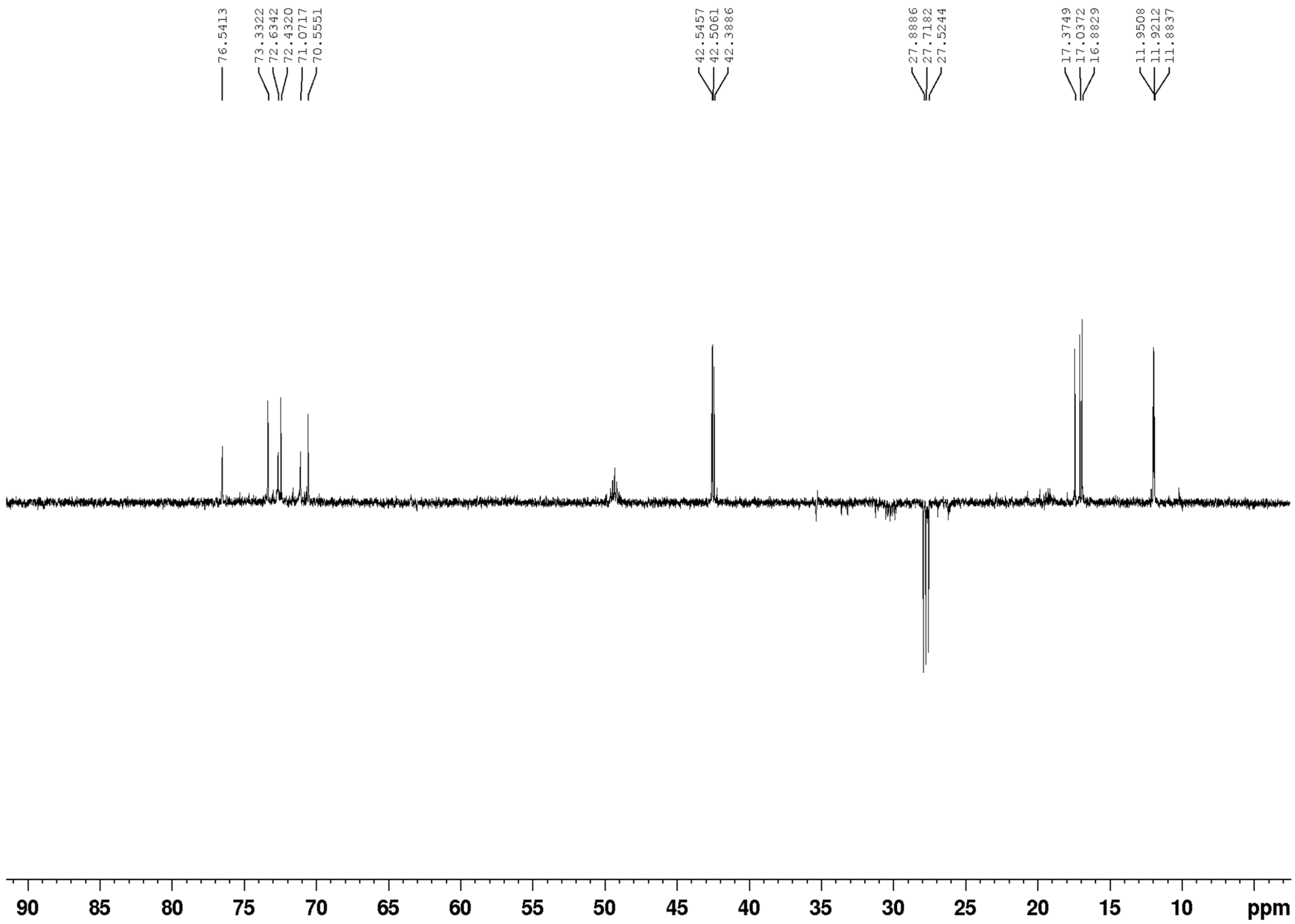

Figure S36. DEPT spectrum (AV-600) of **5** in CD_3OD-*d4*

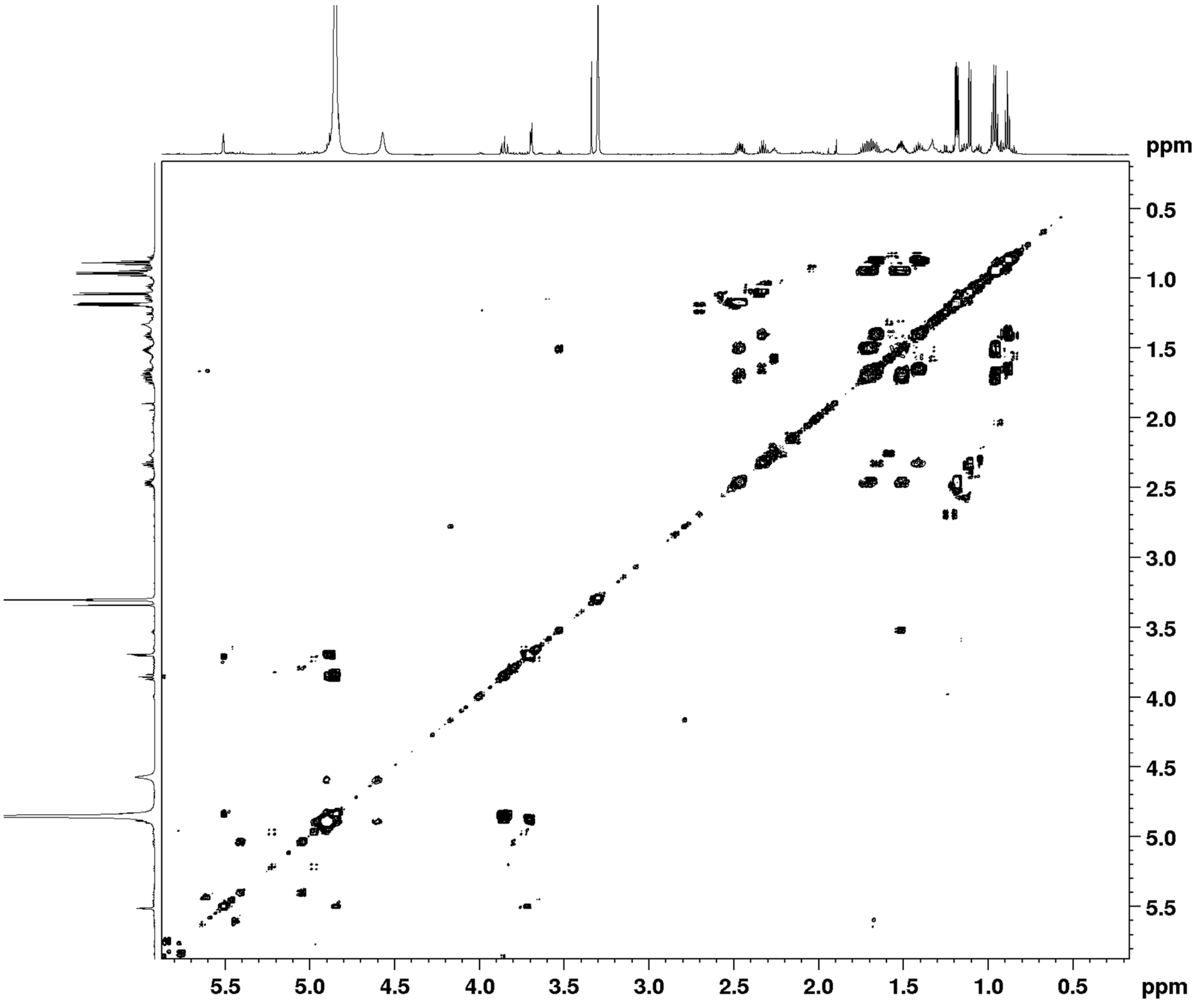

Figure S37. ^{1}H-^{1}H COSY spectrum (AV-600) of **5** in CD_3OD-*d4*

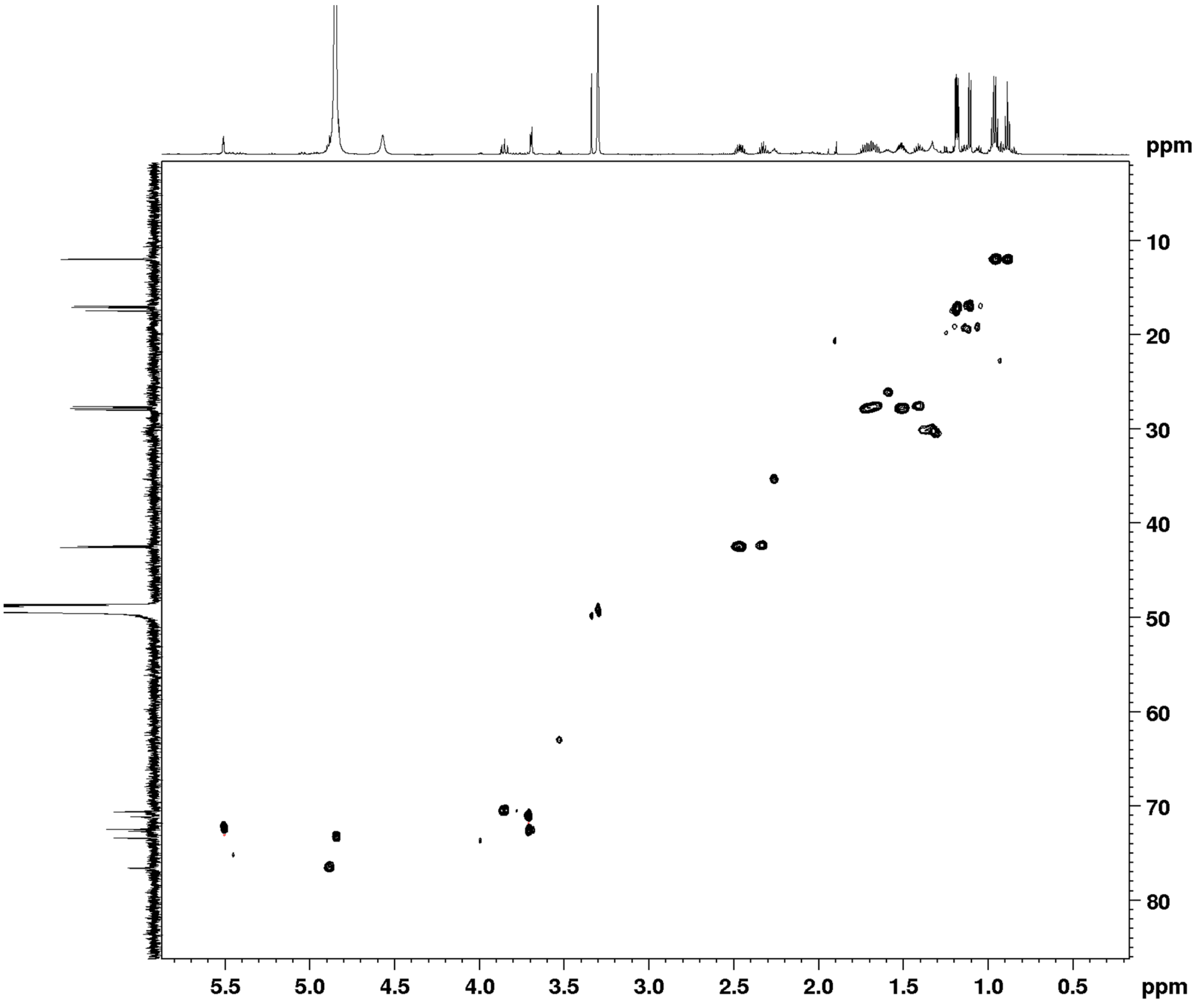

Figure S38. HSQC spectrum (AV-600) of **5** in CD_3OD-*d4*

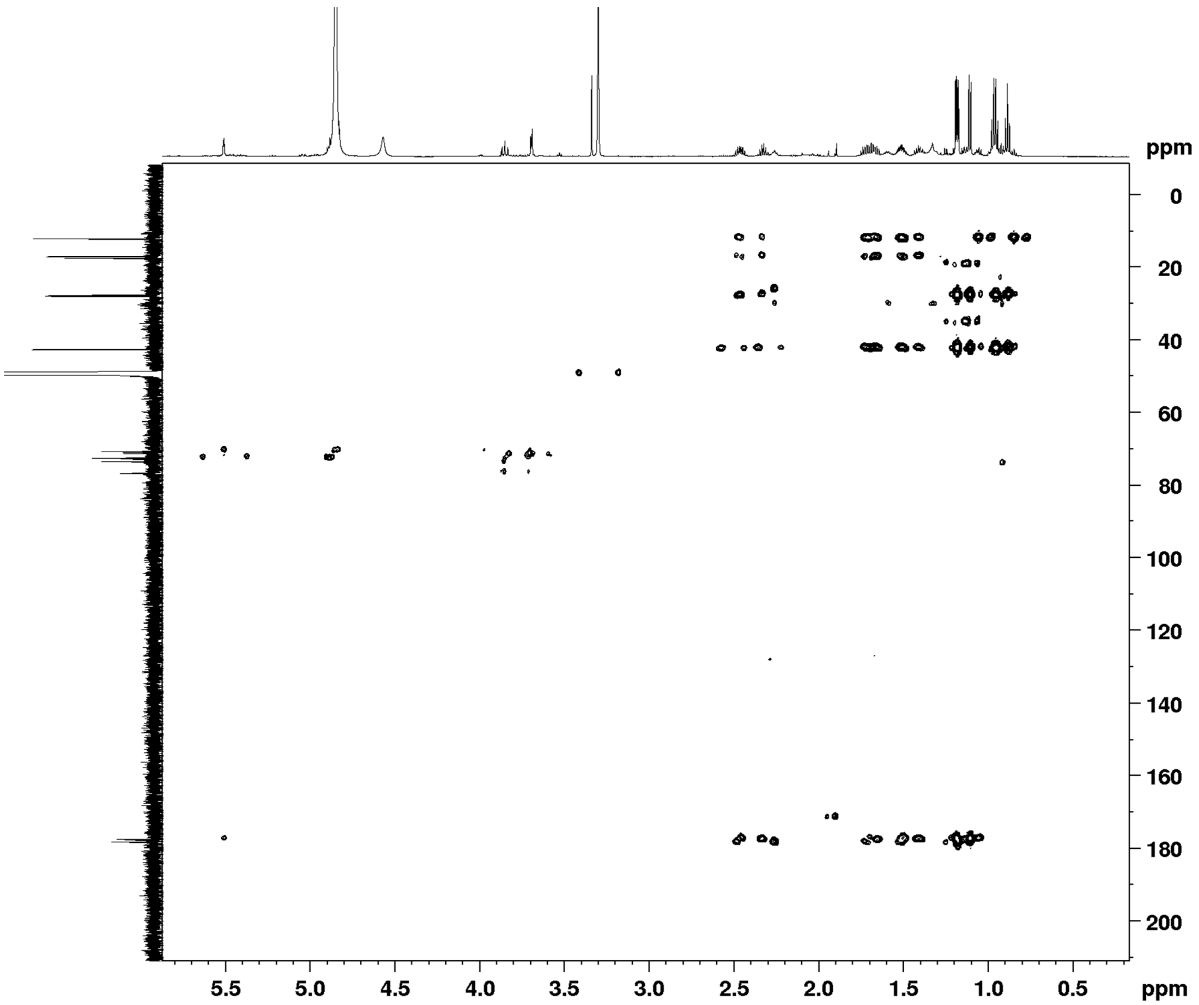

Figure S39. HMBC spectrum (AV-600) of **5** in CD3OD-*d4*

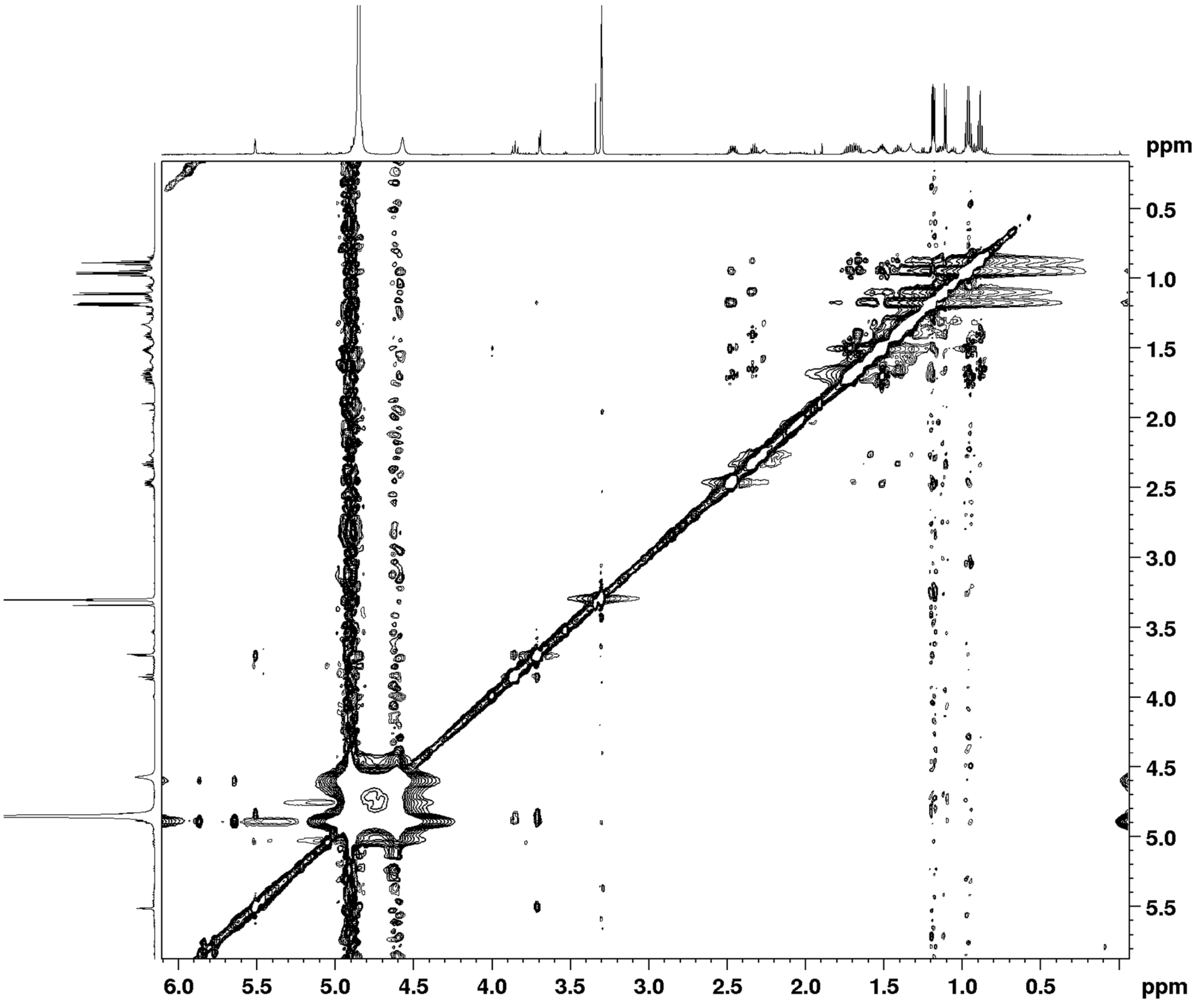

Figure S40. NOESY spectrum (AV-600) of **5** in CD3OD-*d4*

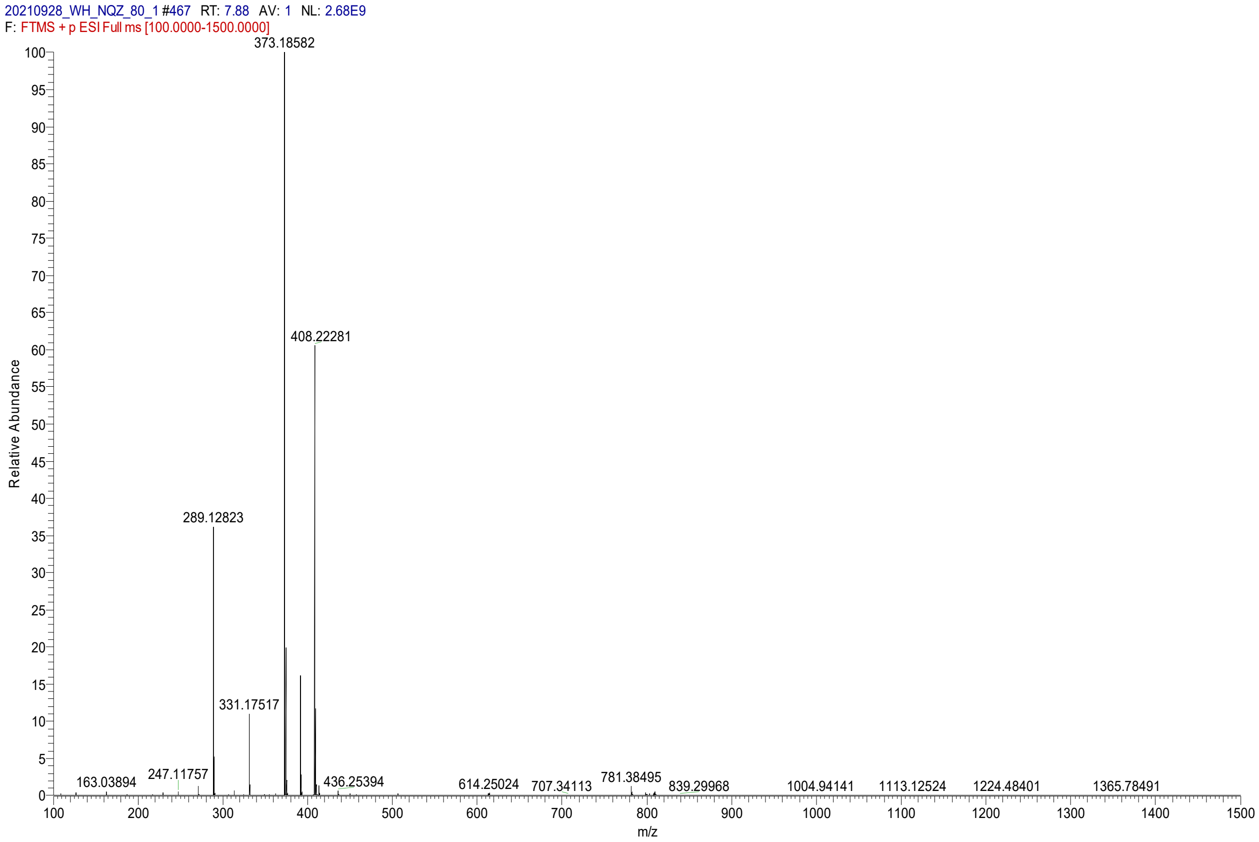

Figure S41. HR-ESI-MS spectrum of **6** in CD_3OD-*d4*

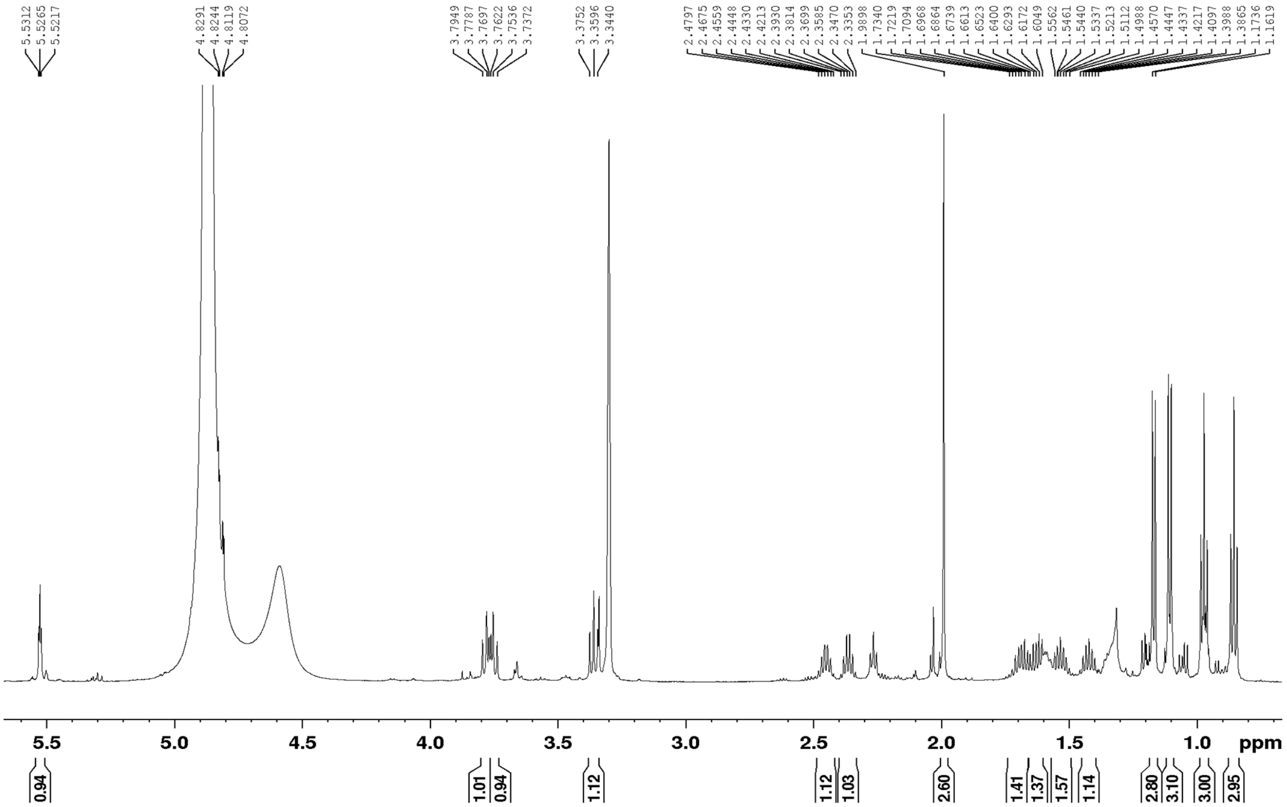

Figure S42. ^{1}H NMR spectrum (AV-600, 600 MHz) of **6** in CD_3OD-*d4*

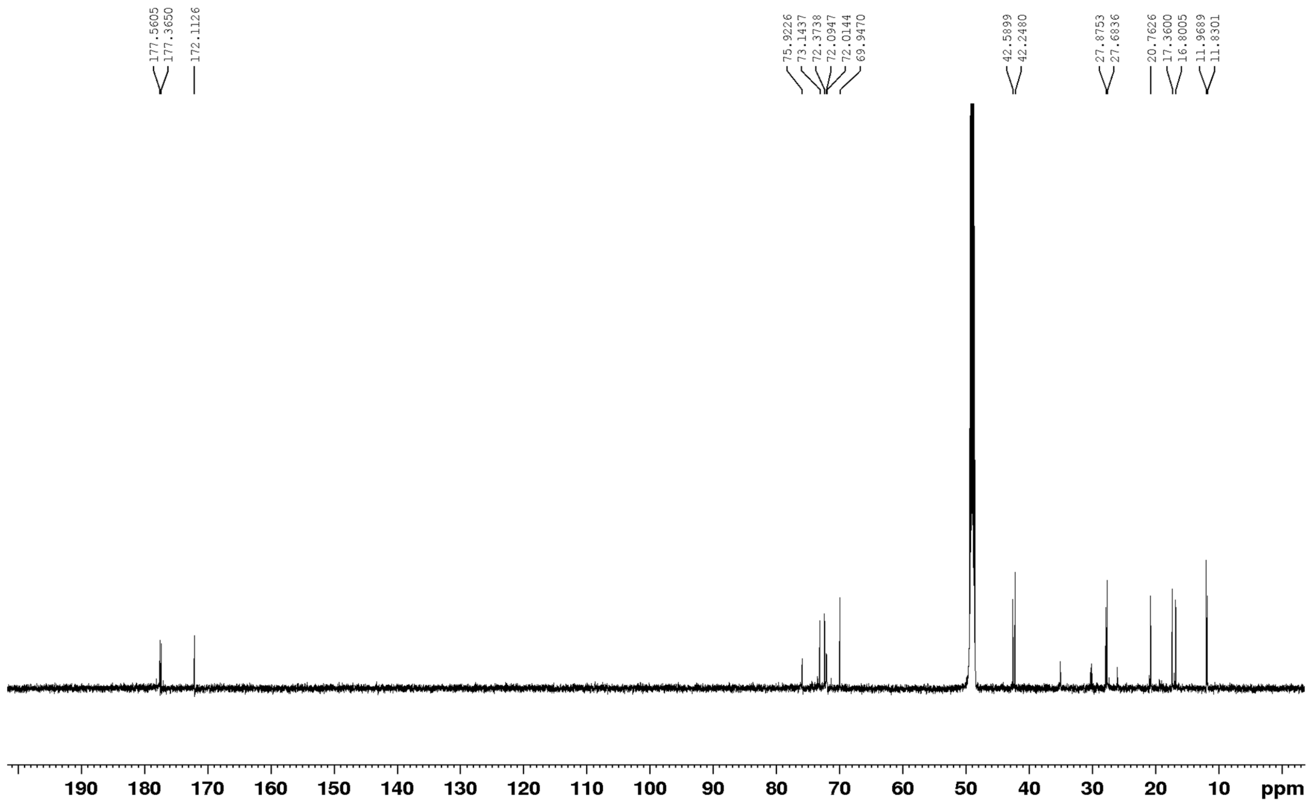

Figure S43. ^{13}C NMR spectrum (AV-600, 150 MHz) of **6** in CD3OD-*d4*

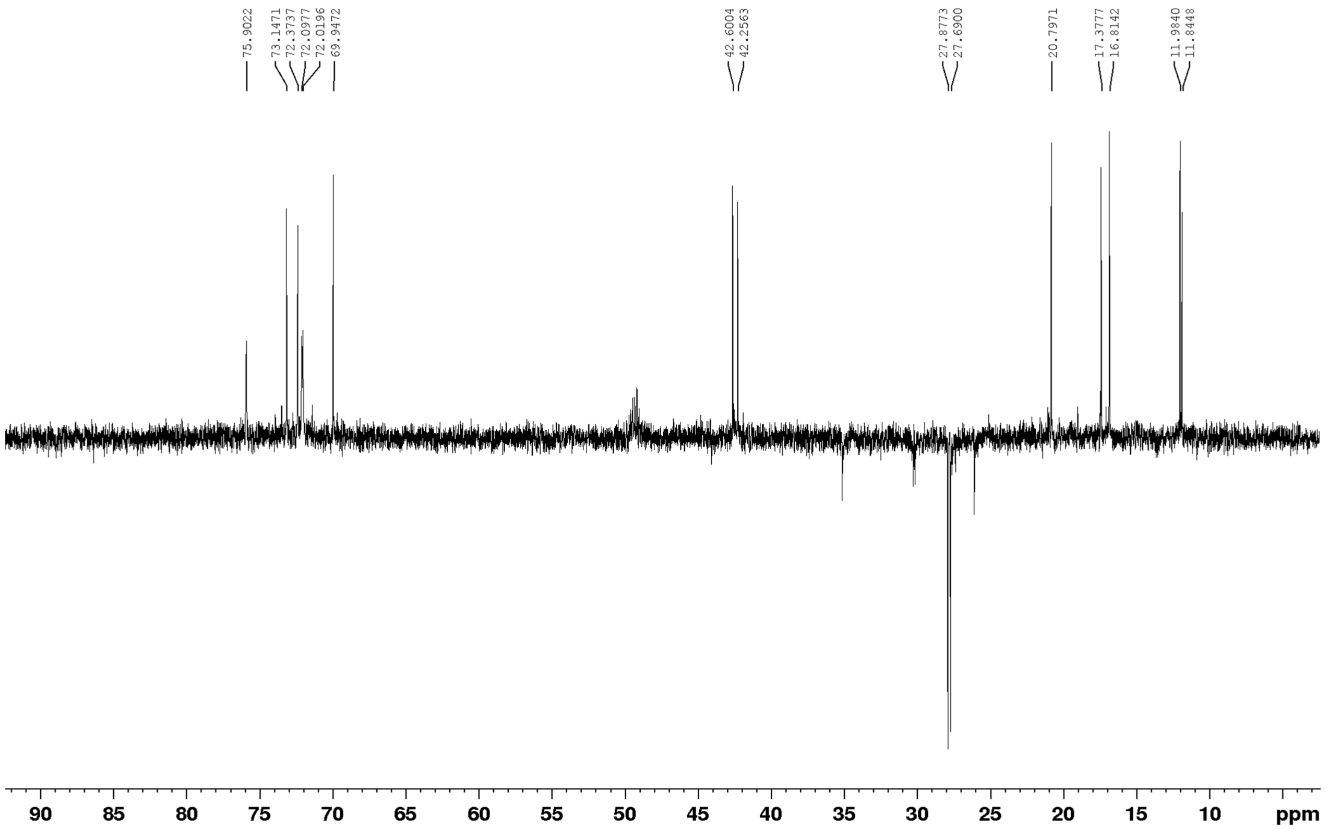

Figure S44. DEPT spectrum (AV-600) of **6** in CD_3OD-*d4*

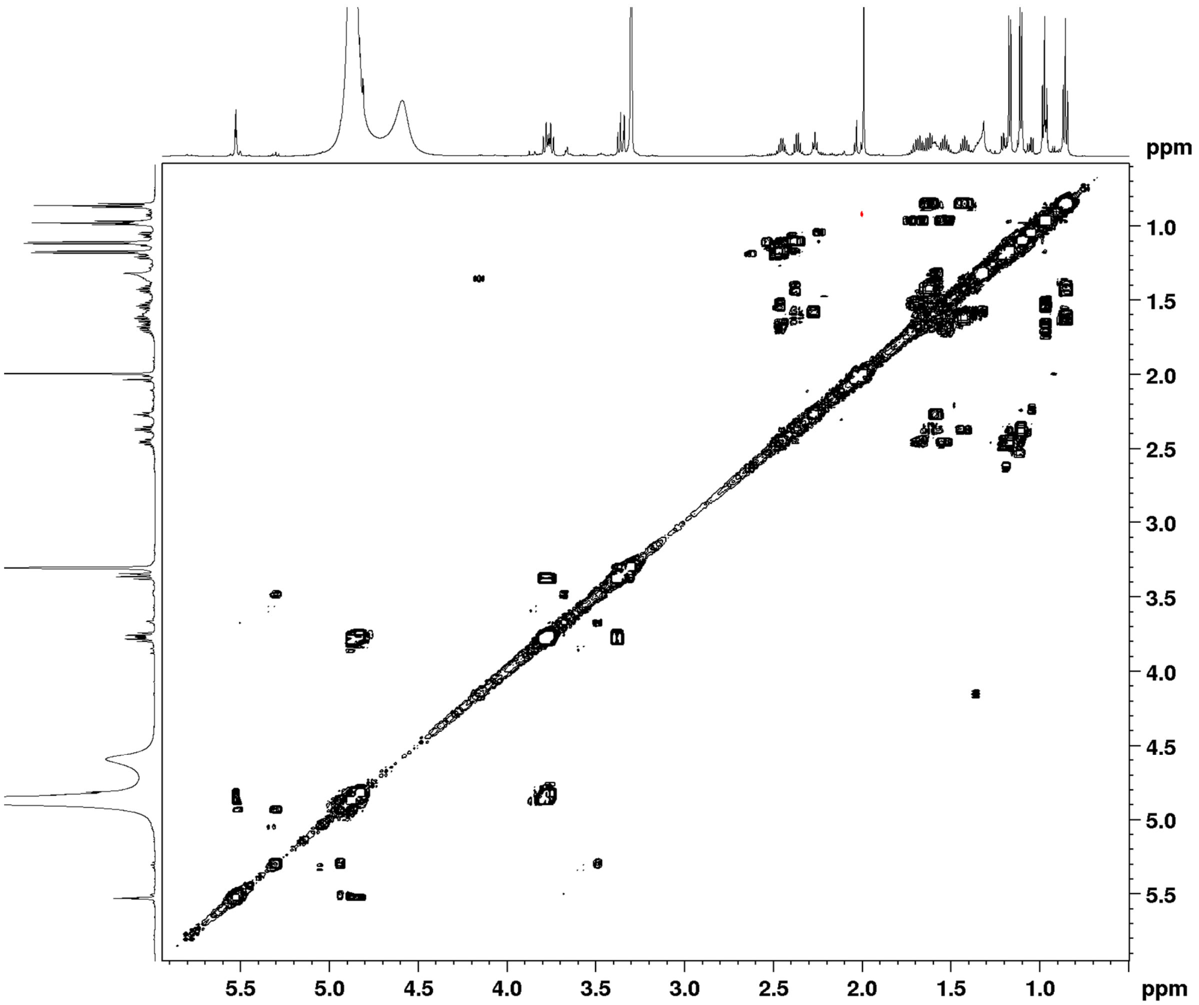

Figure S45. ^1H-^1H COSY spectrum (AV-600) of **6** in CD_3OD-*d4*

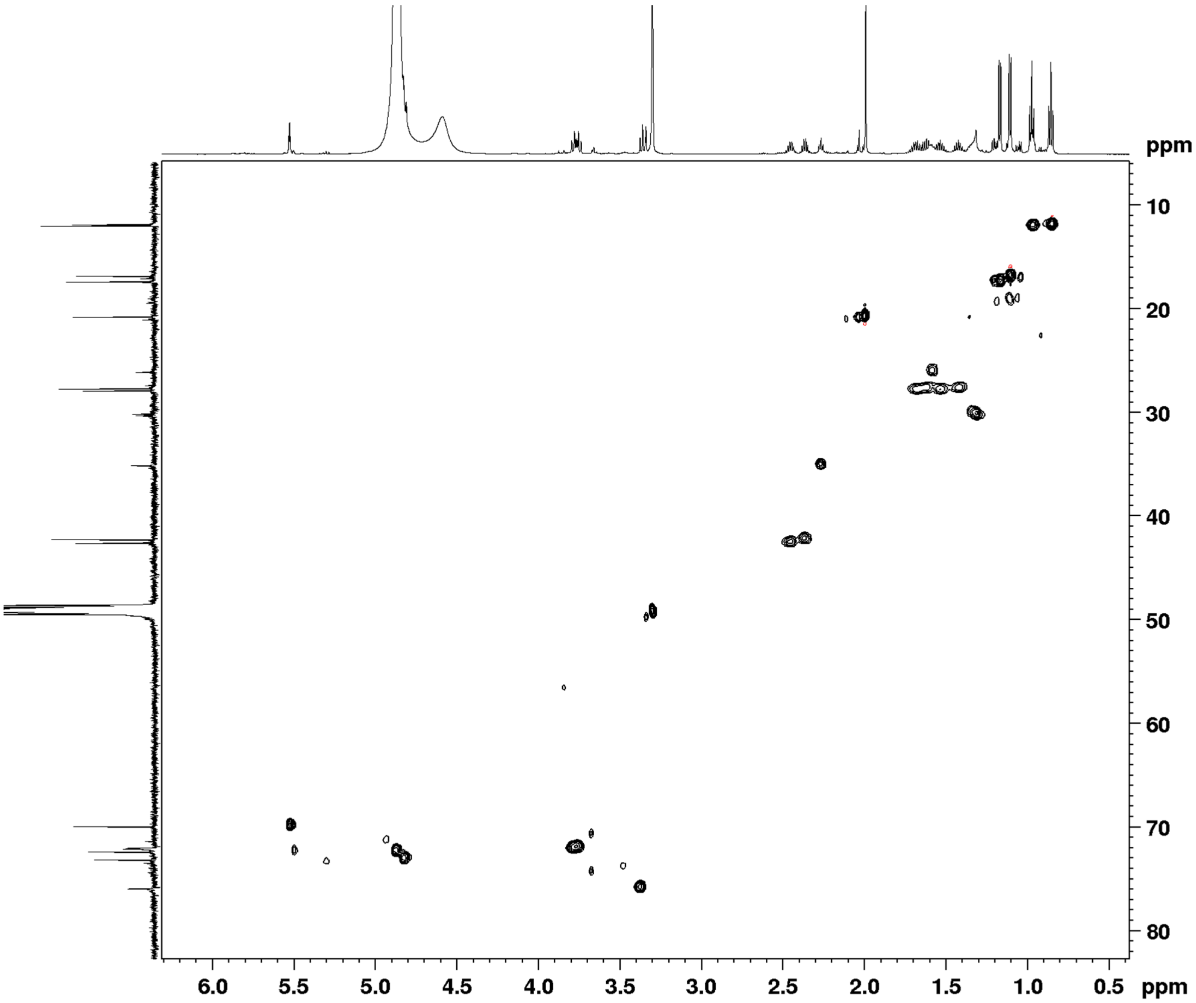

Figure S46. HSQC spectrum (AV-600) of **6** in CD3OD-*d4*

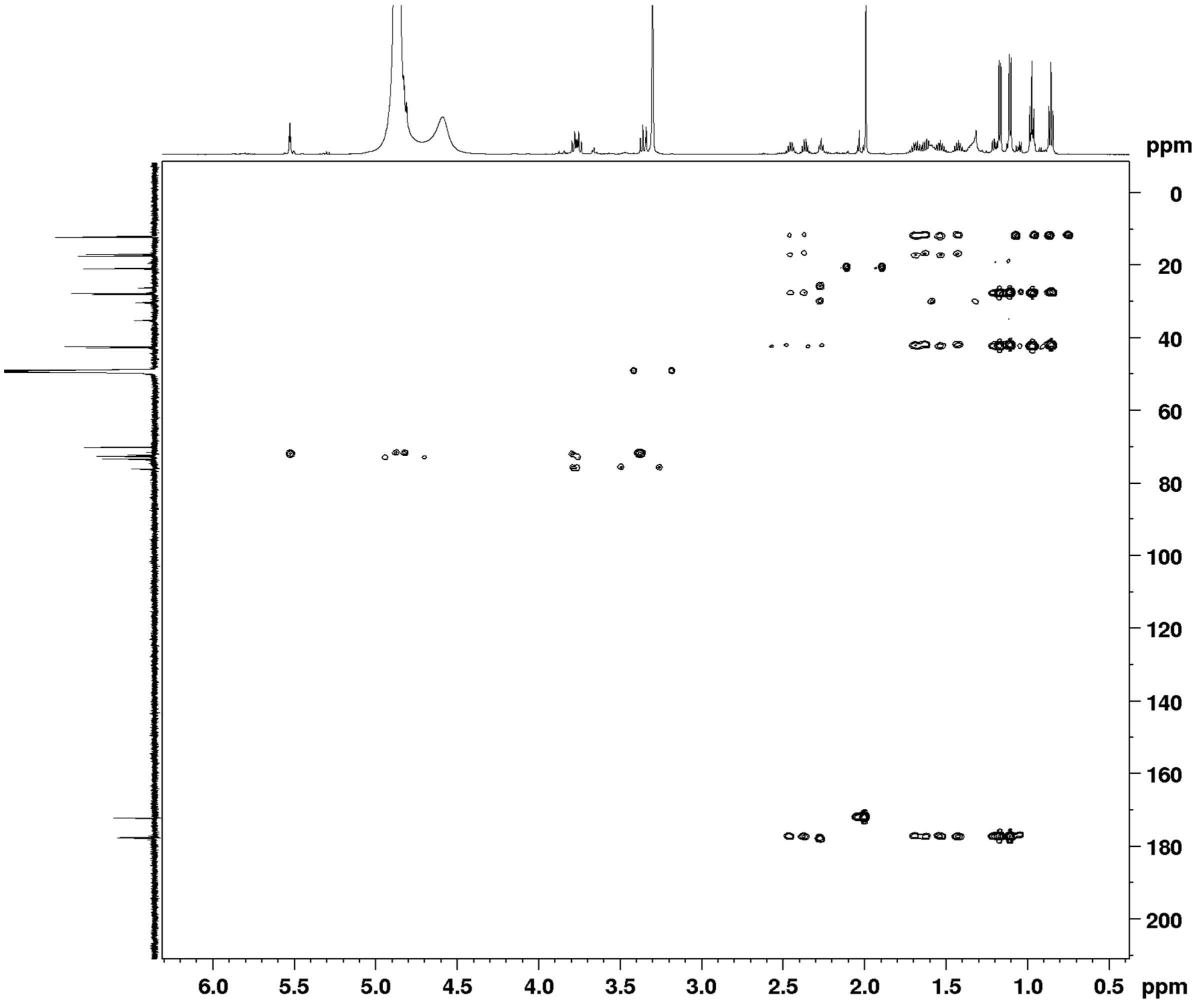

Figure S47. HMBC spectrum (AV-600) of **6** in CD_3OD-*d4*

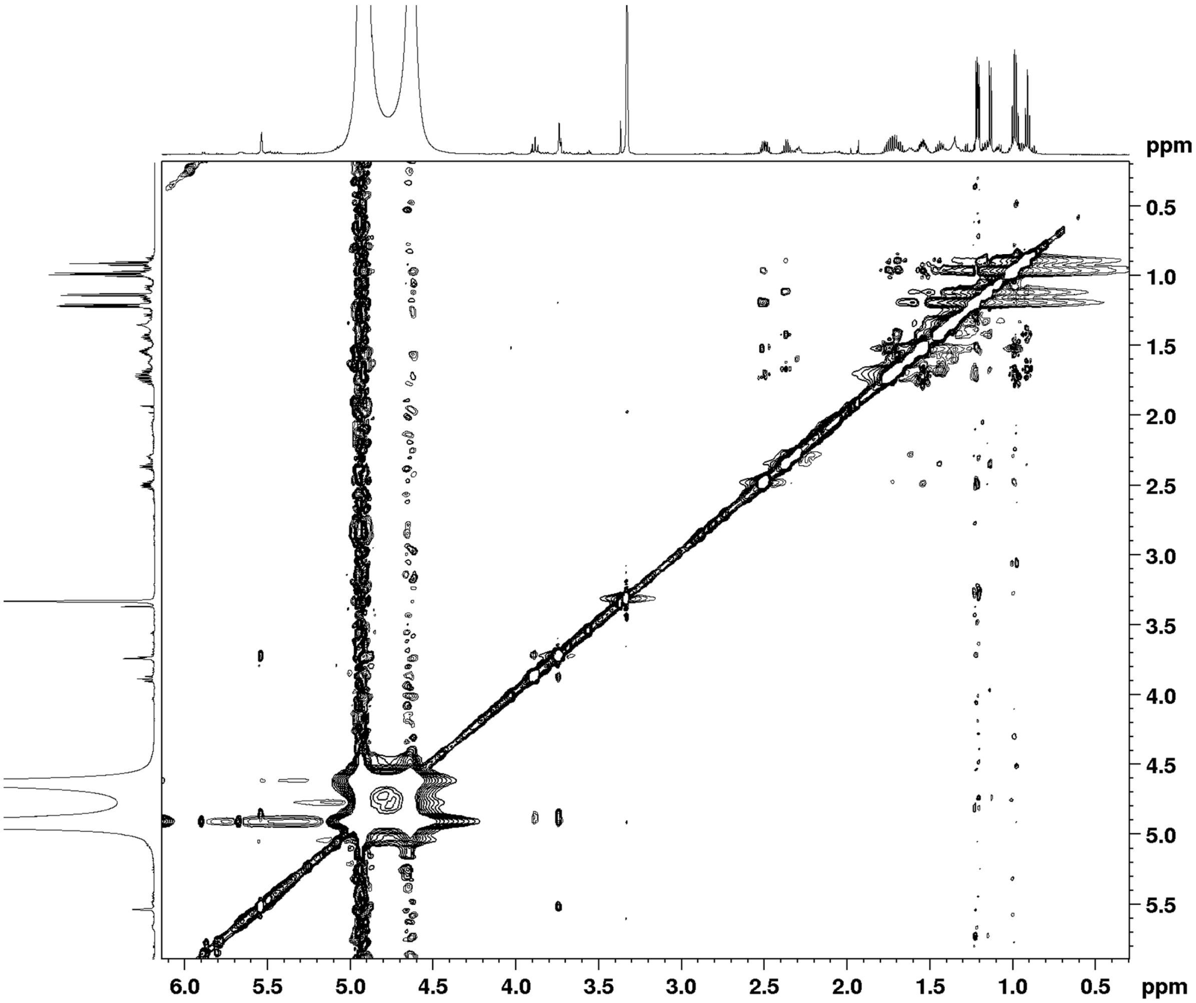

Figure S48. NOESY spectrum (AV-600) of **6** in CD_3OD-*d4*

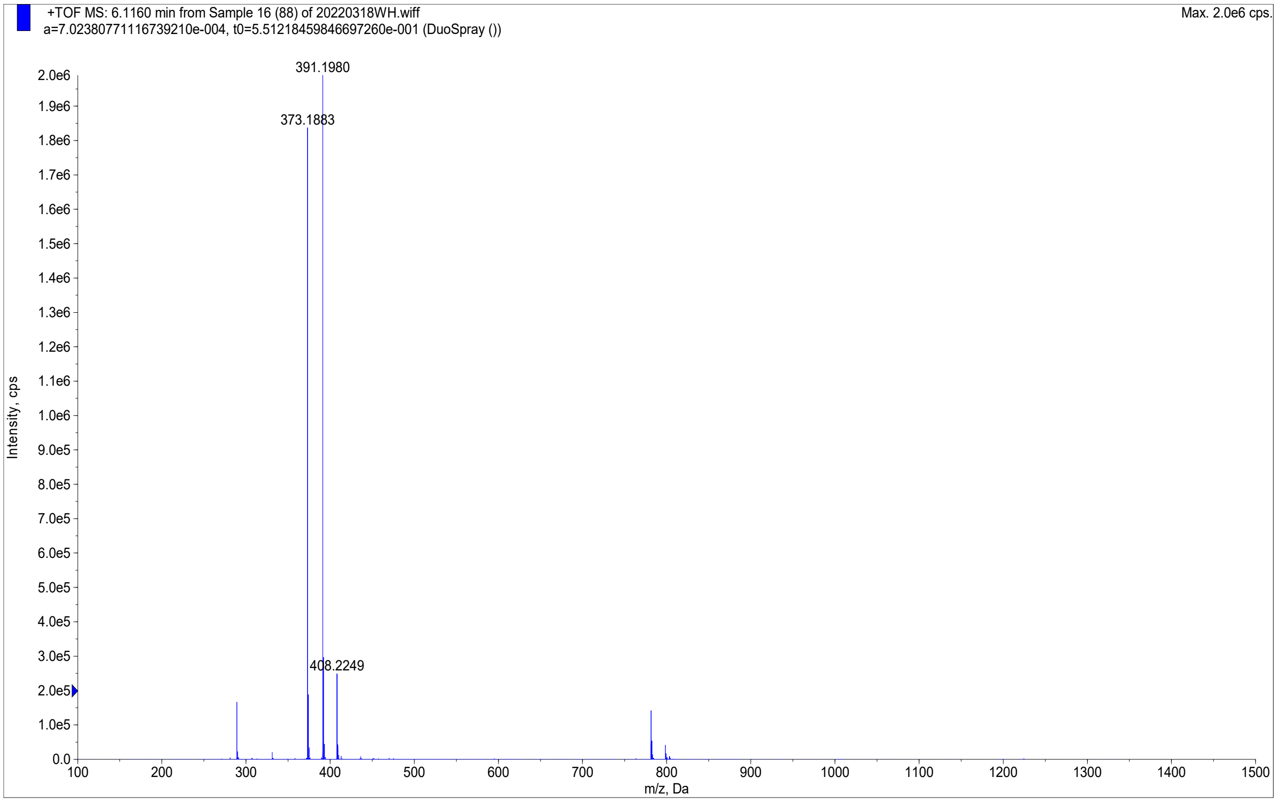

Figure S49. HR-ESI-MS spectrum of **7** in CD_3OD-*d4*

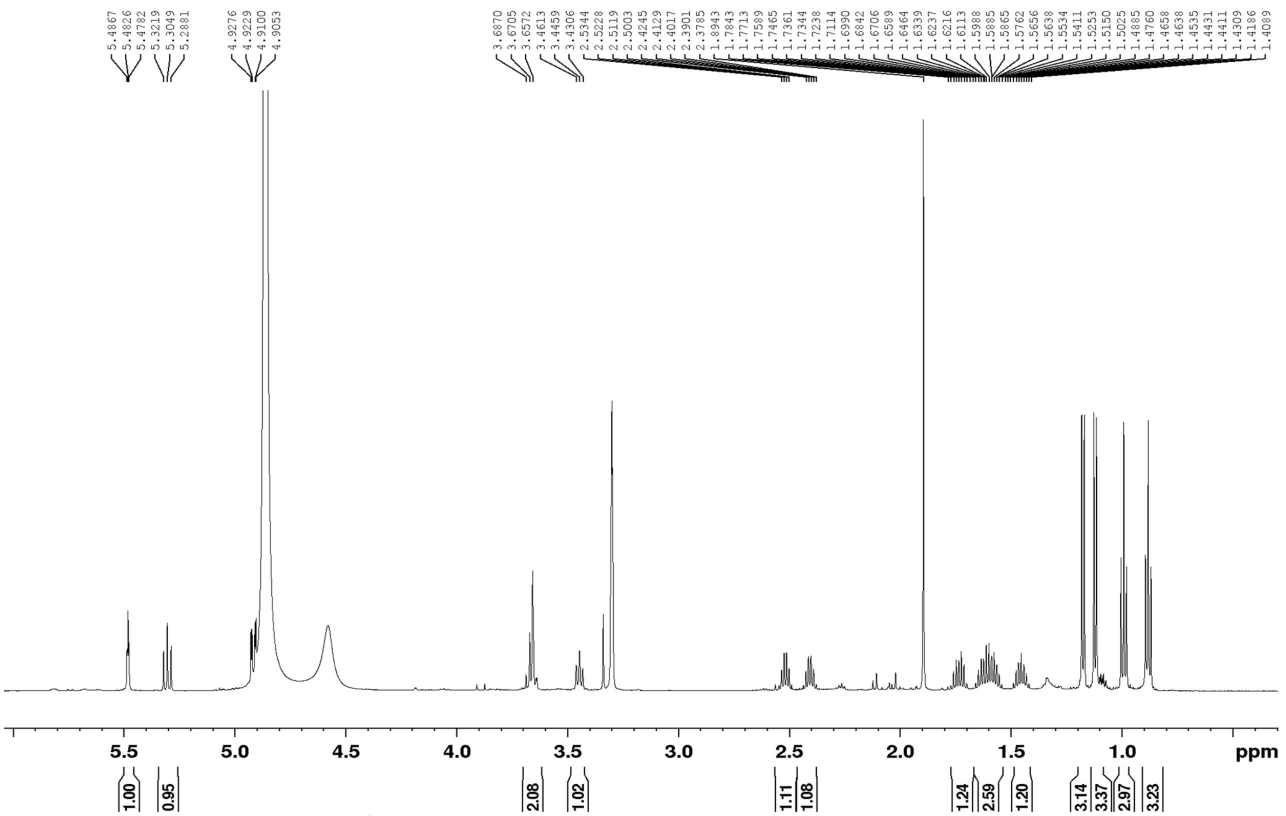

Figure S50. ^{1}H NMR spectrum (AV-600, 600 MHz) of **7** in CD_3OD-*d4*

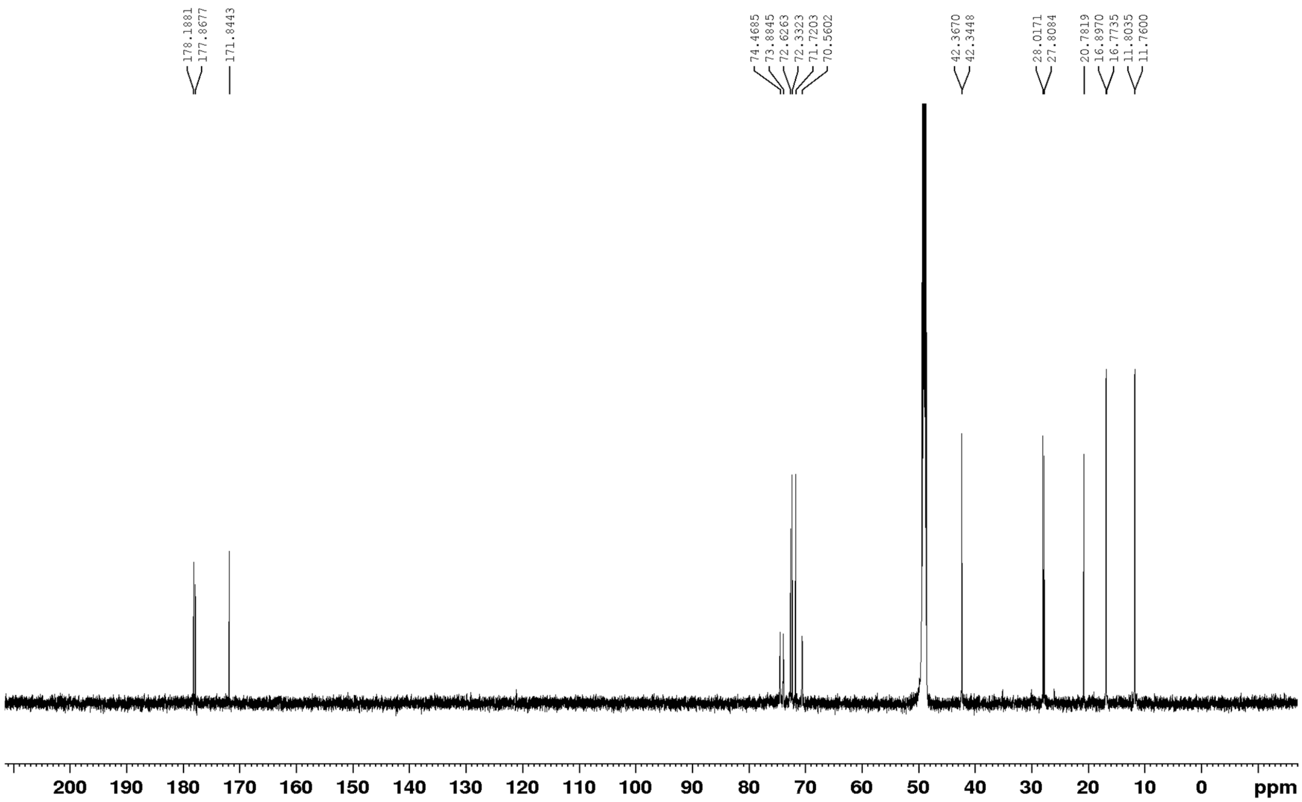

Figure S51. ^{13}C NMR spectrum (AV-600, 150 MHz) of **7** in CD_3OD-*d4*

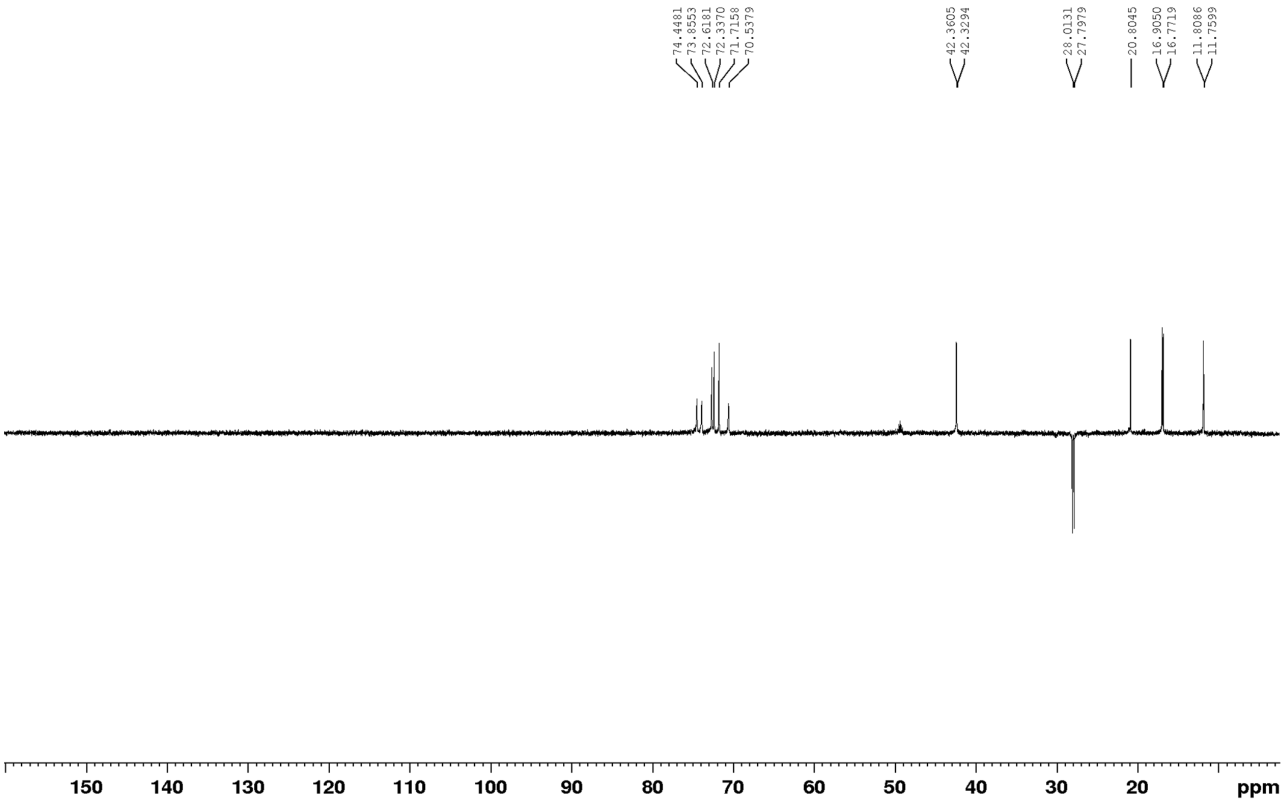

Figure S52. DEPT spectrum (AV-600) of **7** in CD_3OD-*d4*

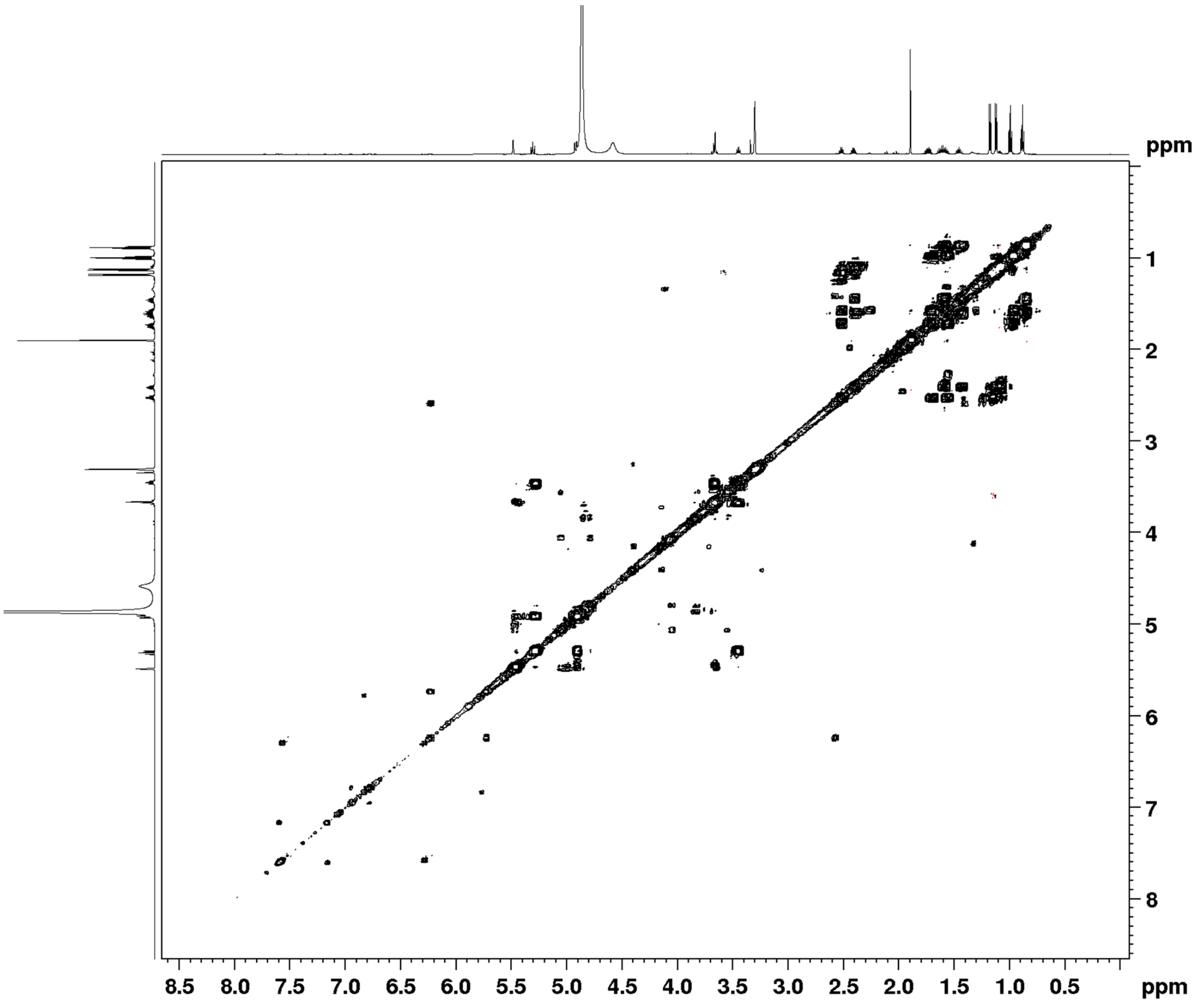

Figure S53. ^{1}H-^{1}H COSY spectrum (AV-600) of **7** in CD3OD-*d4*

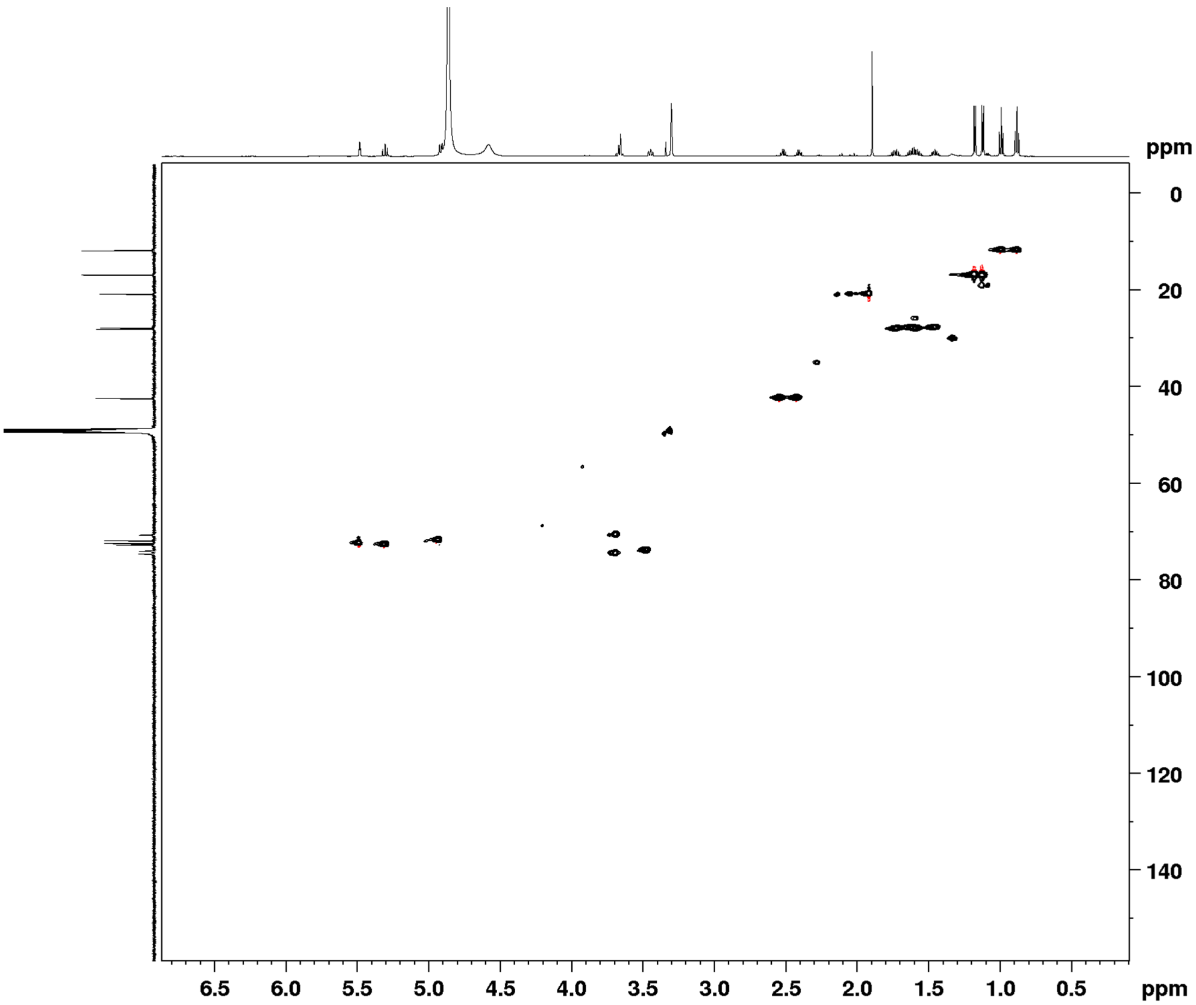

Figure S54. HSQC spectrum (AV-600) of **7** in CD_3OD-*d4*

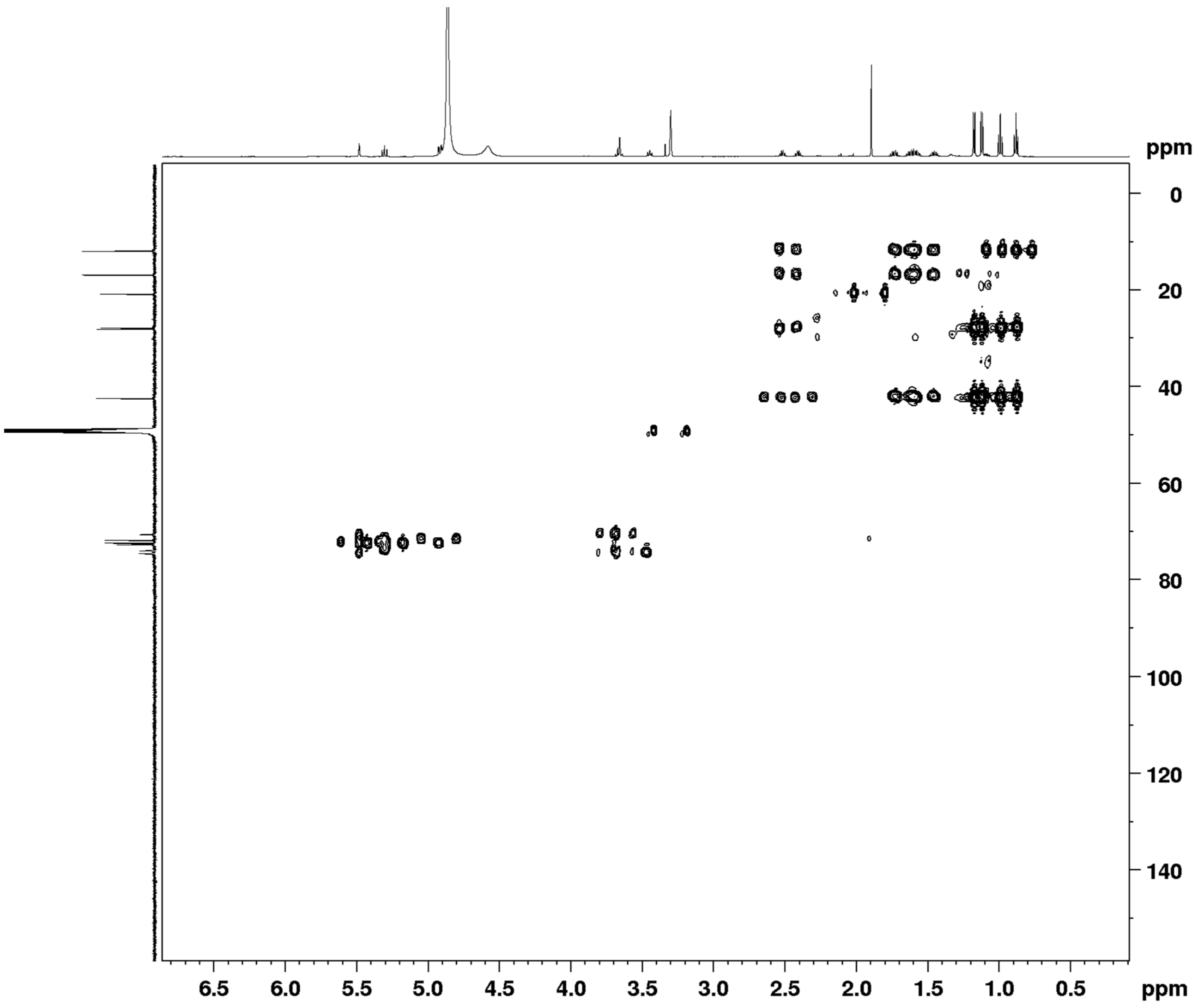

Figure S55. HMBC spectrum (AV-600) of **7** in CD_3OD-*d4*

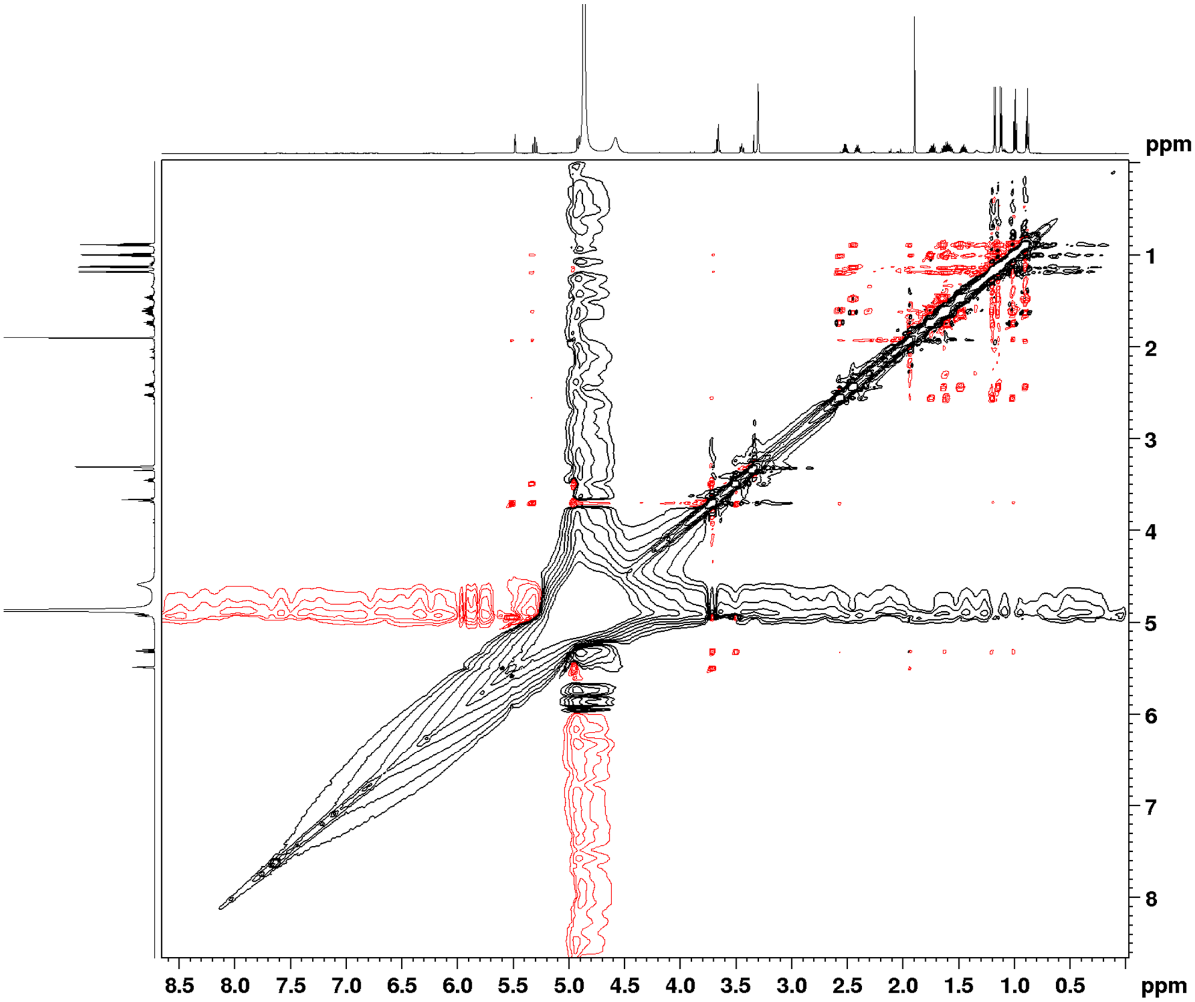

Figure S56. NOESY spectrum (AV-600) of **7** in CD_3OD-*d4*

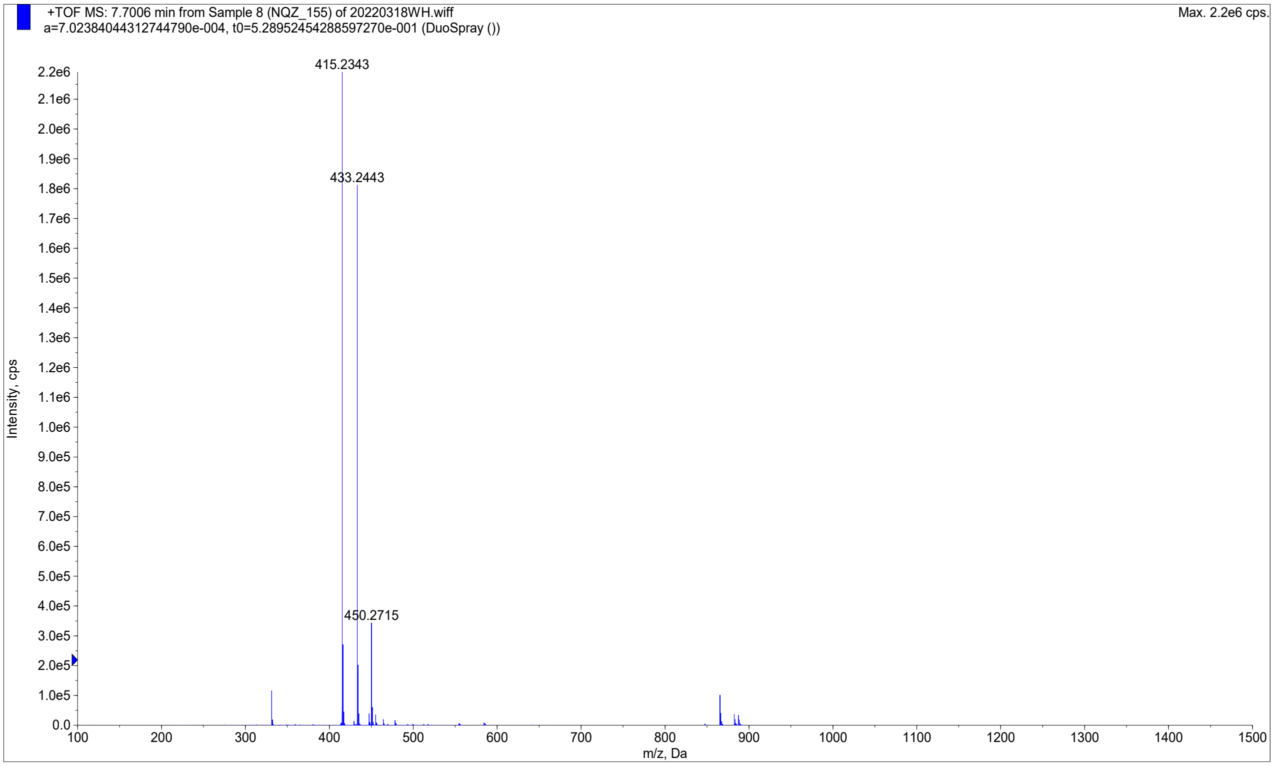

Figure S57. HR-ESI-MS spectrum of **8** in CD_3OD-*d4*

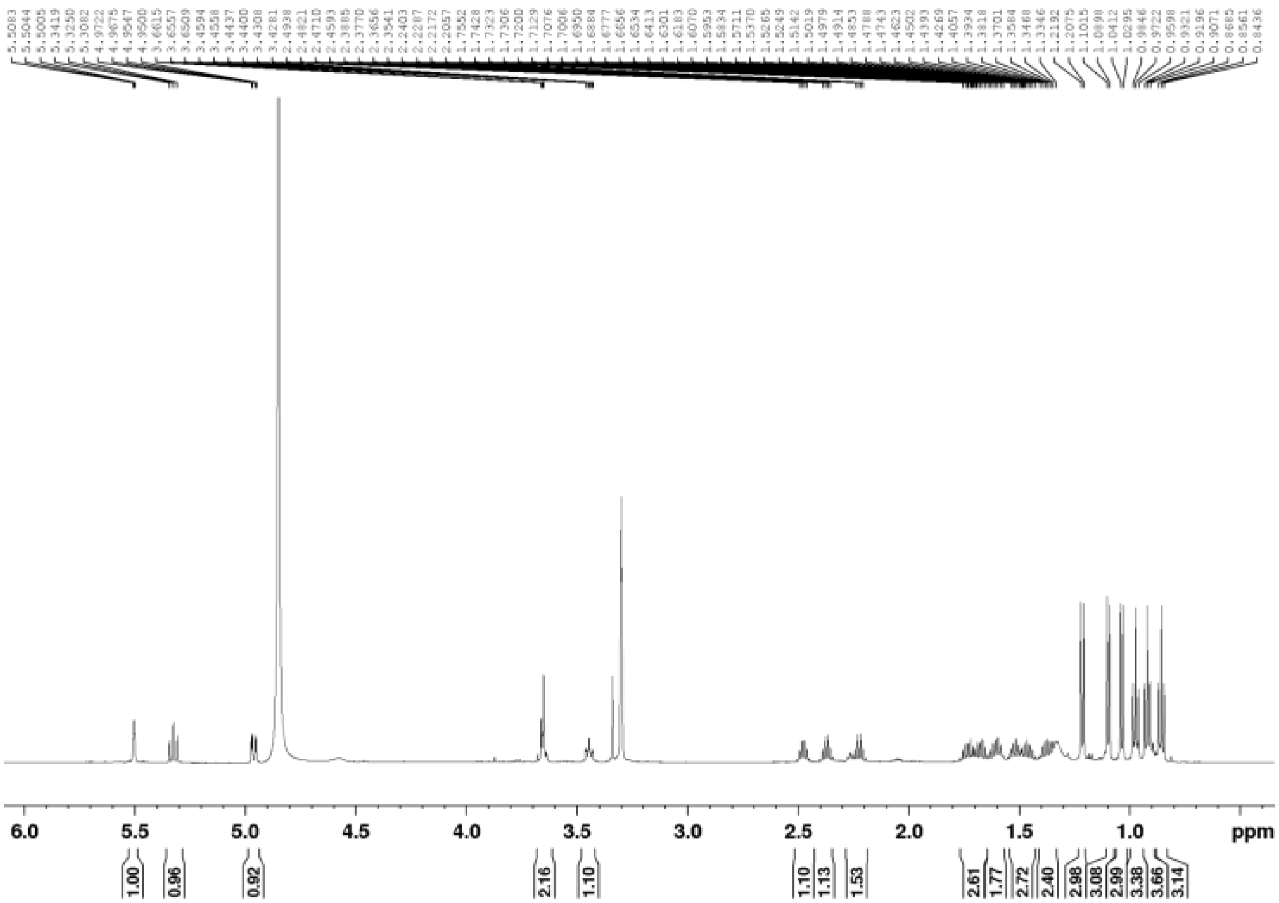

Figure S58. ^{1}H NMR spectrum (AV-600, 600 MHz) of **8** in CD_3OD-*d4*

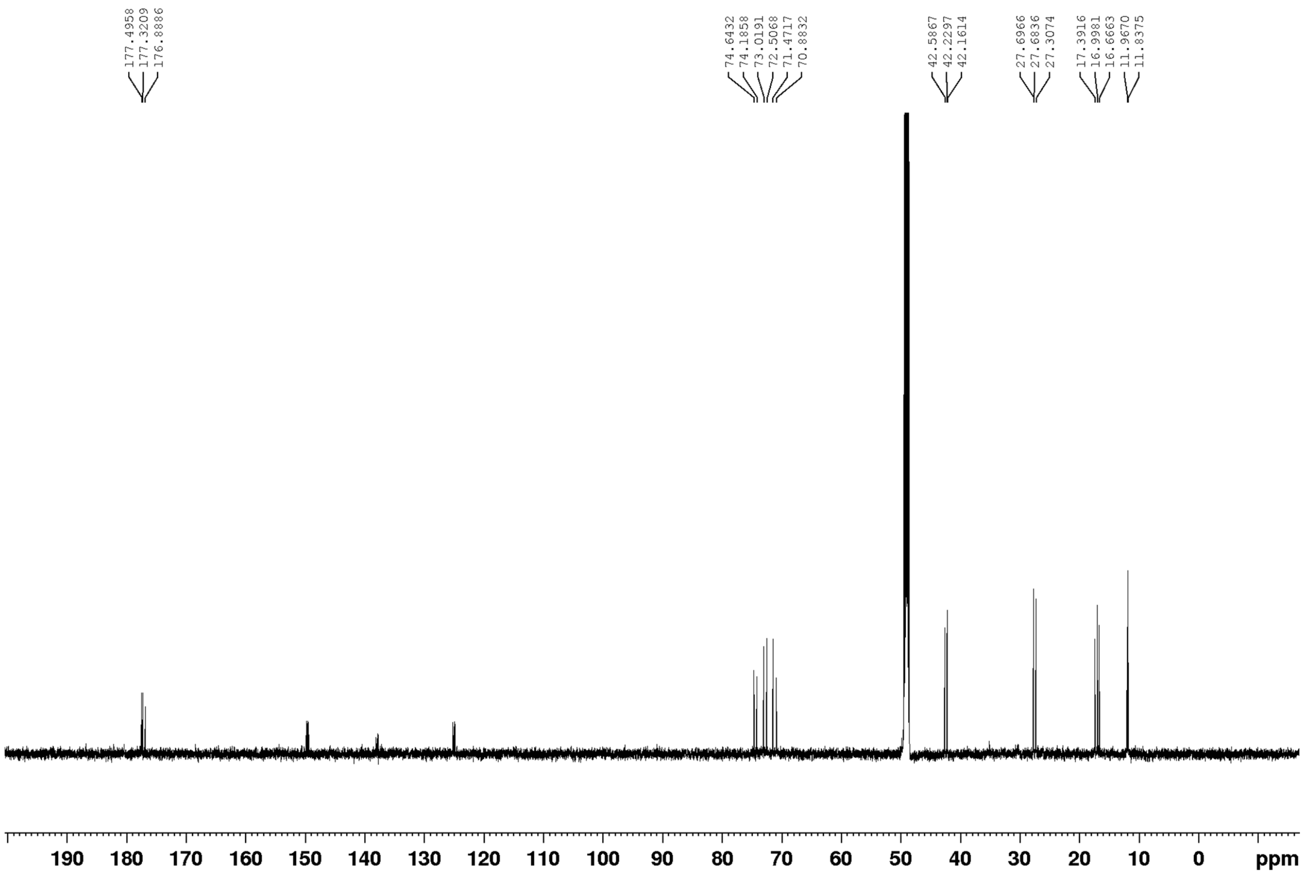

Figure S59.^{13}C NMR spectrum (AV-600, 150 MHz) of **8** in CD3OD-*d4*

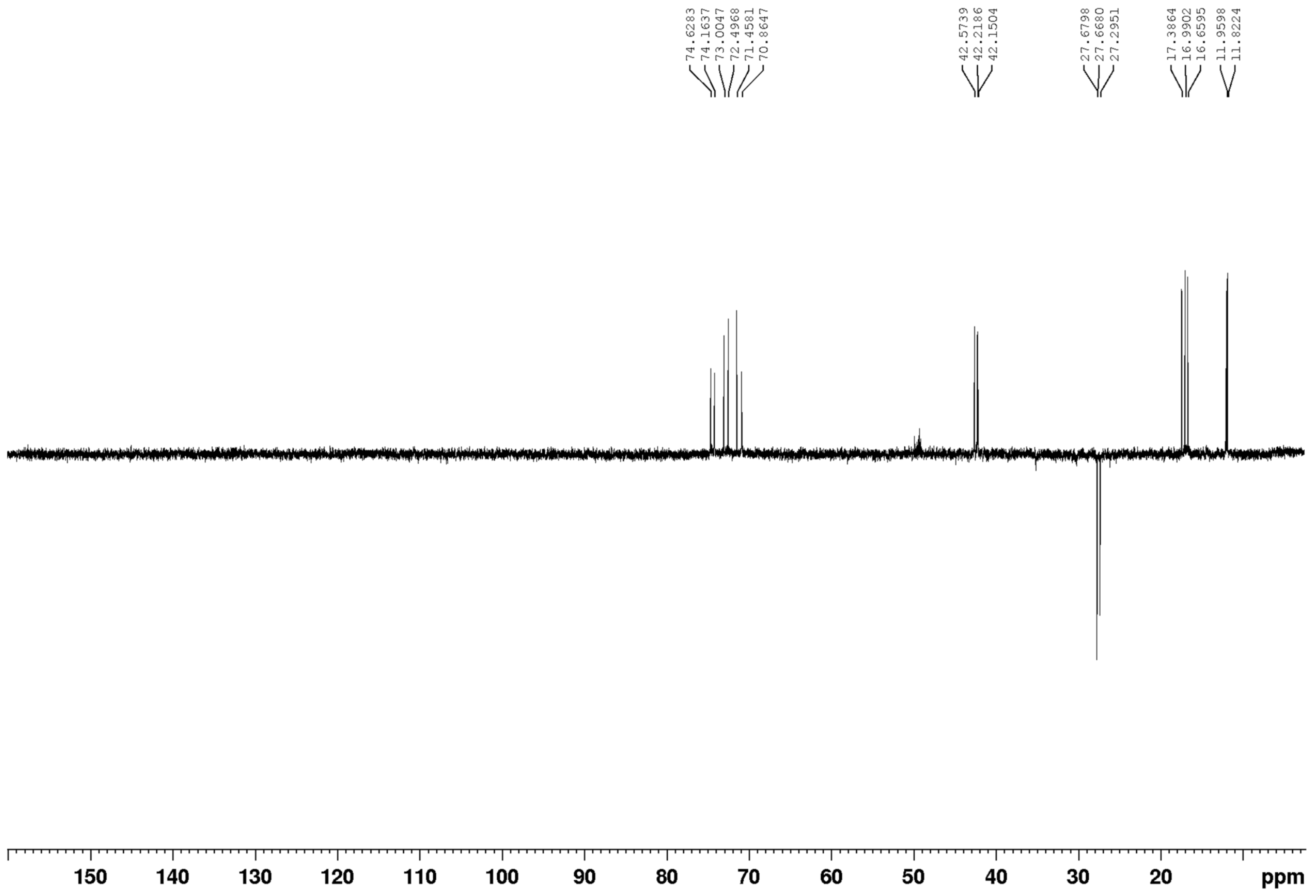

Figure S60. DEPT spectrum (AV-600) of **8** in CD_3OD-*d4*

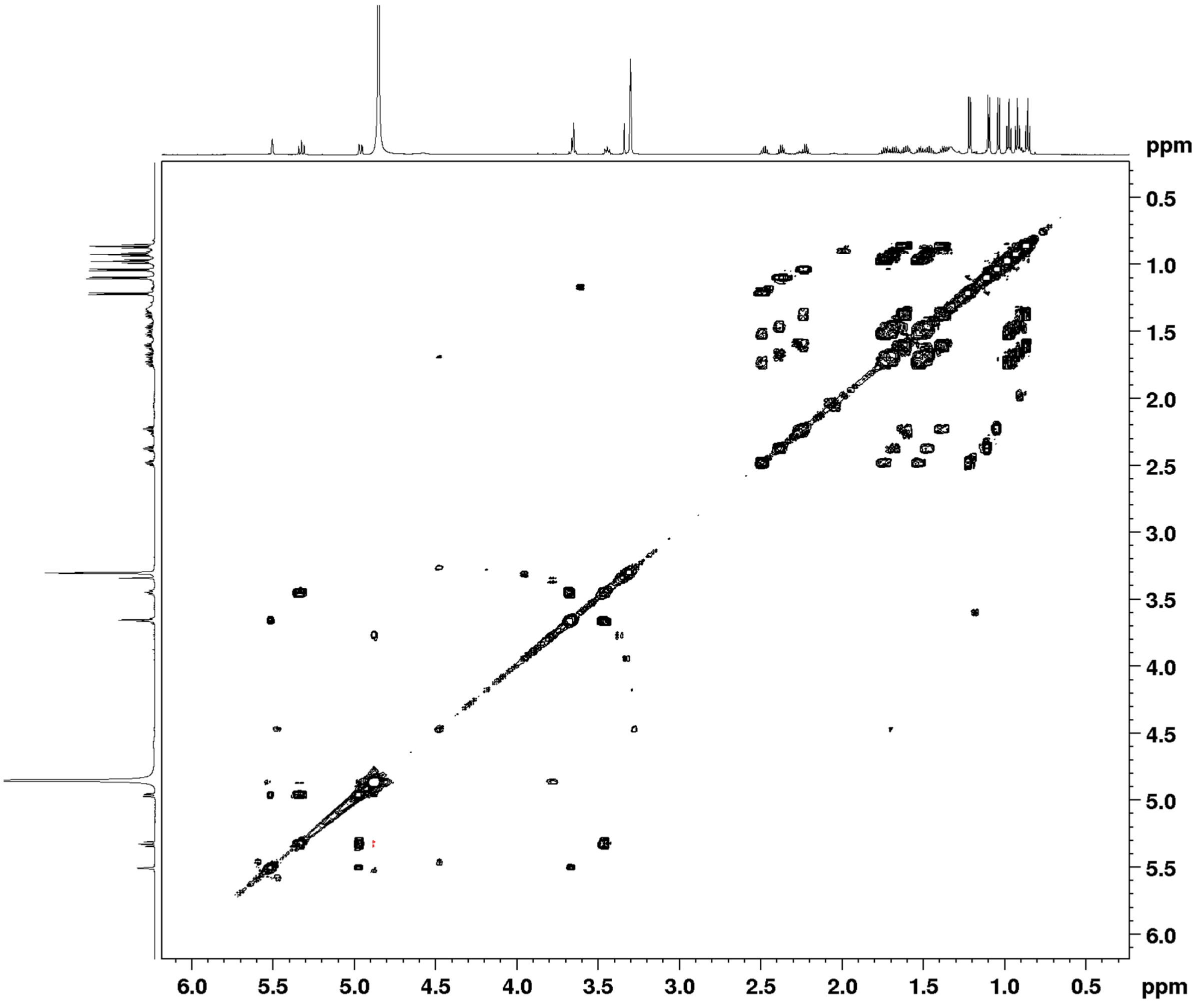

Figure S61. ^{1}H-^{1}H COSY spectrum (AV-600) of **8** in CD_3OD-*d4*

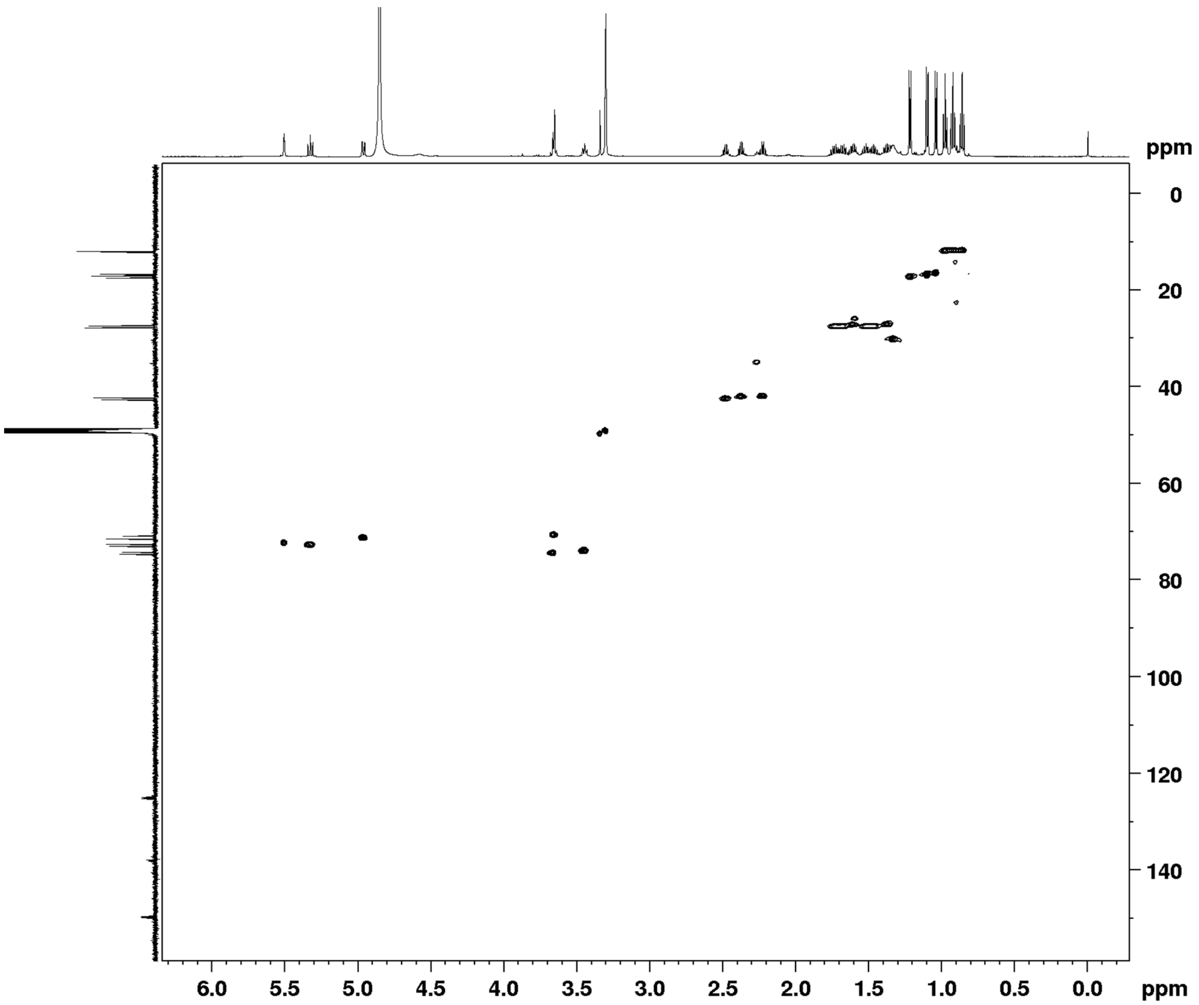

Figure S62. HSQC spectrum (AV-600) of **8** in CD_3OD-*d4*

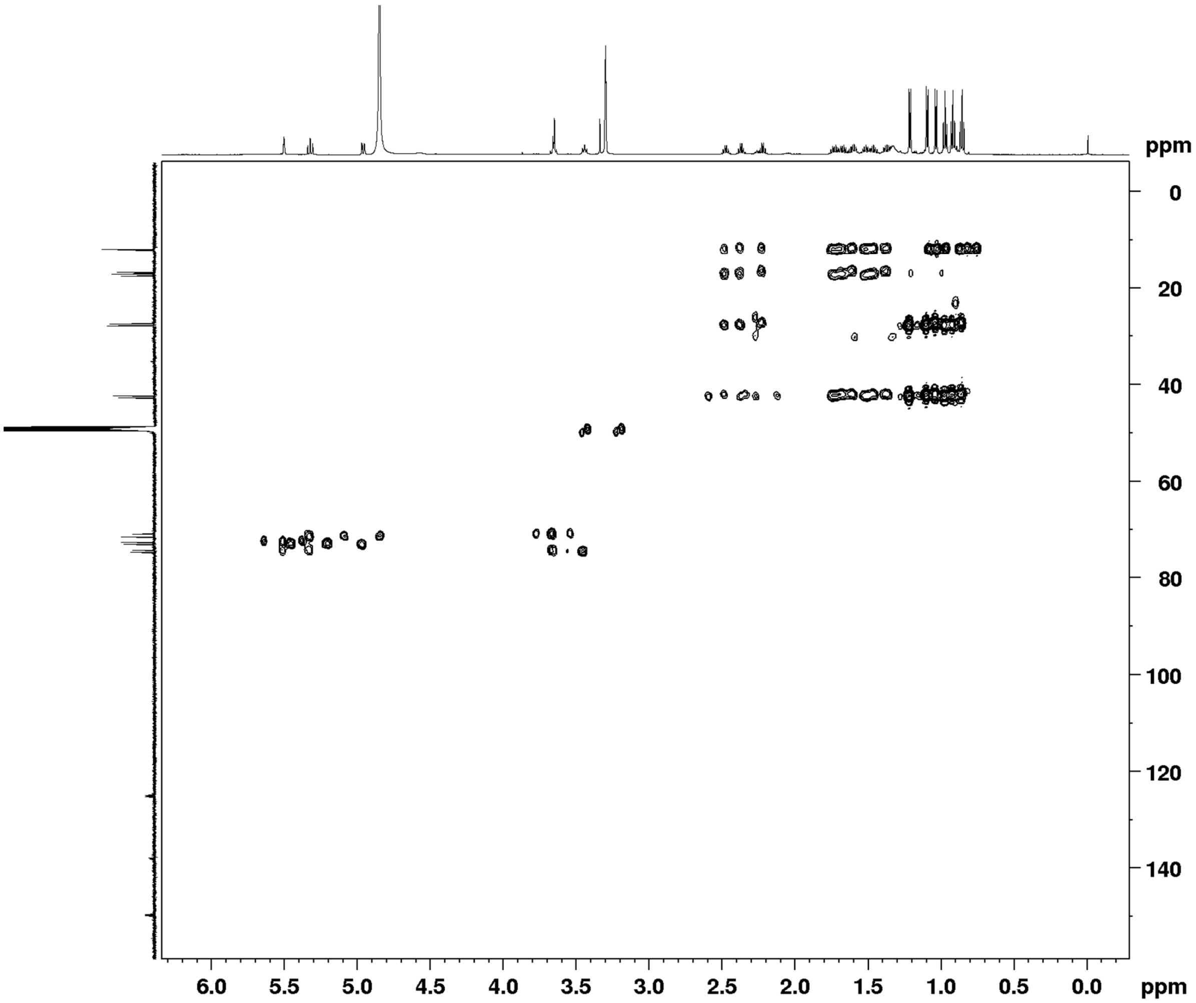

Figure S63. HMBC spectrum (AV-600) of **8** in CD3OD-*d4*

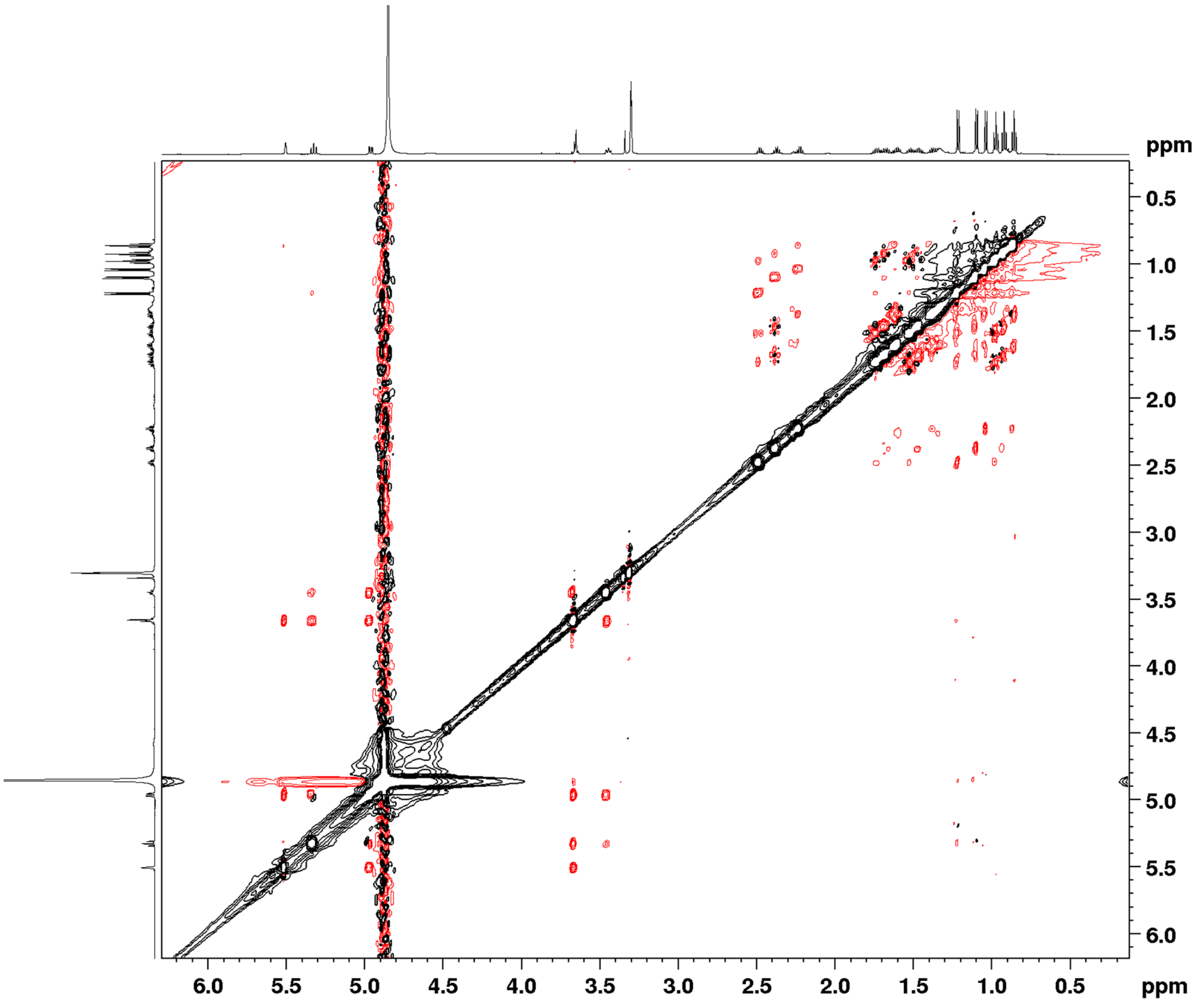

Figure S64. NOESY spectrum (AV-600) of **8** in CD_3OD-*d4*

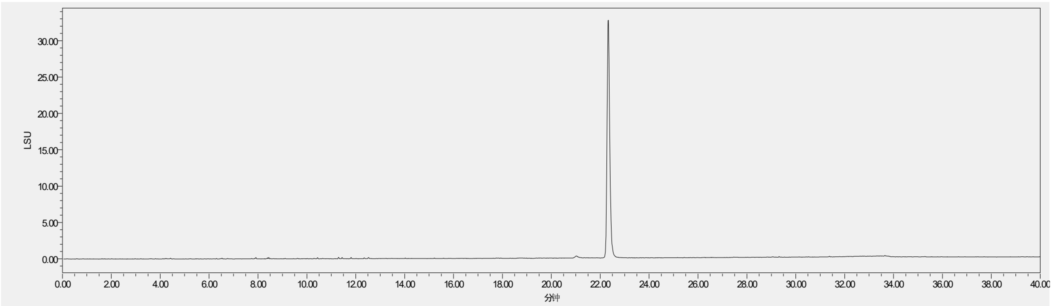

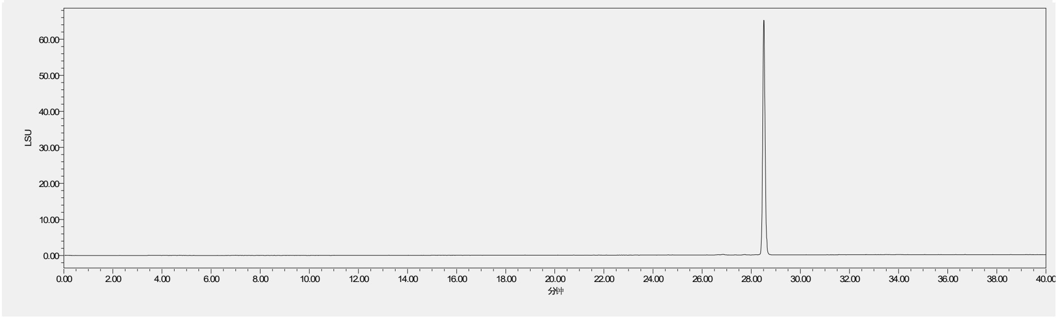

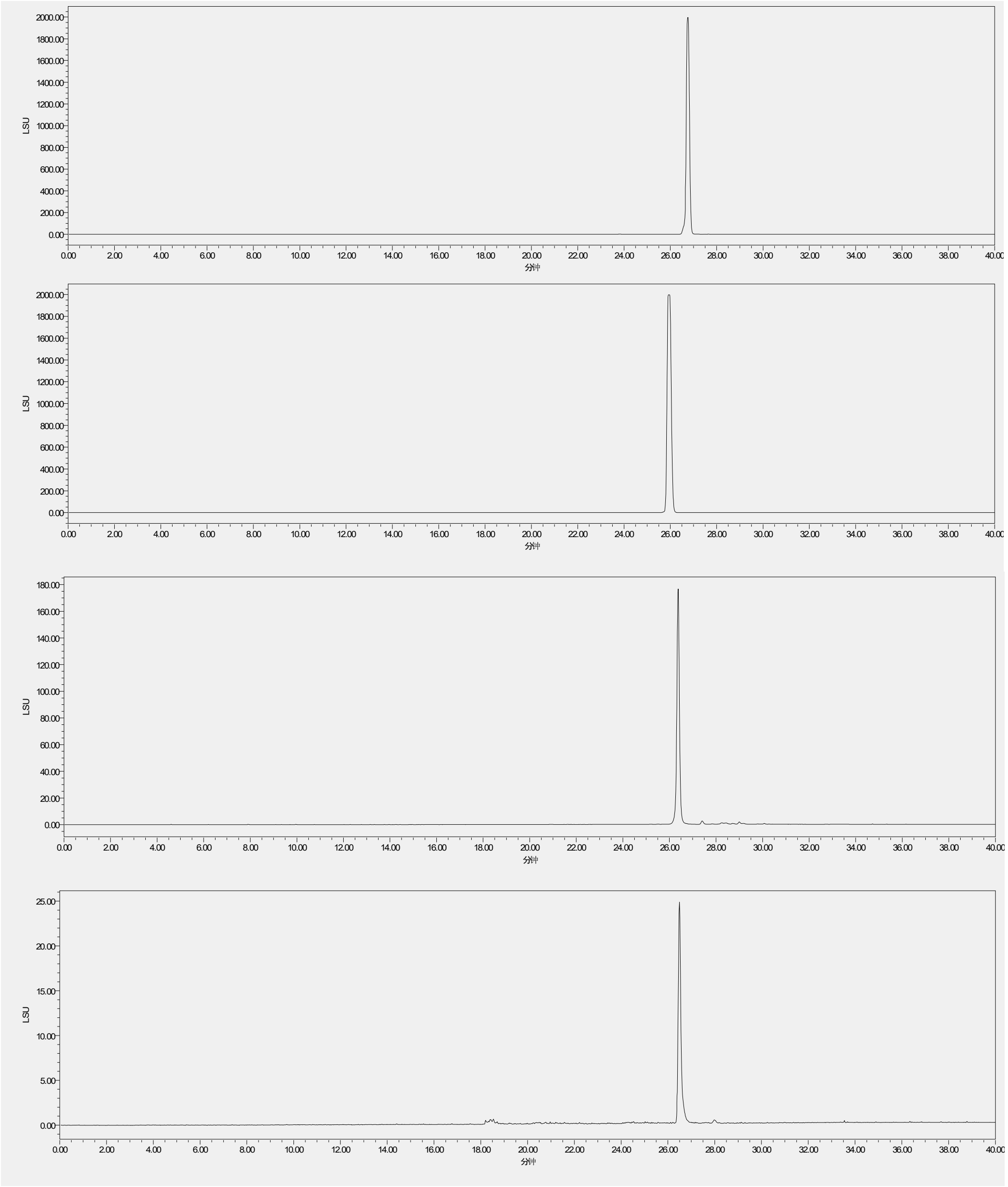
LSU
分钟
LSU
分钟
LSU
分钟
LSU
分钟

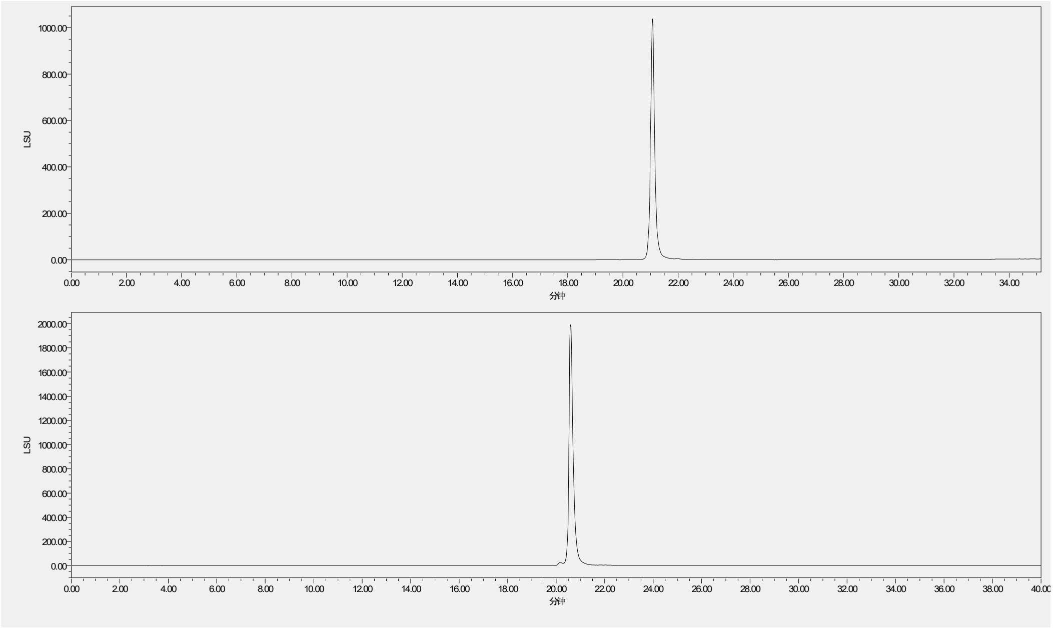

Figure S65. HPLC spectrum of **1**–**8**
